# Elucidating the hypoxic stress response in barley (*Hordeum vulgare* L.) during waterlogging: A proteomics approach

**DOI:** 10.1038/s41598-018-27726-1

**Published:** 2018-06-25

**Authors:** Haiye Luan, Huiquan Shen, Yuhan Pan, Baojian Guo, Chao Lv, Rugen Xu

**Affiliations:** 1 Jiangsu Key Laboratory of Crop Genetics and Physiology/Co-Innovation Center for Modern Production Technology of Grain Crops, Key Laboratory of Plant Functional Genomics of the Ministry of Education, Barley Research Institution of Yangzhou University, Yangzhou University, Yangzhou, 225009 China; 2 Institute of Agricultural Science in Jiangsu Coastal Areas, Yancheng, 224002 China

## Abstract

Waterlogging is one of the major abiotic stresses that affects barley production and yield quality. Proteomics techniques have been widely utilized to explore the mechanisms involved in the responses to abiotic stress. In this study, two barley genotypes with contrasting responses to waterlogging stress were analyzed with proteomic technology. The waterlogging treatment caused a greater reduction in biomass and photosynthetic performance in the waterlogging-sensitive genotype TF57 than that in the waterlogging-tolerant genotype TF58. Under waterlogging stress, 30, 30, 20 and 20 differentially expressed proteins were identified through tandem mass spectrometry analysis in the leaves, adventitious roots, nodal roots and seminal roots, respectively. Among these proteins, photosynthesis-, metabolism- and energy-related proteins were differentially expressed in the leaves, with oxygen-evolving enhancer protein 1, ATP synthase subunit and heat shock protein 70 being up-regulated in TF58. Pyruvate decarboxylase (PDC), 1-amino cyclopropane 1-carboxylic acid oxidase (ACO), glutamine synthetase (GS), glutathione S-transferases (GST) and beta-1, 3-glucanase in adventitious, nodal and seminal roots were more abundant in TF58 than those in TF57 under waterlogging stress. Ten representative genes were selected for validation by qRT-PCR in different genotypes with known waterlogging tolerance, and the expression levels of three candidate genes (*PDC*, *ACO* and *GST*) increased in the roots of all genotypes in response to the waterlogging stress. These three genes might play a significant role in the adaptation process of barley under waterlogging stress. The current results partially determined the mechanisms of waterlogging tolerance and provided valuable information for the breeding of barley with enhanced tolerance to waterlogging.

## Introduction

Waterlogging, caused by excess water that decreases the oxygen content in the soil and the ability of the plant to absorb nutrients, affects crop growth and reduces annual crop yields^1^. Approximately 16% of land worldwide has been affected by waterlogging stress, resulting in severe economic losses^2^. Barley is relatively sensitive to waterlogging stress, with 20–25% yield losses occurring under waterlogging conditions worldwide^3^. The root system in the plants is the first organ that responds to waterlogging^4^. Waterlogging tolerance is a complex trait, both genetically and physiologically^5^. To adapt to waterlogging stress, plants have evolved many morpho**-**anatomical and physiological adaptations, such as the formation of adventitious roots, aerenchyma in roots and reactive oxygen species (ROS) scavenging by antioxidant enzymes after plant exposure to hypoxic conditions^6–8^. Nevertheless, the molecular mechanisms of plant responses to waterlogging stress are not yet understood completely. Thus, the identification of candidate genes and the proteins responsible for waterlogging tolerance in barley is imperative.

Proteomic technique coupled with mass spectrometry (MS) can detect translational and post-translational regulations of different proteins. These techniques have been widely applied in barley to explore the mechanisms involved in their response to abiotic stress, including salt, drought and low O_2_^9–11^. Proteins associated with waterlogging tolerance have been reported in maize^12^, soybean^13^, wheat^14^ and tomato^15^. A number of waterlogging-induced proteins were identified as being involved in photosynthesis, disease- or defense-related mechanisms, metabolic enzymes, molecular chaperones, cell wall biosynthesis and signaling pathways^11,16,17^. NADP-malic enzyme, glutamate decarboxylase, glutathione synthetase (GSH) dehydrogenase, GSH S-transferase and xyloglucan endotransglycosylase 6 accumulated in maize genotypes with waterlogging-tolerant genotypes under waterlogging stress^12^. Many proteins showed significant changes in the roots, hypocotyls and leaves of soybean seedlings under waterlogging stress^13^. It was reported that proteins related to glycolysis and fermentation, such as, alcohol dehydrogenase (ADH) and pyruvate decarboxylase (PDC), showed increased levels, whereas the levels of scavengers of reactive oxygen species (ROS), such as superoxide dismutase (SOD) and peroxidase (POD), were decreased during flooding^13^. Overexpression of the *PDC* gene in transgenic *Arabidopsis* resulted in increased ATP and NAD+ production and ultimately enhanced tolerance to waterlogging stress^18^. However, limited information is available about the specific waterlogging-induced proteins in barley.

In the present study, two barley genotypes with contrasting responses to waterlogging were analyzed using two**-**dimensional polyacrylamide gel electrophoresis (2**-**DE) coupled with MS. The primary objective was to identify the proteins and genes associated with waterlogging tolerance in barley. Five other genotypes with known waterlogging tolerance were used to validate the results.

## Results

### Plant growth responses to waterlogging stress

**Figure 1:**
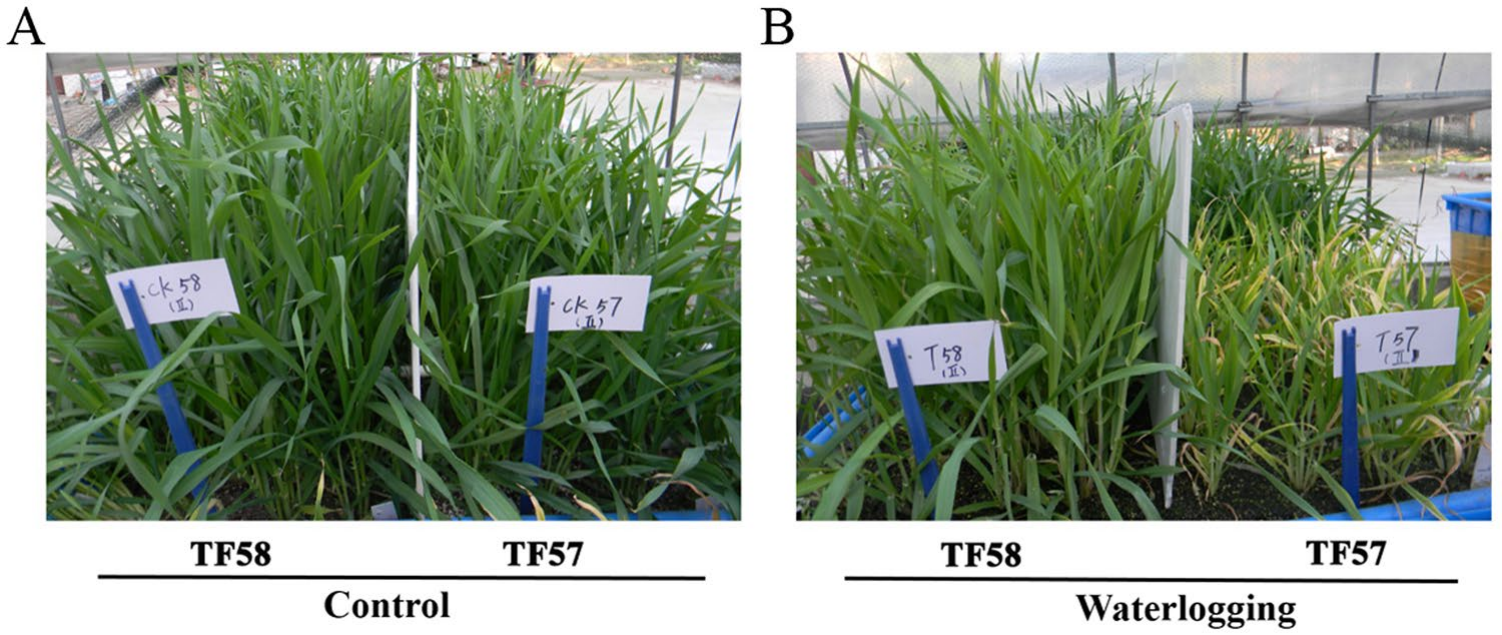
Growth performance of waterlogging-sensitive TF57 and waterlogging-tolerant TF58 under control and waterlogging treatment. The performance of TF57 (right) and TF58 (left) under control (**A**); Differential performance of TF57 (right) and TF58 (left) under waterlogging stress (**B**).

**Figure 2:**
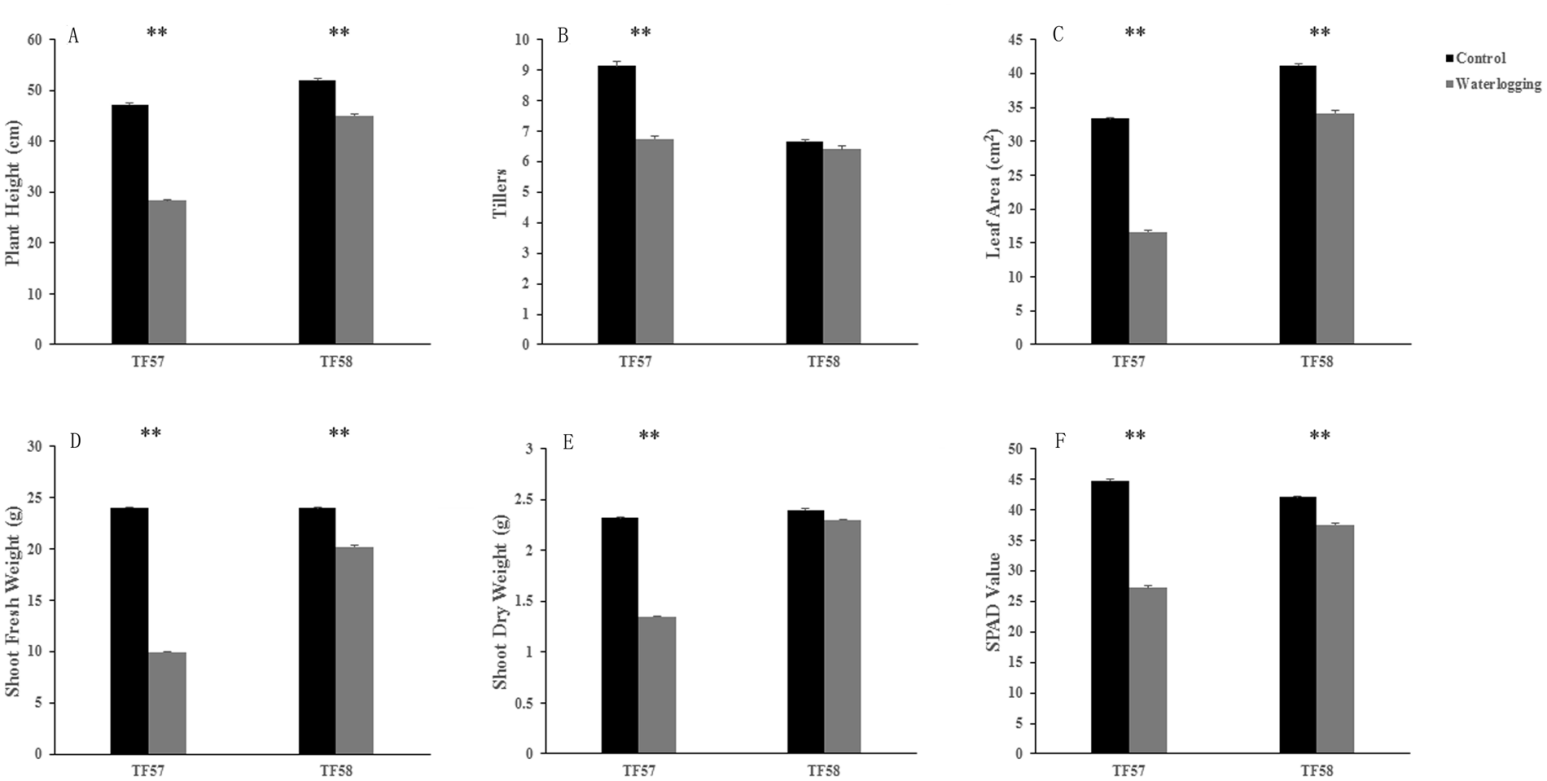
Comparison of the morphological traits of barley in response to waterlogging stress. Plant height (**A**); Tillers (**B**); Leaf Area (**C**); Shoot fresh weight (**D**); Shoot dry weight (**E**); and SPAD value (**F**). Five seedlings were randomly selected for measurement from each replication, and three independent biological experiments were performed. Each bar represents the mean ± SD of 15 seedlings. Black and grey bars represent the control and waterlogging treatments, respectively. Statistical analysis was performed using Student’s *t*-test as shown by asterisks, indicating significant differences between treatments (**p < 0.01).

As shown in Fig. 1, the growth of TF57 and TF58 was affected after the 21**-**day waterlogging treatment, and TF58 was more tolerant than TF57. The plant height, tillers, leaf area, shoot fresh weight and dry weight of TF57 decreased by 40%, 26%, 50%, 59%, and 42%, respectively, while the same parameters for TF58 were reduced by only 13%, 3%, 17%, 16%, and 4%, respectively, compared to those of the control (Fig. 2). The soil-plant analysis development (SPAD) value of TF57 (based on chlorophyll meter readings) decreased by 39.2% relative to the control, whereas that of TF58 was reduced by only 11.7% (Fig. 2).

### Physiological responses of TF57 and TF58 under waterlogging

**Figure 3:**
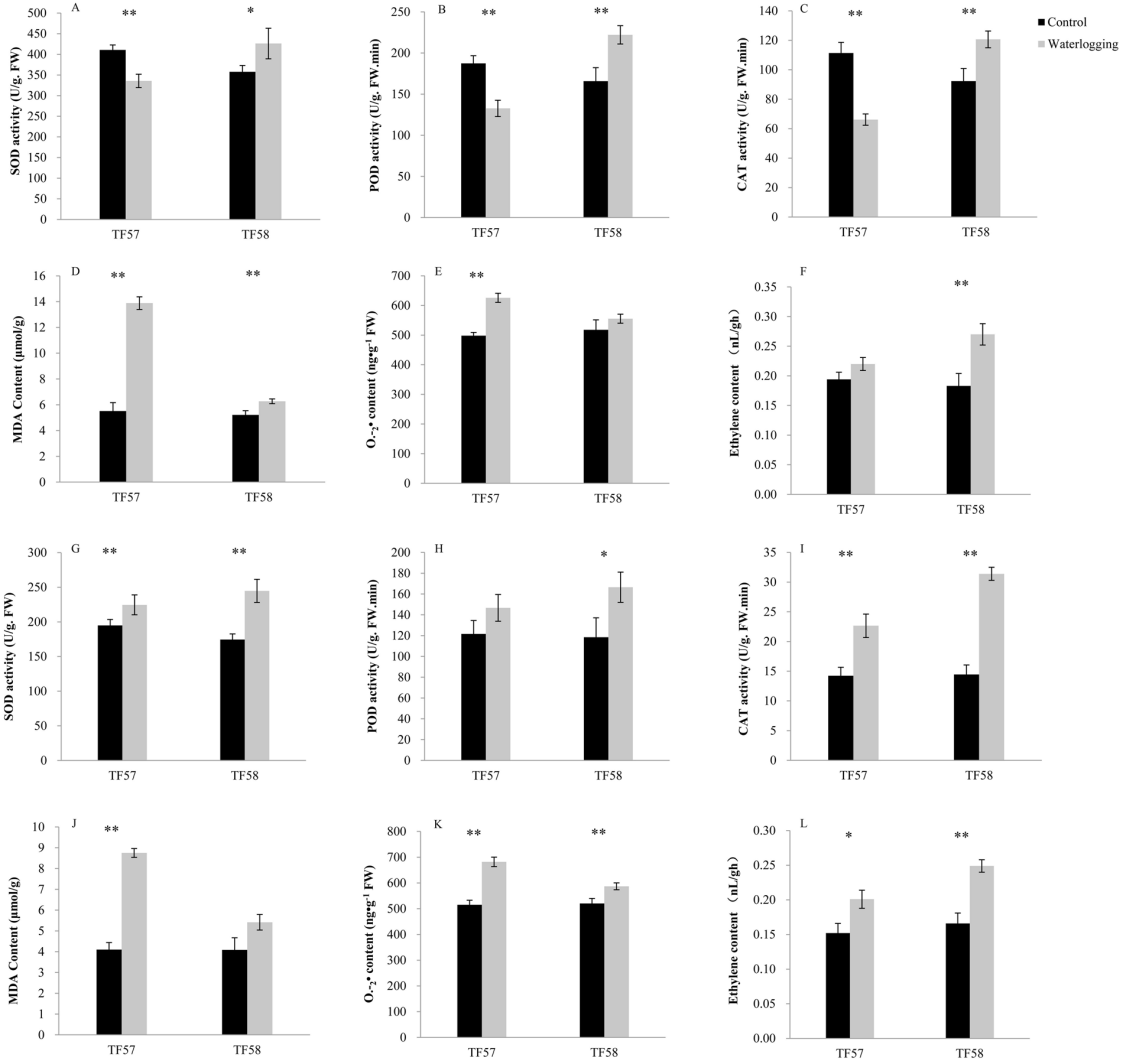
Effect of waterlogging on the superoxide dismutase (SOD) in the leaves (**A**) and roots (**G**); peroxidase (POD) in the leaves (**B**) and roots (**H**); catalase (CAT) in the leaves (**C**) and roots (**I**); malonaldehyde (MDA) in the leaves (**D**) and roots (**J**); superoxide radical (${{\rm{O}}}_{2}^{.-}$) content in the leaves (**E**) and roots (**K**); and ethylene content of the leaves (**F**) and roots (**L**). Three seedlings were randomly selected for measurement from each replication and three independent biological experiments were performed. The results are presented as the mean ± SD of nine seedling. Statistical analysis was performed using Student’s *t*-test. * And ** represent significant differences at *p* < 0.05 and *p* < 0.01, respectively.

Under waterlogging stress, significant differences were observed in the physiological response between TF57 and TF58. Antioxidant enzyme activities in the leaves of susceptible TF57 significantly decreased during waterlogging, while those of tolerant TF58 significantly increased (Fig. 3A–C). Significant increases in the antioxidant enzyme activities of the roots were observed in both genotypes under waterlogging stress (Fig. 3G–I). In addition, MDA an 98 d O_2_^.−^ concentrations increased significantly in the leaves and roots of TF57 but only slightly increased in the same tissues of TF58 under waterlogging conditions (Fig. 3D,E,G,K). In contrast, waterlogging led to a significant increase in the ethylene content, which was greater in TF58 than TF57 (Fig. 3F,L).

### Differential changes in proteins under waterlogging stress

Two-dimensional electrophoresis (2**-**DE) maps of different plant organs were constructed with three biological replicates to identify protein changes in the leaves, adventitious, seminal and nodal roots of two barley genotypes (TF57 and TF58) under waterlogging stress. With a linear gradient of pH values ranging from 4**–**7, 725, 922, 1158 and 856 reproducible protein spots were detected in all three biological replicates from the leaves (Fig. S1), adventitious roots (Fig. S2), nodal roots (Fig. S3) and seminal roots (Fig. S4). Principal component analysis (PCA) of the different organs was performed with all proteomic data, which clearly showed a separation of the groups (TF57, TF58 under control and waterlogging) (Fig. S5). Therefore, the PCA indicated that waterlogging induces significantly different responses in TF57 and TF58. The PCA analysis also implied high reproducibility among replicates.

**Figure 4:**
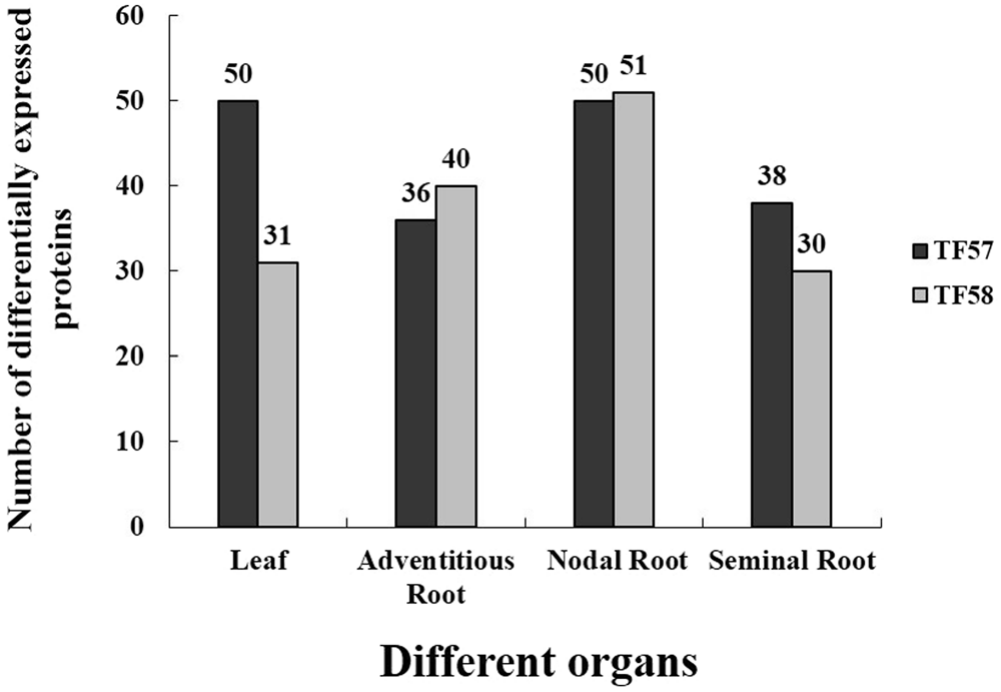
The number of differentially expressed proteins between control and waterlogging samples in the leaves, adventitious roots, nodal roots and seminal roots of TF57 and TF58. Black and grey bars represent the TF57 and TF58, respectively.

Quantitative analysis of protein expression was performed between waterlogging and control. Only protein spots with fold changes more than 1.5 or less than 0.66 (*p* < 0.05) were considered as differentially expressed proteins. Under waterlogging stress, 50 (30 increased and 20 decreased), 36 (15 increased and 21 decreased), 50 (32 increased and 18 decreased) and 38 (17 increased and 21 decreased) protein spots were significantly altered (*p* < 0.05) in the leaves, adventitious roots, nodal roots and seminal roots of TF57, relative to those in the control (Fig. 3). However, in the same organs, 31 (11 increased and 20 decreased), 40 (16 increased and 24 decreased), 51 (27 increased and 24 decreased) and 30 (13 increased and 17 decreased) protein spots, respectively, changed significantly (*p* < 0.05) in TF58 relative to those in the control (Fig. 4). Relatively fewer protein spots changed significantly (*p* < 0.05) in the leaves and seminal roots of TF58 than those of TF57 (Fig. 4).

### Identification and functional classification of waterlogging responsive proteins in different organs

**Figure 5:**
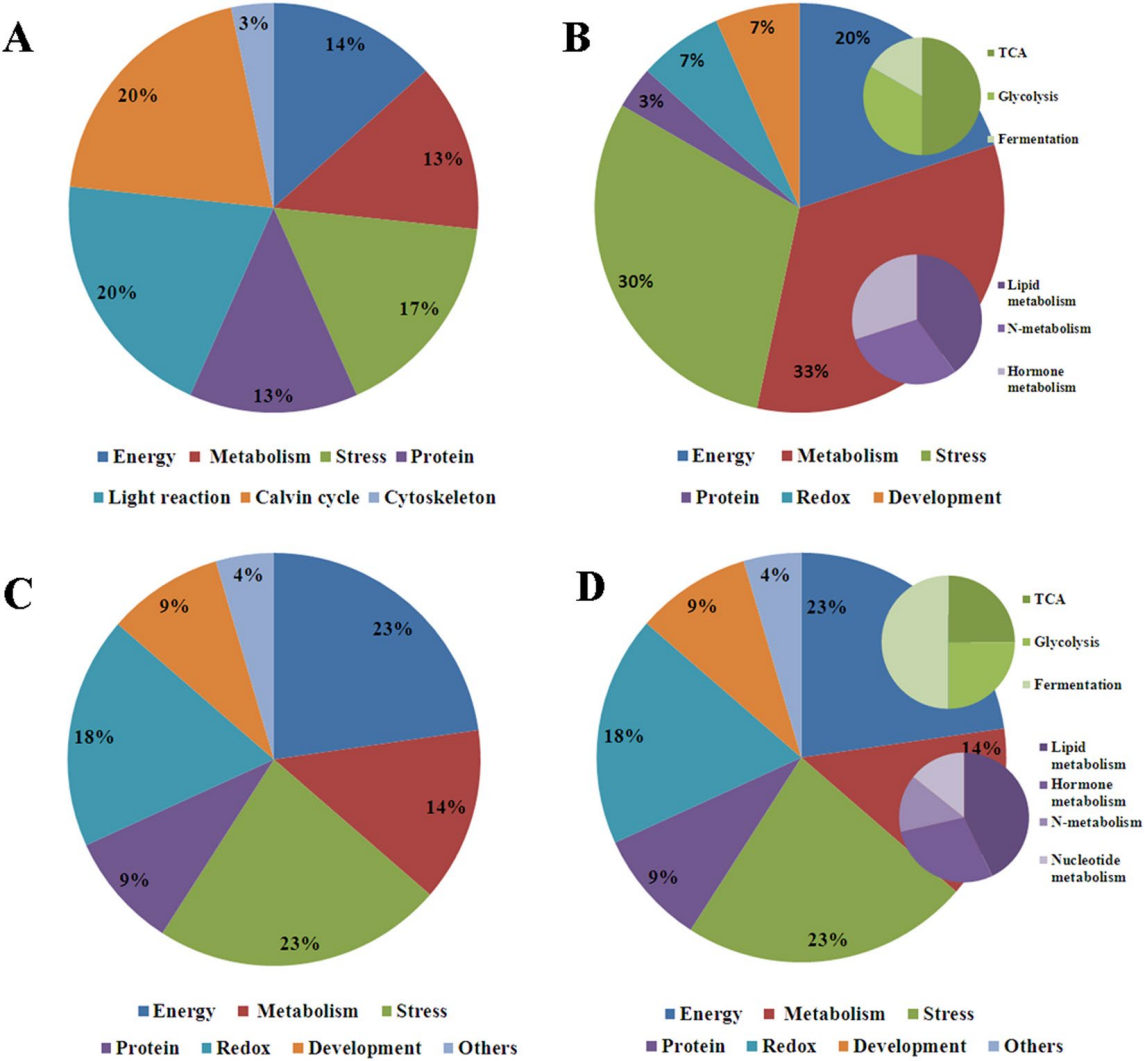
Functional categories of differentially expressed proteins in different organs. Leaves (**A**); adventitious roots (**B**); nodal roots (**C**); and seminal roots (**D**).

All differentially expressed protein spots between the control and waterlogged plants were excised from the representative 2**-**DE gels for protein identification. A total of 100 protein spots were successfully identified via tandem MS with 30, 30, 20 and 20 protein species in the leaves, adventitious roots, nodal roots and seminal roots, respectively (Tables S2, S3, S4 and S5). These protein spots were grouped into different categories according to their biological functions. In the leaves, the differentially expressed protein spots were grouped into seven categories (Fig. 5A). Most of the proteins identified were light reaction-related proteins (20%) and Calvin cycle-related proteins (20%). The remaining proteins were related to energy, metabolism, stress and the cytoskeleton.

In the adventitious roots, the differential protein spots were grouped into six categories, and most of the proteins identified were metabolism-related proteins. The remaining proteins were related to energy, stress and other functions (Fig. 5B).

In the nodal roots, the differentially expressed protein spots were classified into seven categories, with most of the proteins associated with the energy (23%). The other proteins were related to metabolism, stress, redox, development and other functions (Fig. 5C).

As shown in Fig. 5D, the identified proteins in the seminal roots were classified into seven groups according to function, including energy (23%), metabolism (14%), stress (23%), protein (9%), redox (18%), development (9%) and other functions (4%).

Several proteins were identified in multiple spots and located at different positions. For example, in the adventitious roots, spots AR595 and AR17 were identified as NADP**-**dependent malic enzymes (Fig. S2). In the nodal roots, spots NR287 and NR275 were identified as beta**-**1,3**-**glucanase 2a (Fig. S3). The tandem mass spectrometry (MS/MS) results revealed that the positional variation was a consequence of post-translational modification (oxidation) (Tables S3 and S4).

### Genotypic differences in the proteomes responding to waterlogging stress

**Figure 6:**
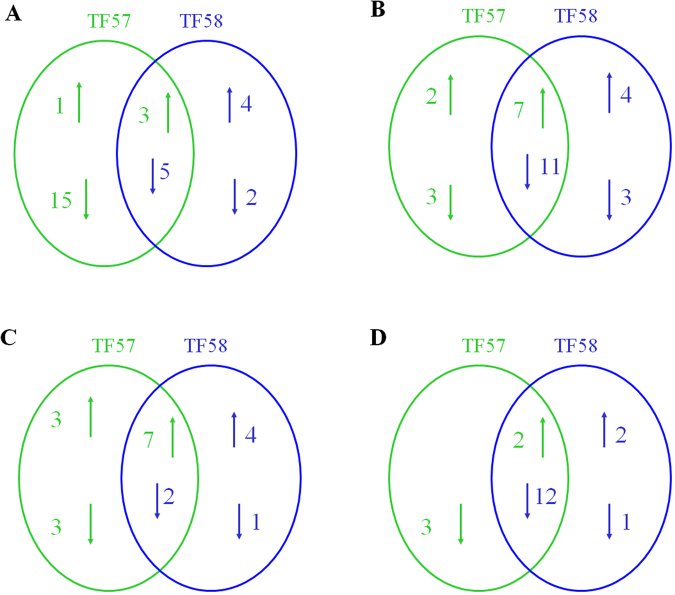
The number of differentially expressed protein spots successfully identified by tandem MS in different organs. Leaves (**A**); adventitious roots (**B**); nodal roots (**C**); and seminal roots (**D**).

Different mechanisms were observed in response to waterlogging in TF57 and TF58. In the leaves, six differentially abundant proteins (DAPs) were identified only in TF58. Sixteen DAPs were identified in TF57, and most of these DAPs showed significant down-regulation compared to the control. Eight DAPs were found in both genotypes (Fig. 6A). The number of down**-**regulated protein spots in TF57 was significantly greater than in TF58. L176 (oxygen**-**evolving enhancer protein 1), L9 (ATP synthase subunit mitochondrial-like), L66 (adenosine diphosphate glucose pyrophosphatase), and L357 (heat shock protein 70) were up-regulated in TF58 but remained unchanged in TF57. The differences in the responses of these proteins in the leaves could partially explain the more integrated morpho**-**anatomical structures and photosynthesis systems in TF58 during waterlogging (Table S2).

In the adventitious roots, seven DAPs were identified in TF58, five in TF57, and eighteen in both genotypes (Fig. 6B). Spots AR595, AR17 (NADP**-**dependent malic enzyme), AR370 (caffeic acid o**-**methyltransferase), and AR321 (guanine nucleotide**-**binding protein subunit beta) were up-regulated in TF58 but remained unchanged in TF57. The abundance of spots AR373 (glutamine synthetase isoform GS1_2) and AR852 (1-aminocyclopropane**-**1**-**carboxylate oxidase 1**-**like) were significantly increased in both genotypes, and the fold changes of these proteins in TF58 were significantly higher than those in TF57. Spots AR689 (quinone reductase 2) and AR144 (translationally**-**controlled tumour protein) were up**-**regulated in TF57 but down-regulated in TF58 (Table S3).

Five DAPs were identified in the nodal roots of TF58, six in TF57, and nine DAPs shared between both genotypes (Fig. 6C). NR864 (pyruvate decarboxylase), NR709 (NADP**-**dependent malic enzyme), NR1032 (ATP synthase d mitochondrial), and NR176 (glutathione s**-**transferase 3**-**like) were up**-**regulated only in TF58. The protein abundance of NR412 (1**-**aminocyclopropane**-**1**-**carboxylate oxidase 1**-**like), NR997 (peroxidase), NR275 and NR287 (beta**-**1,3**-**glucanase 2a) was enriched in both genotypes, but the change in TF58 was greater than that of TF57 (Table S4).

In the seminal roots, three DAPs were identified in TF58, three in TF57, and fourteen shared between genotypes (Fig. 6D). The abundance of SR101 (glutathione transferase f4) and SR86 (glutathione transferase) was enhanced in TF58. SR565 (pyruvate decarboxylase), SR160 (1**-**aminocyclopropane**-**1**-**carboxylate oxidase 1**-**like), and SR171 (glutamine synthetase isoform GS1_2) were enhanced in both genotypes, but the fold change of TF58 was higher than that in TF57. The different regulation patterns of the response proteins between the two genotypes could contribute to the variable waterlogging tolerances of TF57 and TF58 (Table S5).

### Validation of protein expression by immunoblotting and qRT-PCR analysis

**Figure 7:**
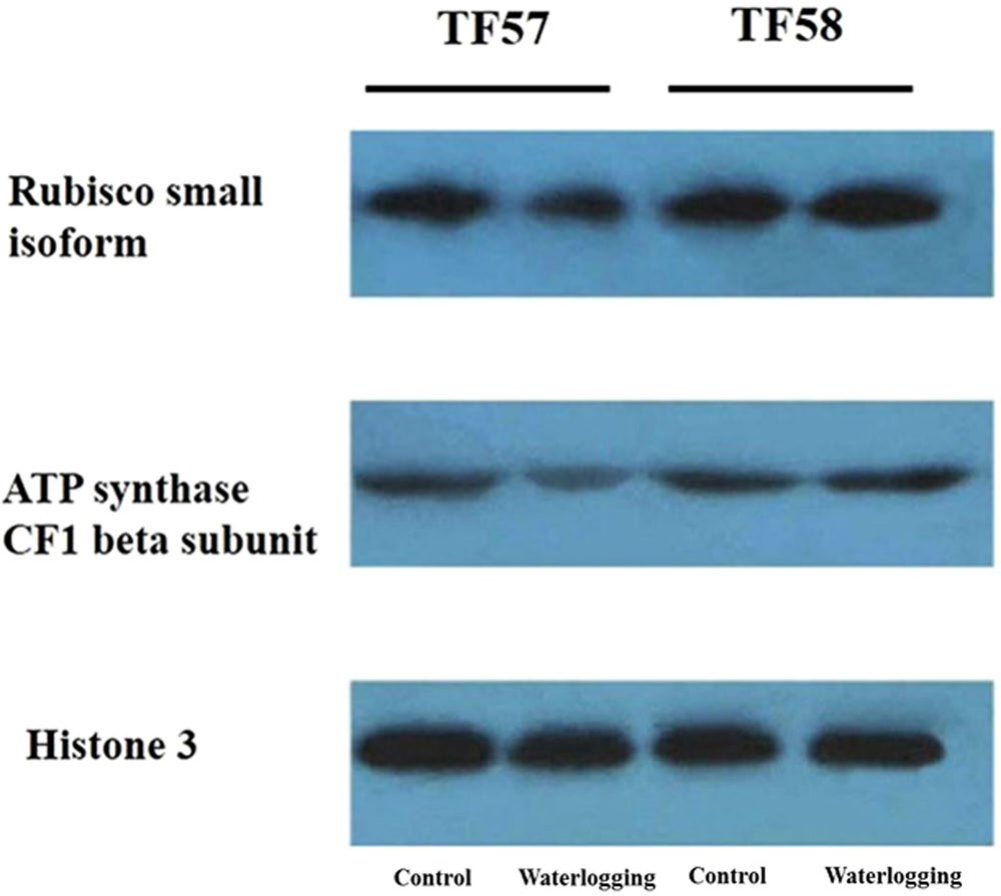
Western blotting analysis using Rubisco small isoform and ATP synthase beta subunit antibodies. Histone H3 detection was used as the control.

The protein expression revealed by 2**-**DE was confirmed by Western blotting analysis (Fig. 7). Similar protein abundance was found for the Rubisco small subunit (L263) when the results from the 2**-**DE and Western blotting analysis were compared. Under waterlogging, a lower abundance of the Rubisco small subunit was detected in TF57 than that in TF58. Similar observations were made using antisera against the beta subunit of ATP synthase (L4).

Ten proteins were selected for qRT**-**PCR analysis to assess the validity of the proteomic analysis. As shown in Fig. S6, the mRNA expression levels of genes encoding the ATP synthase CF1 beta subunit and the Rubisco activase small isoform significantly decreased in TF57 and TF58, with the changes being significantly greater in TF57. The expression levels of oxygen-evolving enhancer protein and cytosolic malate dehydrogenase were up-regulated in TF58 but down-regulated in TF57. As shown in Fig. S7, the transcript levels of *PDC*, *GS1*, 1**-**amino cyclopropane 1**-**carboxylic acid oxidase (*ACO*), glutathione S**-**transferases (GSTs), caffeic acid o**-**methyltransferase (*COMT*) and G**-**protein subunit beta were significantly up-regulated or remained unchanged in the adventitious roots, nodal roots and seminal roots of both barley genotypes under waterlogging stress, with TF58 showing generally higher expression levels. Overall, the mRNA expression levels of these genes, except for ATP synthase CF1 beta subunit in TF58 were correlated with protein abundance. This result indicated that the enrichment of these proteins likely resulted from the transcriptional induction of the corresponding genes under waterlogging stress.

### Further validation of candidate proteins in additional barley genotypes

**Figure 8:**
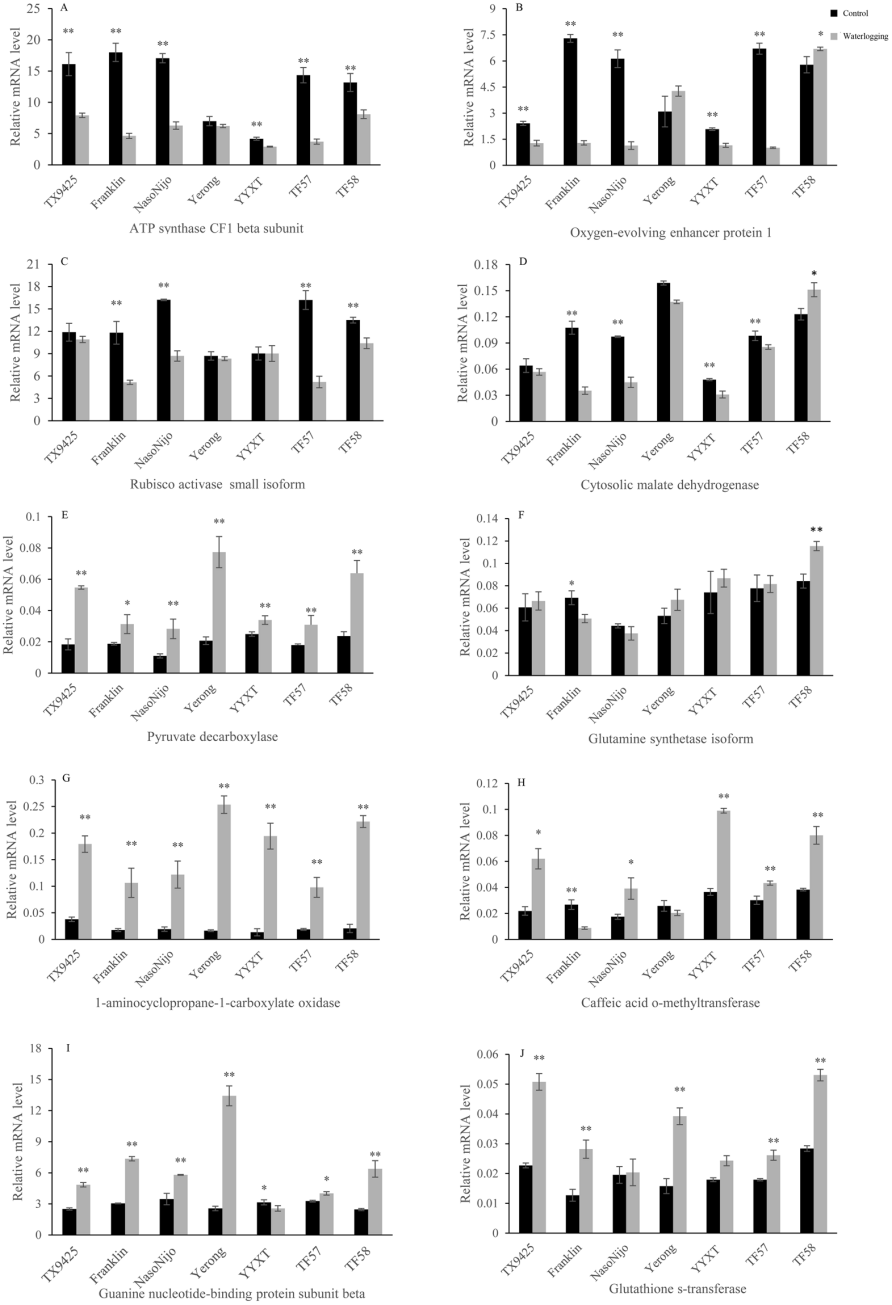
The mRNA expression of ten candidate proteins in five genotypes and two DH genotypes were assayed using quantitative real time**-**PCR in leaves (ABCD) and roots (EFGHIJ). Three biological repeats were performed for each sample, and *actin* (gi|24496452) was used as an internal reference. Statistical analysis was performed using Student’s *t*-test. The error bars indicate the SD from three biological repeats. * And **represent significant differences at *p* < 0.05 and *p* < 0.01, respectively.

To validate the candidate proteins, the mRNA expression levels of ten genes were investigated using additional genotypes with known waterlogging tolerance (Fig. 8). The expression patterns of the ATP synthase CF1 beta subunit and the Rubisco activase small isoform in the leaves were consistent in all genotypes. The expression levels significantly decreased or remained unchanged under waterlogging, with the changes being significantly greater in the sensitive genotypes (TF57, Franklin and Naso Nijo), than in tolerant genotypes. The expression levels of oxygen-evolving enhancer protein and cytosolic malate dehydrogenase were up-regulated in TF58 but down-regulated or remained unchanged in other genotypes.

The transcriptional levels of *PDC*, *ACO* and *GST* increased in the roots of all genotypes in response to waterlogging stress. The three genes had higher expression levels in the tolerant genotypes TX9425, Yerong and TF58 than in the sensitive genotypes (Fig. 8). *GS* was up-regulated only in TF58, down-regulated in Franklin, and remained unchanged in the other genotypes. Guanine nucleotide**-**binding protein subunit beta was up-regulated in all genotypes except YYXT, with the highest expression detected in Franklin, Yerong and TF58 under waterlogging stress. The expression of caffeic acid o**-**methyltransferase (*COMT*) was significantly up-regulated in TX9425, NasoNijo, YYXT, TF57 and TF58, down-regulated in Franklin and remained unchanged in Yerong. Therefore, the three genes (*PDC*, *ACO* and *GST*) are likely involved in waterlogging tolerance.

### Candidate gene confirmation by comparison with genomic information

In recent years, a large number of QTLs for waterlogging tolerance in barley were identified^6,19,20^. To validate the potential candidate genes, the present results and those of a previous genetic analysis were integrated for waterlogging tolerance. The results revealed that two candidate proteins, GST and ACO, were located in the confidence intervals of the QTL for waterlogging tolerance. *GST* was mapped to chromosome 4, positioned at 99.1 cM, which is a major QTL for aerenchyma formation and waterlogging tolerance in barley^19,20^. *ACO* was mapped to a waterlogging stress response QTL whose linkage marker was Ebmac0755 at 131.3 cM on chromosome 7^20,21^. The mRNA levels of both genes increased in the roots of different genotypes under waterlogging stress and showed higher expression in the tolerant genotypes (TF58, TX9425 and Yerong).

## Discussion

### Phenotypic alterations in waterlogged TF57 and TF58

Gas diffusion velocity significantly decreases under saturated soil conditions^22^. Waterlogging significantly inhibits plant growth and development. After 21 days of waterlogging treatment, the longest adventitious root length and the dry weight in both shoots and roots decreased between 20 and 80% compared to the control^3,6^. In this study, the growth of TF57 was severely affected after 21 days of waterlogging treatment in comparison with TF58. The SPAD values in TF57 significantly decreased under waterlogging stress, and wilted/yellowed shoots were observed (Figs 1 and 2). It has been reported that tolerant maize genotypes displayed higher chlorophyll content and lower degrees of leaf injury under waterlogging stress^23^. TF58 showed a lower degree of leaf injury and a higher shoot fresh weight and shoot dry weight, indicating that TF58 was more tolerant to waterlogging than TF57.

### Photosynthesis-related proteins

Photosynthesis is essential for metabolic synthesis and maintaining plant growth; however, this process is sensitive to abiotic stress^16^. In the present study, significant changes were observed in key photosynthetic proteins, including the Rubisco small and large subunits (L342), Rubisco activase (L252, L263 and L294), and oxygen-evolving enhancer protein (L176) (Fig. S1). Rubisco activase is a molecular chaperone that controls the switching of Rubisco from the inactive to active conformation and is a key enzyme that affects the photosynthetic rate^24^. Immunoblotting analysis suggested that the Rubisco small subunit of TF58 slightly changed due to waterlogging, while the change was more pronounced in TF57 (Fig. 7). Therefore, the photosynthetic function in barley leaves were impaired under waterlogging stress, with more severe damage observed in the sensitive genotypes. Augmented levels of MDA and O_2_^.-^ content in the leaves and roots of TF57 under waterlogging may also support this idea (Fig. 3).

### Energy- and metabolism-related proteins

Energy deprivation is one of the major factors affecting waterlogged plant survival. This is due to aerobic respiration being replaced by anaerobic respiration, and the yield of ATP significantly decreasing under waterlogging stress^25^. Enzymes related to glycolytic and fermentative pathways have been confirmed to be induced by the waterlogging treatment^12^. PDC, which catalyses the first step in the ethanolic fermentation pathway, plays key roles in the response to hypoxia and anoxia^18^ and was up-regulated in both the root and leaf tissues of cotton under waterlogging stress^8^. In this study, more PDC was induced in tolerant barley genotypes than that in sensitive genotypes under waterlogging stress (Fig. S2 and Table S3).

The ATP synthase subunits exist in chloroplasts, integrate into the thylakoid membrane, and serve as the main enzymes of the ATP biosynthetic pathway and photosynthesis^26^. In this study, the expression of the ATP synthase CF1 beta subunit was down-regulated in all genotypes, and greater changes were observed in the sensitive genotype (Fig. 7 and Table S2). These results are different from those reported in maize and cucumber, in which ATP synthase-related proteins showed high expression in tolerant plants under waterlogging stress^12,27^. These findings suggested that tolerant barley could economize energy consumption to survive under waterlogging stress.

### Ethylene and ROS production in response to waterlogging

Ethylene plays an important role in modifying plant responses to oxygen deficiency and inducing aerenchyma and adventitious root primordia formation as a signal transducer. The gaseous phytohormone ethylene is synthesized by the activation of 1-aminocyclopropane-1-carboxylic acid (ACC) synthase and ACC oxidase (ACO)^28^. In this study, ACO (AR852, NR412 and SR160) was significantly up-regulated during waterlogging in both genotypes, whereas TF58 had a higher abundance than TF57 in the adventitious roots, nodal roots and seminal roots (Table S3,S4,S5). The expression levels of *ACO* increased in the different genotypes during waterlogging (Fig. 8). Under waterlogging stress, enhanced ethylene production in leaves and roots was also observed in tolerant genotype TF58 (Fig. 3). This is similar to results of a study conducted on maize undergoing waterlogging^12^.

Reactive oxygen species (ROS), which are produced in plants experiencing various stresses, can damage the normal functions in plant cells^27^. To counteract the harmful effects of ROS, ROS scavengers are induced under abiotic stress. These scavengers include SOD, POD, ascorbate peroxidase (APX) and GST^26,29^. GST represents a major group of detoxification enzymes, which can catalyse glutathione-dependent detoxification reactions and protect plants from impairment caused by abiotic stress^30^. Previous studies have revealed that the overexpression of *GST* significantly enhanced tolerance to abiotic stress in tobacco^31^. Our results showed that the expression levels of GST were increased in all genotypes under waterlogging stress, but greater changes were observed in TX9425, Yerong and TF58, suggesting that more efficient ROS detoxification occurs in tolerant genotypes under waterlogging stress (Fig. 8). These results were further confirmed by the increased antioxidant enzyme activities (SOD, CAT and POD) in the leaves and roots of TF58 under waterlogging stress (Fig. 3).

### Cell wall biosynthesis and loosening of related proteins

The formation of adventitious roots is accompanied by cell wall biosynthesis and loosening^27^. Two cell wall biosynthesis and loosening-related proteins, caffeic acid o**-**methyltransferase (COMT, AR370) and beta**-**1,3**-**glucanase 2a (NR275 and NR287), were induced by waterlogging stress in TF58. COMT, which catalyses the methylation of caffeic acid and 5**-**hydroxyferulic acid, is an important methylating enzyme involved in lignin biosynthesis^32^. It is highly expressed in response to abiotic stresses and promotes the formation of the mechanical barrier via lignin deposition^33^. In the present study, COMT showed higher expression levels in TX9425, YYXT and TF58 than in the other genotypes, indicating that more cell wall barriers were present under waterlogging (Fig. 8). A barrier to radial O_2_ loss (ROL) in roots enhances longitudinal O_2_ diffusion by preventing loss to the surrounding anoxic soil^30^.

### A possible waterlogging stress-responsive protein network

**Figure 9:**
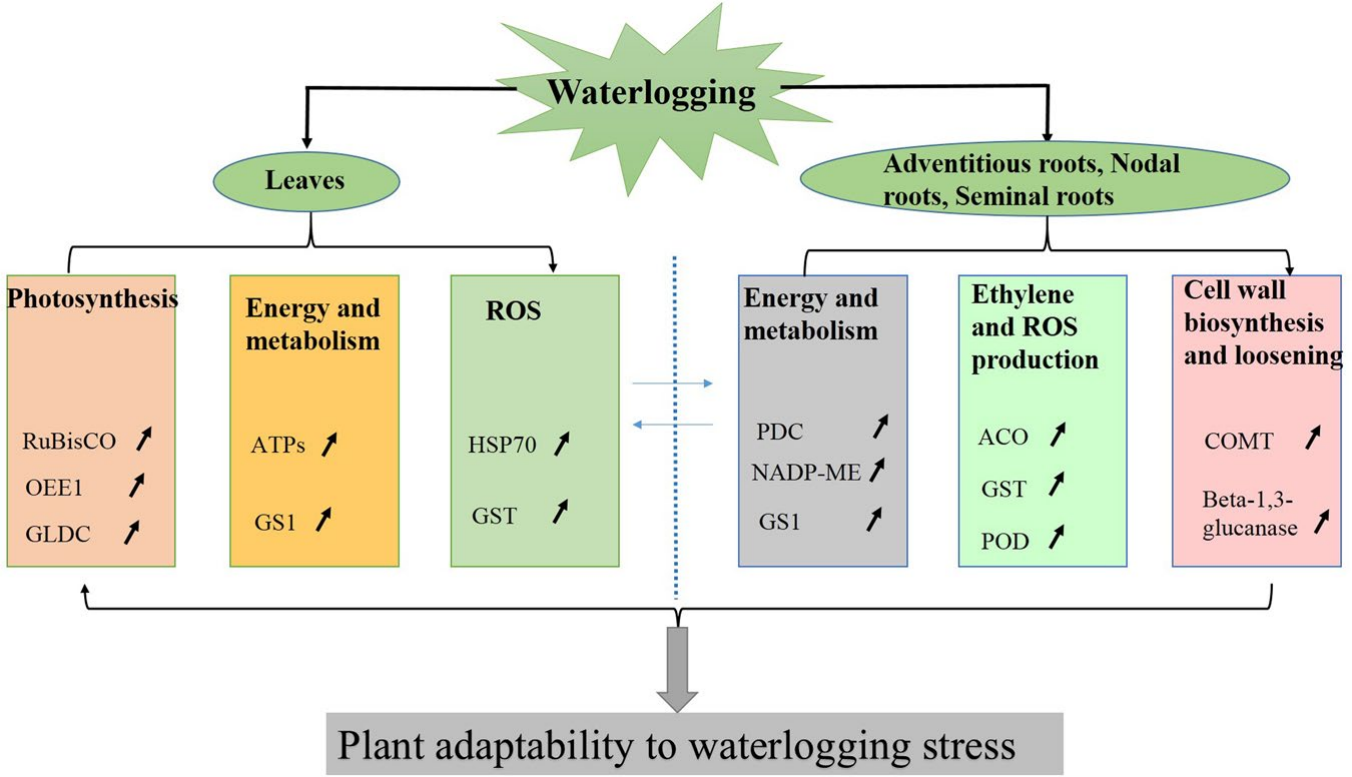
Ahypothetically integrated schematic diagram of the mechanism involved in waterlogging tolerance in TF58. An arrow (↗) indicates increased abundance. RuBisCO, Ribulose-bisphosphate carboxylase oxygenase; OEE1, oxygen-evolving enhancer protein; GLDC, glycine dehydrogenase; ATPs, ATP synthase; GS1, glutamine synthetase; HSP70, Heat shock protein 70; GST, glutathione S**-**transferases; PDC, pyruvate decarboxylase; NADP**-**ME, NADP**-**dependent malic enzyme; ACO, 1**-**amino cyclopropane 1**-**carboxylic acid oxidase; POD, peroxidase; COMT, caffeic acid o**-**methyltransferase, Beta-1,3-glucanase.

In the present study, a waterlogging responsive protein network was proposed based on the proteomic analysis (Fig. 9). Barley roots initially perceive waterlogging stress signals and accumulate ethylene by increasing the expression of ACO. Ethylene can trigger ROS production, which leads to a redox imbalance in plant cells. The synthesis of antioxidant enzymes (GST and POD) increased to reduce oxidative damage. Ethylene and ROS can induce epidermal and cortical programmed cell death, leading to the formation of adventitious roots and aerenchyma by up-regulated COMT and beta-1,3-glucanase. The energy supply increases in the roots and leaves to mitigate waterlogging stress by up-regulating PDC, NADP-ME, GS1 and ATP. Impaired photosynthesis is significantly reduced by the up-regulation of OEE1 and glycine dehydrogenase (GLDC) in the leaves. The proposition was validated by the determination of related physiological indexes (Fig. 3).

The waterlogging-tolerant genotypes possess the ability to produce higher levels of ethylene, scavenge more ROS, generate a more efficient energy supply and undergo more photosynthesis than waterlogging-sensitive genotypes under waterlogging stress. The mRNA levels of PDC, ACO and GST increased in the tolerant genotypes, and the associated proteins are important candidate biomarkers for improving waterlogging tolerance in barley. Further investigations and functional analyses of these candidate genes are required in future studies.

## Material and Methods

### Plant materials and treatments

Two barley genotypes (TF57 and TF58) derived from a doubled haploid (DH) population (TX9425/Franklin) were used for comparative proteomic analysis. TF57 is a waterlogging-sensitive genotype, whereas TF58 is a waterlogging-tolerant genotype^34^. Another five barley genotypes (TX9425, Yerong, YYXT, Franklin and Naso Nijo) were selected for further validation of potential candidate proteins. In previous studies, TX9425, Yerong and YYXT were tolerant to waterlogging stress, while Franklin and Naso Nijo were susceptible to waterlogging stress^21,35–37^.

Barley seeds were sown in PVC containers (70 cm × 50 cm × 60 cm) filled with a mixture of nutritional substance and vermiculite. The plants were grown under greenhouse conditions at a temperature of 22 ± 2 °C/day and 18 ± 2 °C/night. Waterlogging treatments started at the four-leaf stage and lasted for 3 weeks. The water in the waterlogged containers was maintained at 2–3 cm above the soil surface at all times by a water valve that controlled the water flow during the waterlogging period. The control plants were subject to normal irrigation (60–70% soil moisture content, which was measured by weighing).

### Growth, physiological and biochemical assays

To investigate morphological alterations, the TF57 and TF58 plants were harvested after a 21-day waterlogging treatment, shoot height, shoot fresh and dry weights were measured. In addition, the chlorophyll concentration of the first leaf was measured using a soil plant analysis development (SPAD) meter (SPAD-502Plus, Konica Minolta, Tokyo, Japan). Leaves, adventitious roots, seminal roots and nodal roots were collected, carefully washed and immediately frozen in liquid nitrogen for further analysis. The experiment was carried out with three biological replicates, two genotypes and two waterlogging treatments.

Fresh leaves and roots (0.5 g each) were rinsed thoroughly with distilled water. The crude enzymatic extracts of each genotype were prepared in 0.05 M phosphate buffer (pH 7.8) after being ground with a pestle and being milled to powder in liquid nitrogen. The homogenate was filtered through four layers of muslin cloth and centrifuged at 12 000 g for 10 min at 4 °C. The final supernatants were used for physiological and biochemical assays. The activities of superoxide dismutase (SOD), peroxidase (POD), catalase (CAT) and the content of malondialdehyde (MDA) and superoxide radical (O_2_^.−^) were measured using the corresponding assay kits (Institute of Jiancheng Bioengineering, Nanjing, China) according to the manufacturer’s instructions. Ethylene was determined according to a gas chromatograph device with a TRB-5 capillary column at 100 °C and a flame ionization detector. N_2_ was employed as the carrier gas^27^.

### Protein extraction for proteomic analysis

Fresh samples were powdered in liquid nitrogen with a mortar and pestle. The powder (0.5**–**1 g) was then quickly transferred to a 1.5 mL centrifuge tube. Total proteins were extracted from different organs using TRIZOL^®^ (Invitrogen, Grand Island, NY, USA), according to the manufacturer’s instruction. The samples were solubilized and incubated using a protein buffer described by Guo *et al*.^38^. Protein concentration was determined by a Bradford assay^39^.

### Two-dimensional gel electrophoresis and image analysis

Total proteins (500 μg) were loaded onto GE Healthcare 24 cm IPG gel strips (pH 4–7). The isoelectric focusing (IEF) of the acidic range IPG strips (pH 4–7) was carried out according to the manufacturer’s instructions of IPGPhor II (GE Healthcare, USA) at 20 °C for a total of 65 kVh^40^. Two-dimensional SDS-PAGE gels (12.5% linear gradient) were ran on an Ettan Daltsix electrophoresis system (GE Healthcare, USA). The procedure was set as 2.5 W per gel for 30 min, followed by 12 W per gel for 4–5 h. After electrophoresis, the protein gels were stained for spot detection using silver nitrate, as previously described^41^.

Gel images were analyzed using the Imagemaster 2D Platinum Software Version 7.0 (GE Healthcare). Spot detection was carried out using the software with the values of the parameters smooth, minimum area, and saliency set to 2, 15, and 8, respectively. Manual spot editing, such as spot deletion, splitting, and merging was performed. The determined relative spot intensities were subsequently used for statistical analysis^38^.

### Protein expression pattern analysis

The volume of each spot from the three biological replicate gels was normalized and quantified compared to the total spot volume. Power analysis of protein expression was performed between waterlogging and control samples in the two genotypes, and only those protein spots with fold changes more than 1.5 or less than 0.66, which were significant at *p* < 0.05, were considered as differentially expressed proteins^38^. The different patterns of expressed protein spots were grouped into two categories, namely, up-regulated and down**-**regulated proteins under waterlogging stress compared with the control.

### Identification of proteins by mass spectrometry (MS)

The spots that showed significant differences between waterlogging stress and control conditions were excised from the gels, washed and incubated with a trypsin digestion solution according to Guo *et al*.^26^. The digestion solution was spotted on a MALDI target plate (1.0 µL) twice and recrystallized with the CHCA matrix dissolved in 0.1% TFA/70% ACN (0.5 µL). Each protein spot was desalted with 0.01% TFA and completely dried. The acquisition of a peptide mass fingerprint was performed with a SCIEX MALDI TOF-TOF^TM^ 5800 Analyzer.

The MS/MS results were analyzed using ProteinPilot software (Foster City, CA, USA) and the online MASCOT program (http://www.matrixscience.com). Matches to protein sequences were searched against the NCBInr database restricted to Viridiplantae (green plants) and relevant parameters as previously described^41^. The identified proteins were classified using the MapMan ontology to facilitate a better understanding of the functions of the proteins^42^.

### Quantitative Real-time PCR (qRT-PCR)

Total RNA was isolated from different organs (leaves, adventitious roots, nodal roots and seminal roots) using the Trizol reagent, according to the manufacturers’ instructions (Invitrogen, Grand Island, NY, USA). The first cDNA was synthesized by Random Primer 6 and the M-MLV reverse transcriptase (Takara, Tokyo, Japan). Specific primers were designed using the Primer Premier 5.0 (Premier Biosoft International, Palo Alto, CA, USA). The primers were listed in Supplementary Table S1. Reaction was carried out in 20 µL containing 1 µL cDNA, 10 mM Tris**-**HCl (pH 8.5), 50 mM KCl, 2 mM MgCl_2_, 0.4 µL of DMSO, 200 mM dNTPs, 10 pmol/µL specific PCR primers, 1 U of Taq DNA polymerase and 0.5 µL of SYBR GREEN I fluorescence dye. The qRT-PCR was conducted in clear tubes using an Applied Biosystems ViiA^TM^ 7 Real-Time PCR System (Carlsbad City, CA, USA) as follows: 94 °C for 5 min, 40 cycles at 94 °C for 30 s, 58 °C for 30 s and 72 °C for 45 s, and a final extension at 72 °C for 5 min. The mRNA expression level was normalized using *Actin* as an internal control^12,26,27^. The relative expression levels of target genes were determined as 2^−△Ct^. For each sample, qRT-PCR was performed with three biological replicates. The average values of 2^−△Ct^ were used to determine differences in gene expression^41^.

### Western blotting analysis

Total proteins extracted from the leaves were separated via 10% SDS**-**PAGE. The protein content was quantified using the Bradford assay^39^. The resolved proteins were transferred to a PVDF Immobilon P^SQ^ transfer membrane (0.2 μm pore size) (Millipore, USA) using a semi**-**dry approach, and the membrane was blocked with 5% w/v non-fat milk powder in Tris-buffered saline containing 0.1% v/v Tween 20 (TTBS) for 1 h at room temperature (RT) with agitation. The blot was incubated in the primary antibody at a dilution of 1: 10 000 for 1 h at RT with agitation or overnight at 4 °C. The primary antibodies were rabbit polyclonal ATP synthase beta subunit antibody, a rabbit polyclonal Rubisco small subunit antibody and a rabbit polyclonal histone H3 antibody (Agrisera, Sweden). Histone H3 detection was used as the control. After being washed three times with TTBS, the blot was incubated in HRP-conjugated secondary antibodies (Agrisera, Sweden) diluted to 1:10 000 for 1 h at RT with agitation. After being washed, the immunoblot signals were detected using ECL (GE healthcare) and visualized on X**-**ray films (Fuji Medical X**-**ray film, FUJIFILM Corporation, Tokyo, Japan). Western blotting analysis experiments were repeated at least three times, and representative data were shown.

### Statistical analysis

For phenotypic, physiological parameter, spot intensity and gene expression analysis, student’s *t*-test was used to evaluate the significant differences among each genotype between the control and the waterlogging conditions. In all cases, *p* < 0.05 was considered statistically significant. The results were represented by the mean ± standard deviation (SD). Principal Components Analysis (PCA) was performed using the statistical tool box COVAIN^43^. This software can be accessed online at http://www.univie.ac.at/mosys/software.html.

## Electronic supplementary material

supporting information file

Dataset 1

Dataset 2

Dataset 3

Dataset 4
