# Supplementary material for: Elucidating the hypoxic stress response in barley (Hordeum vulgare L.) during waterlogging: A proteomics approach
Source: Sci Rep. 2018 Jun 25;8:9655. doi: 10.1038/s41598-018-27726-1 (PMC6018542; doi:10.1038/s41598-018-27726-1)
Supplement: Supplementary file 1 — supporting information file [file 41598_2018_27726_MOESM1_ESM.docx]

Elucidating the hypoxic stress response in barley (*Hordeum vulgare* L.) during waterlogging: A proteomics approach

Haiye Luan†^1,2^, Huiquan Shen†^2^,Yuhan Pan^1^, Baojian Guo^1^, Chao Lv^1^& Rugen Xu*^1^

(^1^ Jiangsu Key Laboratory of Crop Genetics and Physiology/Co-Innovation Center for Modern Production Technology of Grain Crops, Key Laboratory of Plant Functional Genomics of the Ministry of Education, Barley Research Institution of Yangzhou University, Yangzhou University, Yangzhou, 225009, China)

(^2^ Institute of Agricultural Science in Jiangsu Coastal Areas, Yancheng, 224002, China)

†These authors contributed equally to this work

*Correspondence: Rugen Xu

Email: [rgxu@yzu.edu.cn](mailto:rgxu@yzu.edu.cn)

Phone: 0086-0514-87979254

Address: Yangzhou University, 48 Wenhui East Road, Yangzhou, Jiangsu, 225009, China

**Supporting Information File:**

**Figure S1** 2**-**DE pattern of leaves proteins of barely under control and waterlogging conditions. TF57 under control conditions (A); TF57 under waterlogging conditions (B); TF58 under control conditions (C); TF58 under waterlogging conditions (D).

**Figure S2** 2**-**DE pattern of adventitious roots of barely under control and waterlogging conditions. TF57 under control conditions (A); TF57 under waterlogging conditions (B); TF58 under control conditions (C); TF58 under waterlogging conditions (D).

**Figure S3** 2-DE pattern of nodal roots of barely under control and waterlogging. TF57 under control conditions (A); TF57 under waterlogging conditions (B); TF58 under control conditions (C); TF58 under waterlogging conditions (D).

**Figure S4** 2-DE pattern of seminal roots of barely under control and waterlogging. TF57 under control conditions (A); TF57 under waterlogging conditions (B); TF58 under control conditions (C); TF58 under waterlogging conditions (D).

**Figure S5** Principal component analysis (PCA) considering two genotypes under two conditions (Control and Waterlogging), based on all protein spots present in three biological replicates. PCA of leaves proteins (A); PCA of adventitious roots proteins (B); PCA of nodal roots proteins (C); PCA of seminal roots proteins (D). C: Control; W: Waterlogging.

**Figure S6** The mRNA expression of four candidate proteins in TF57 and TF58 leaves were assayed using quantitative real time**-**PCR. Three biological repeats were performed for each sample, and *actin* (gi|24496452) was used as an internal reference. Statistical analysis was performed using Student's *t*-test. The error bars indicate the SD from three biological repeats. * and ** represent significant differences at *p* < 0.05 and *p* < 0.01, respectively. C: Control; W: Waterlogging.

**Figure S7** The mRNA expressions levels of six candidate proteins in TF57 and TF58 adventitious roots, nodal roots and seminal roots were assayed using quantitative real-time PCR. Three replicates were performed for each sample. Three biological repeats were performed for each sample, and *actin* (gi|24496452) was used as an internal reference. Statistical analysis was performed using Student's *t*-test. The error bars indicate the SD from three biological repeats. * and ** represent significant differences at *p* < 0.05 and *p* < 0.01, respectively. C: Control; W: Waterlogging.

**Figure S8** The original 2-DE gels for leaf, adventitious root, nodal root, seminal root of barley under control and waterlogging

**Table S1** Specific primers for quantitative real time PCR (qRT-PCR) analysis.

**Table S2** The differentially expressed proteins of leaves between TF57 and TF58 under control and waterlogging stress.

^a^ The number of spot in gels indicates differentially expressed protein as given in Fig. S2; ^b^ Names and species of the proteins obtained via the MASCOT software from the NCBInr database; ^c^ Accession number from the NCBInr database; ^d^ Theoretical molecular weight and isoelectric point; ^e^ Experimental molecular weight and isoelectric point; ^f^ Number of query matched peptides; ^g^ Sequence coverage; ^h^ Statistical probability of the predicted protein is calculated by MASCOT; ^i^ The fold change is calculated using the mean value of each spot in normalized spot volume from gels comparing under control and waterlogging conditions. ↑ and ↓ indicate specifically expressed protein under control and waterlogging conditions, respectively.

**Table S3** The differentially expressed proteins of adventitious roots between TF57 and TF58 under control and waterlogging stress.

^a^ The number of spot in gels indicates differentially expressed protein as given in Fig. S3; ^b^ Names and species of the proteins obtained via the MASCOT software from the NCBInr database; ^c^ Accession number from the NCBInr database; ^d^ Theoretical molecular weight and isoelectric point; ^e^ Experimental molecular weight and isoelectric point; ^f^ Number of query matched peptides; ^g^ Sequence coverage; ^h^ Statistical probability of the predicted protein is calculated by MASCOT; ^i^ The fold change is calculated using the mean value of each spot in normalized spot volume from gels comparing under control and waterlogging conditions. ↑ and ↓ indicate specifically expressed protein under control and waterlogging conditions, respectively.

**Table S4** The differentially expressed proteins of nodal roots between TF57 and TF58 under control and waterlogging stress.

^a^ The number of spot in gels indicates differentially expressed protein as given in Fig. S4; ^b^ Names and species of the proteins obtained via the MASCOT software from the NCBInr database; ^c^ Accession number from the NCBInr database; ^d^ Theoretical molecular weight and isoelectric point; ^e^ Experimental molecular weight and isoelectric point; ^f^ Number of query matched peptides; ^g^ Sequence coverage; ^h^ Statistical probability of the predicted protein is calculated by MASCOT; ^i^ The fold change is calculated using the mean value of each spot in normalized spot volume from gels comparing under control and waterlogging conditions. ↑ and ↓ indicate specifically expressed protein under control and waterlogging conditions, respectively.

**Table S5** The differentially expressed proteins of seminal roots between TF57 and TF58 under control and waterlogging stress.

^a^ The number of spot in gels indicates differentially expressed protein as given in Fig. S5; ^b^ Names and species of the proteins obtained via the MASCOT software from the NCBInr database; ^c^ Accession number from the NCBInr database; ^d^ Theoretical molecular weight and isoelectric point; ^e^ Experimental molecular weight and isoelectric point; ^f^ Number of query matched peptides; ^g^ Sequence coverage; ^h^ Statistical probability of the predicted protein is calculated by MASCOT; ^i^ The fold change is calculated using the mean value of each spot in normalized spot volume from gels comparing under control and waterlogging conditions. ↑ and ↓ indicate specifically expressed protein under control and waterlogging conditions, respectively.

**Figure S1** 2**-**DE pattern of leaves proteins of barely under control and waterlogging conditions. TF57 under control conditions (A); TF57 under waterlogging conditions (B); TF58 under control conditions (C); TF58 under waterlogging conditions (D).


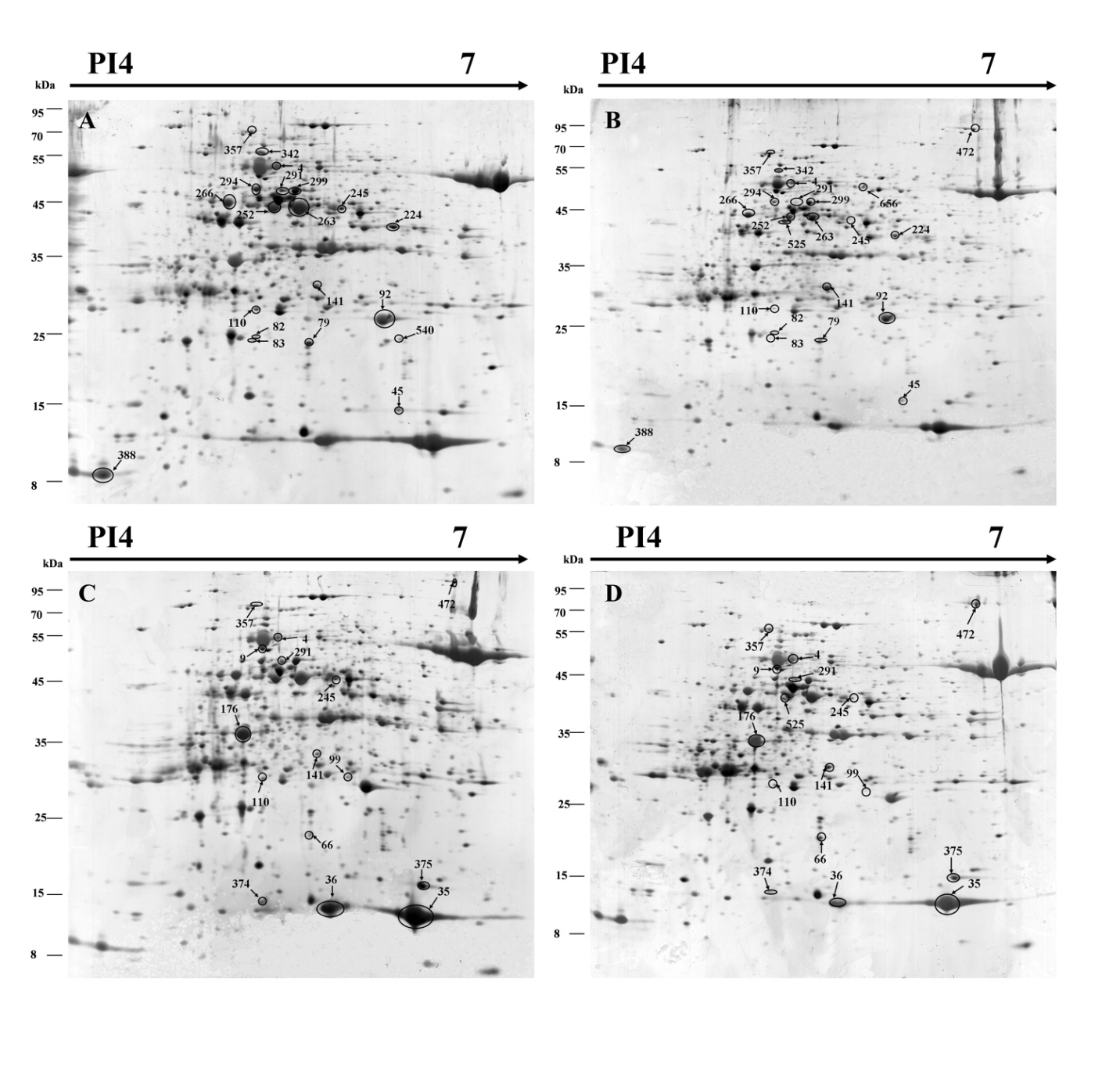


**Figure S2** 2**-**DE pattern of adventitious roots of barely under control and waterlogging conditions. TF57 under control conditions (A); TF57 under waterlogging conditions (B); TF58 under control conditions (C); TF58 under waterlogging conditions (D).


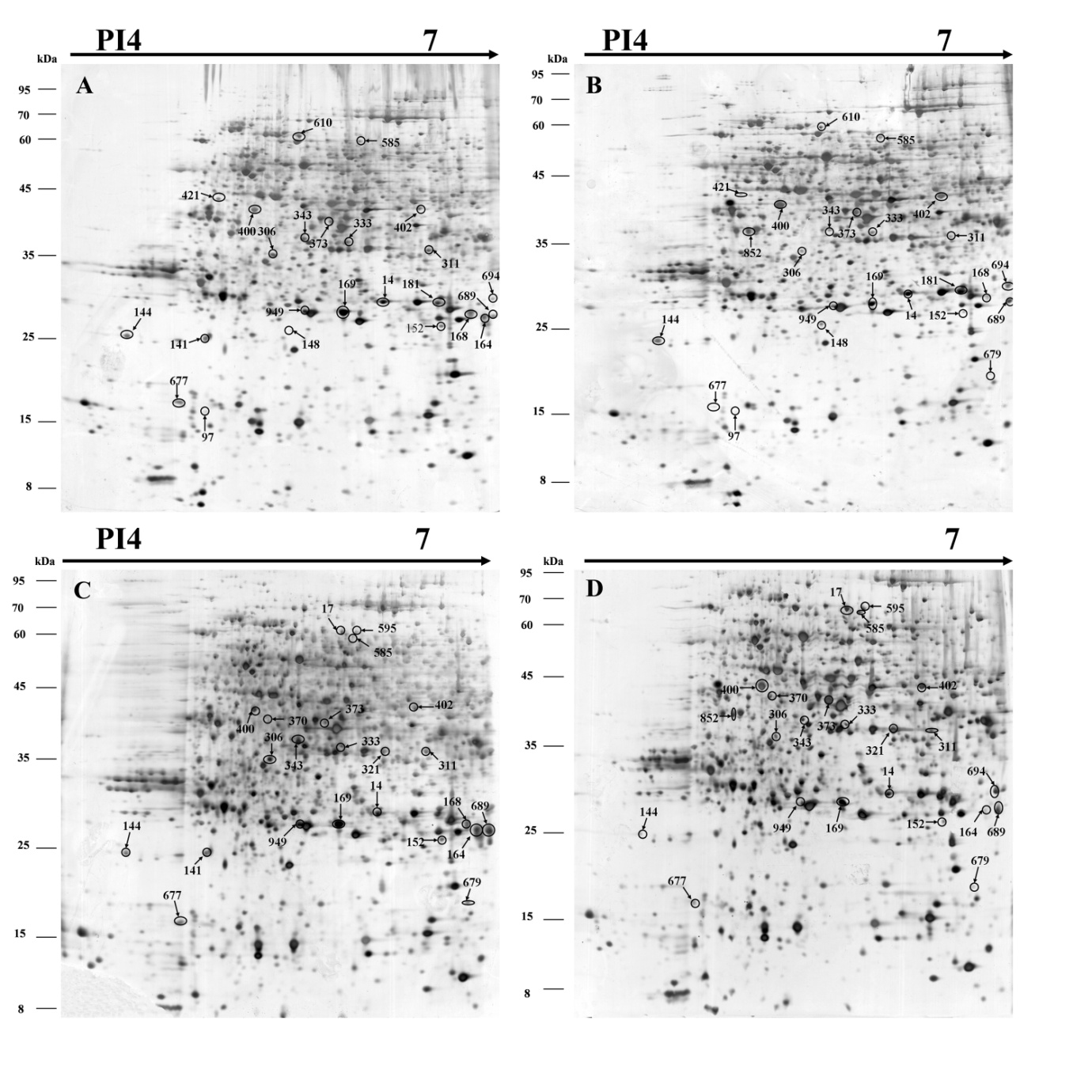


**Figure S3** 2-DE pattern of nodal roots of barely under control and waterlogging. TF57 under control conditions (A); TF57 under waterlogging conditions (B); TF58 under control conditions (C); TF58 under waterlogging conditions (D).


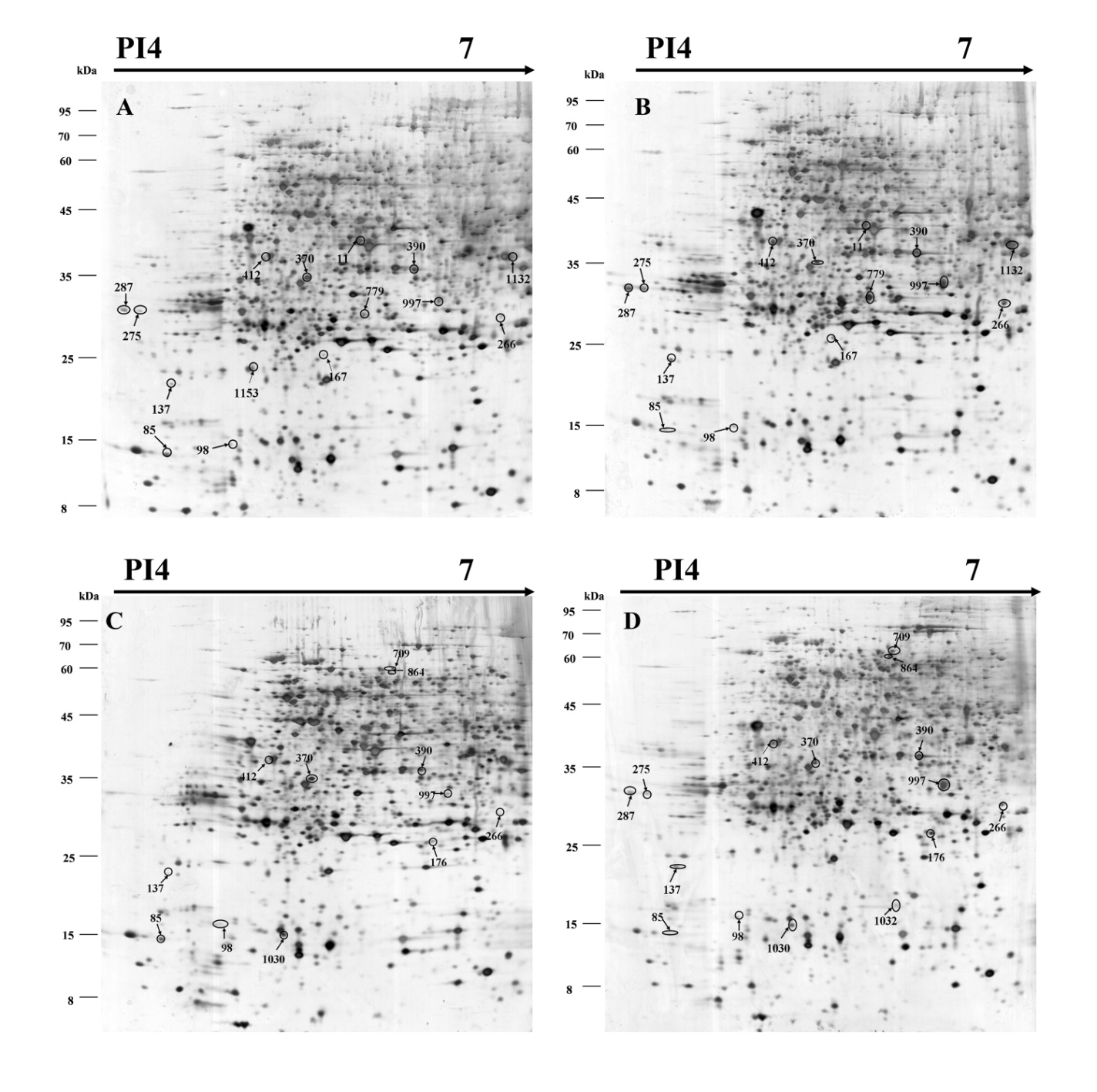


**Figure S4** 2-DE pattern of seminal roots of barely under control and waterlogging. TF57 under control conditions (A); TF57 under waterlogging conditions (B); TF58 under control conditions (C); TF58 under waterlogging conditions (D).


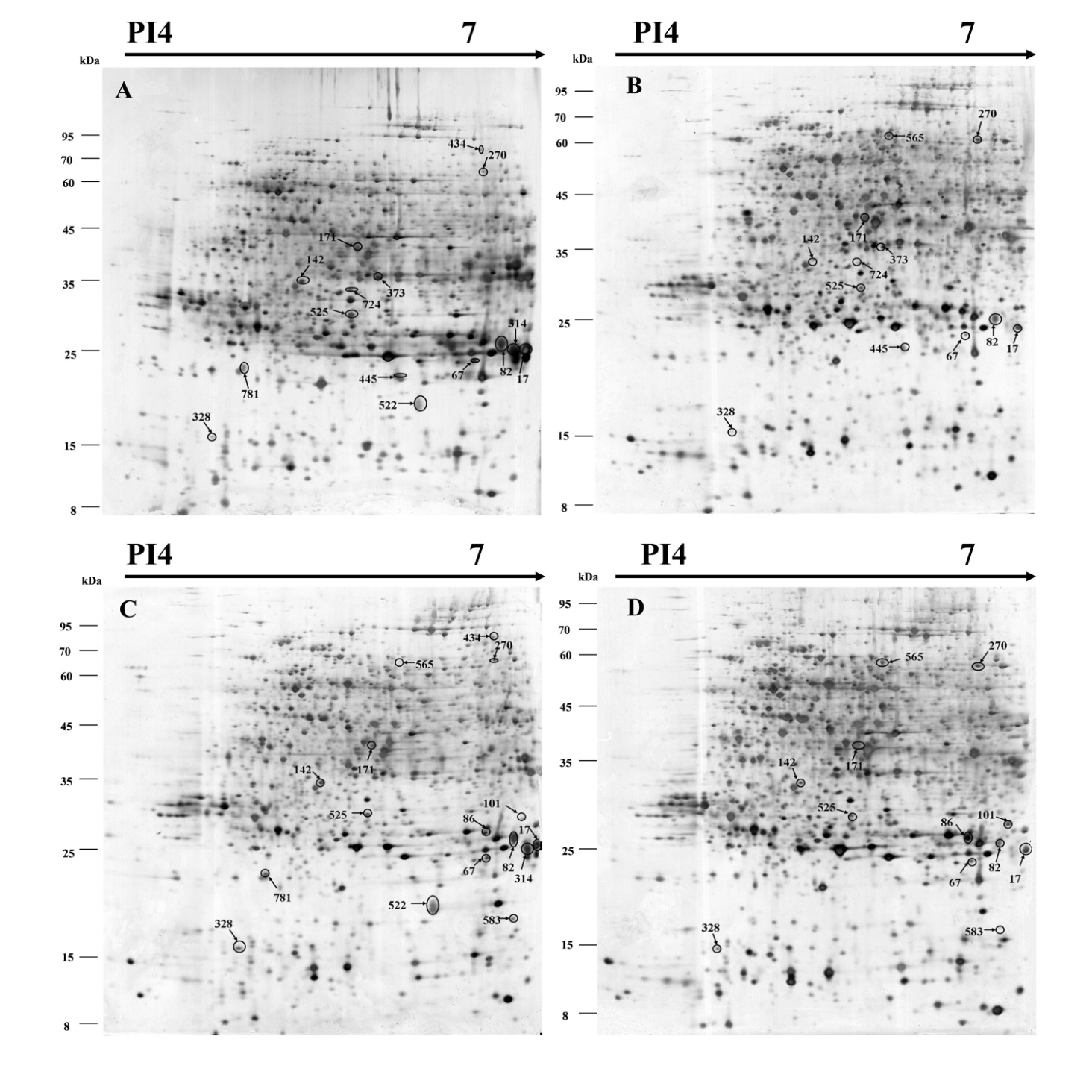


**Figure S5** Principal component analysis (PCA) considering two genotypes under two conditions (Control and Waterlogging), based on all protein spots present in three biological replicates. PCA of leaves proteins (A); PCA of adventitious roots proteins (B); PCA of nodal roots proteins (C); PCA of seminal roots proteins (D). C: Control; W: Waterlogging.


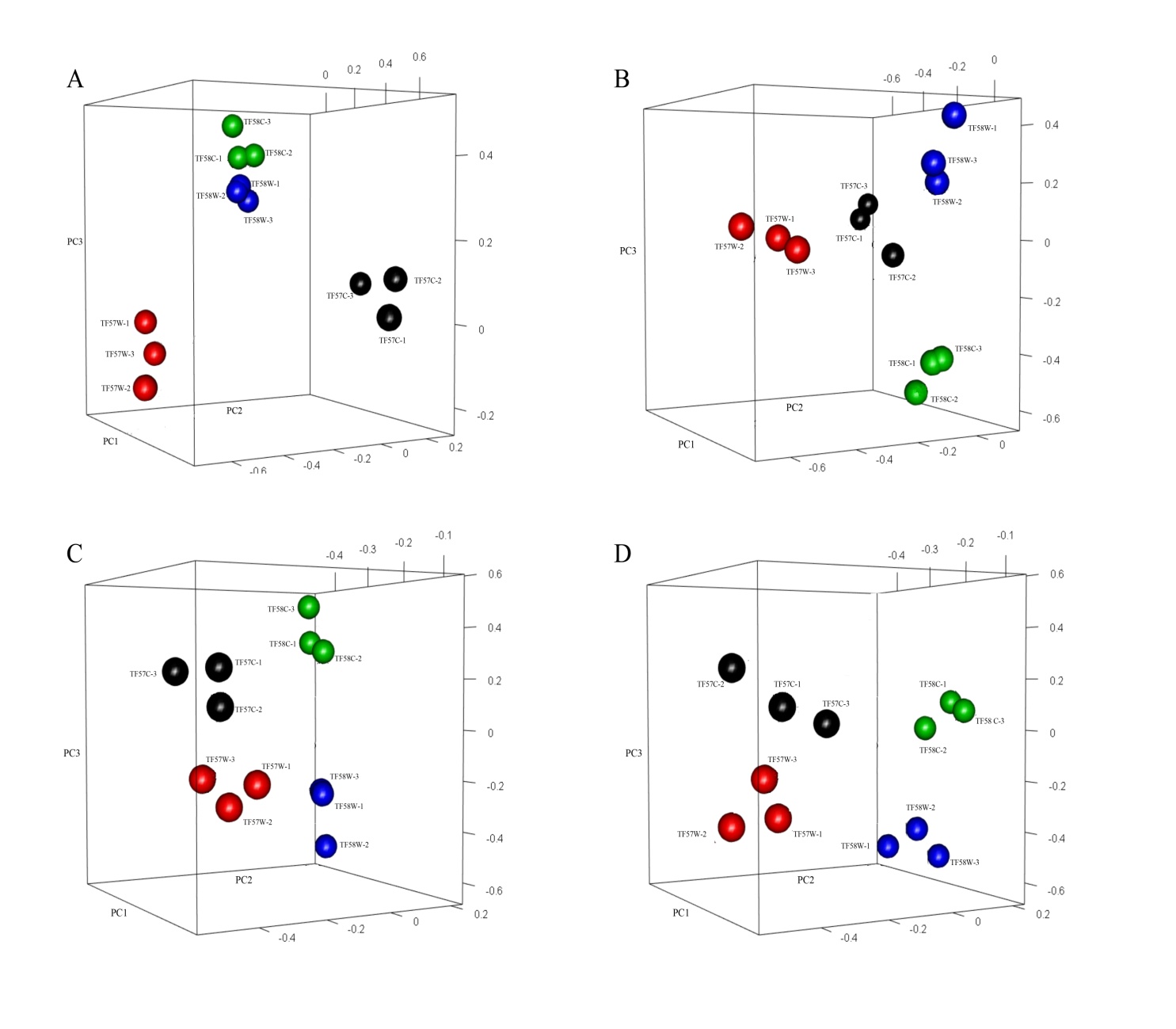


**Figure S6** The mRNA expression of four candidate proteins in TF57 and TF58 leaves were assayed using quantitative real time**-**PCR. Three biological repeats were performed for each sample, and *actin* (gi|24496452) was used as an internal reference. Statistical analysis was performed using Student's *t*-test. The error bars indicate the SD from three biological repeats. * and ** represent significant differences at *p* < 0.05 and *p* < 0.01, respectively. C: Control; W: Waterlogging.


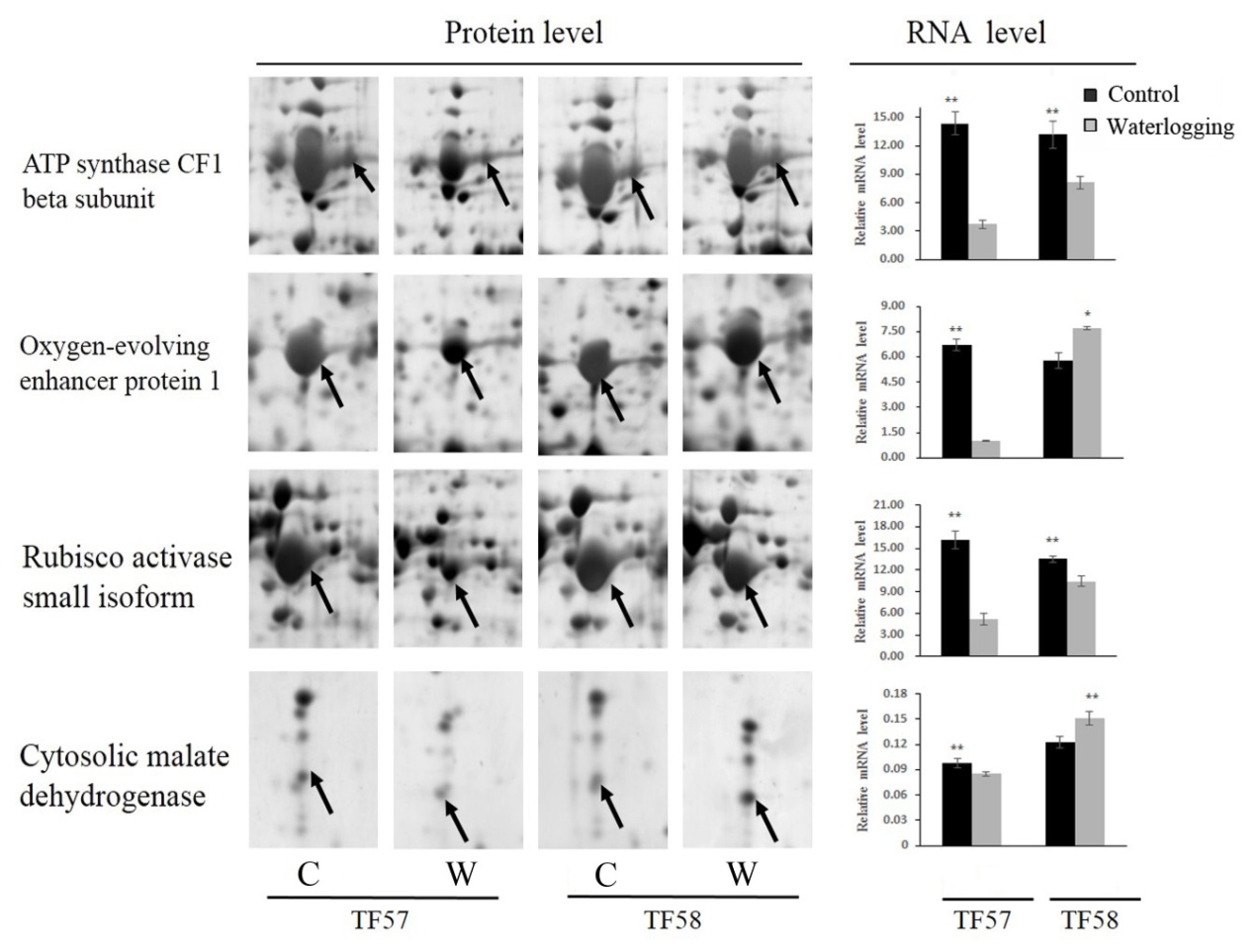


**Figure S7** The mRNA expressions levels of six candidate proteins in TF57 and TF58 adventitious roots, nodal roots and seminal roots were assayed using quantitative real-time PCR. Three replicates were performed for each sample. Three biological repeats were performed for each sample, and *actin* (gi|24496452) was used as an internal reference. Statistical analysis was performed using Student's *t*-test. The error bars indicate the SD from three biological repeats. * and ** represent significant differences at *p* < 0.05 and *p* < 0.01, respectively. C: Control; W: Waterlogging.


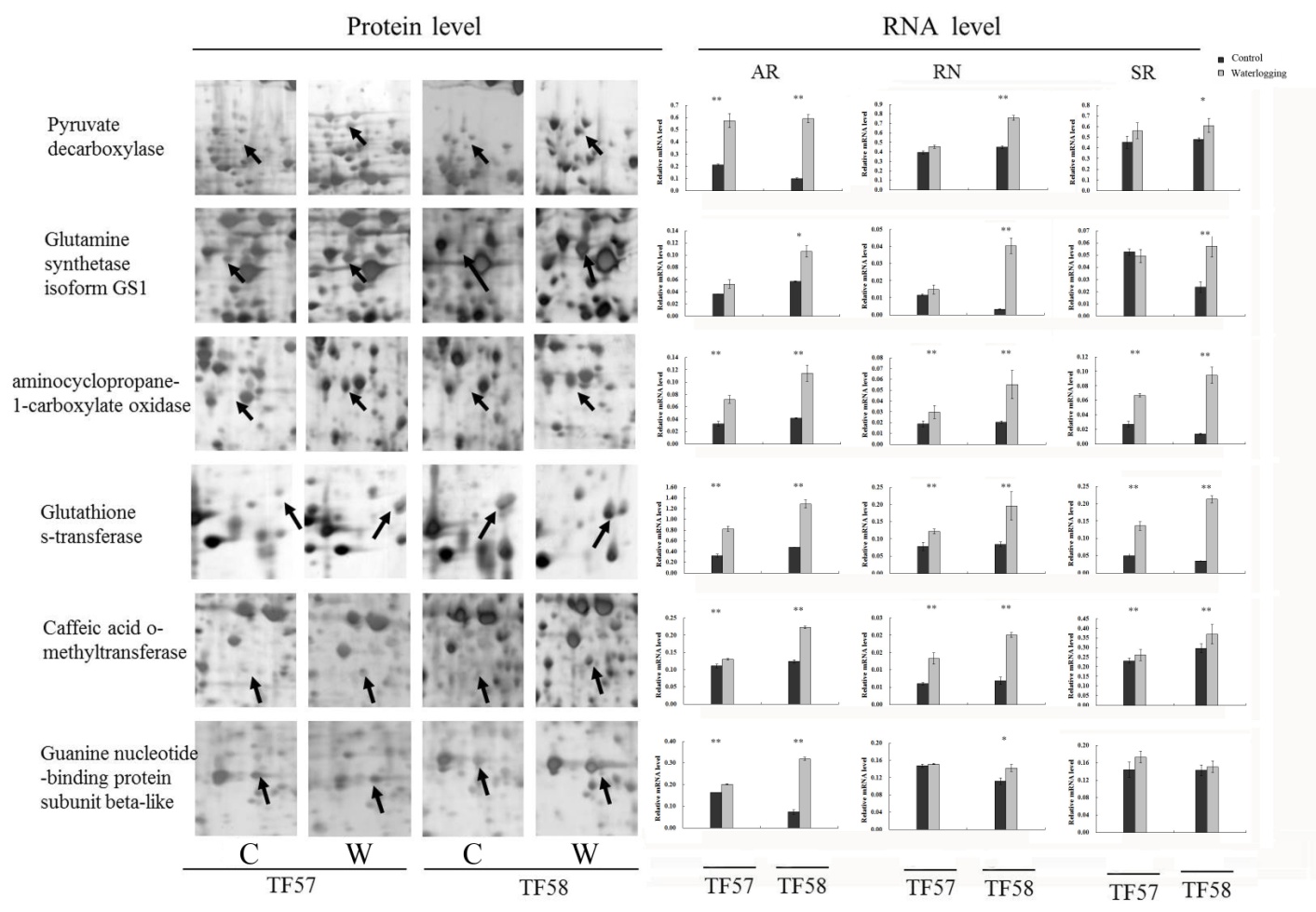


**Figure S8** The original 2-DE gels for leaf, adventitious root, nodal root, seminal root of barley under control and waterlogging

TF57 leaf under control


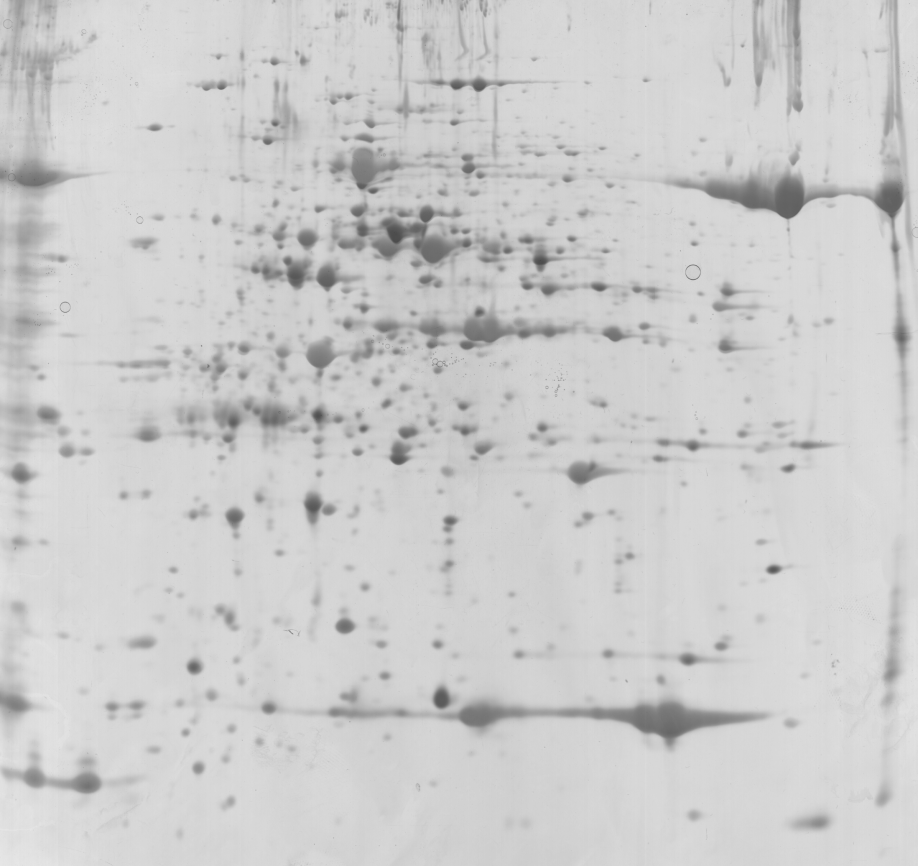


TF57 leaf under waterlogging


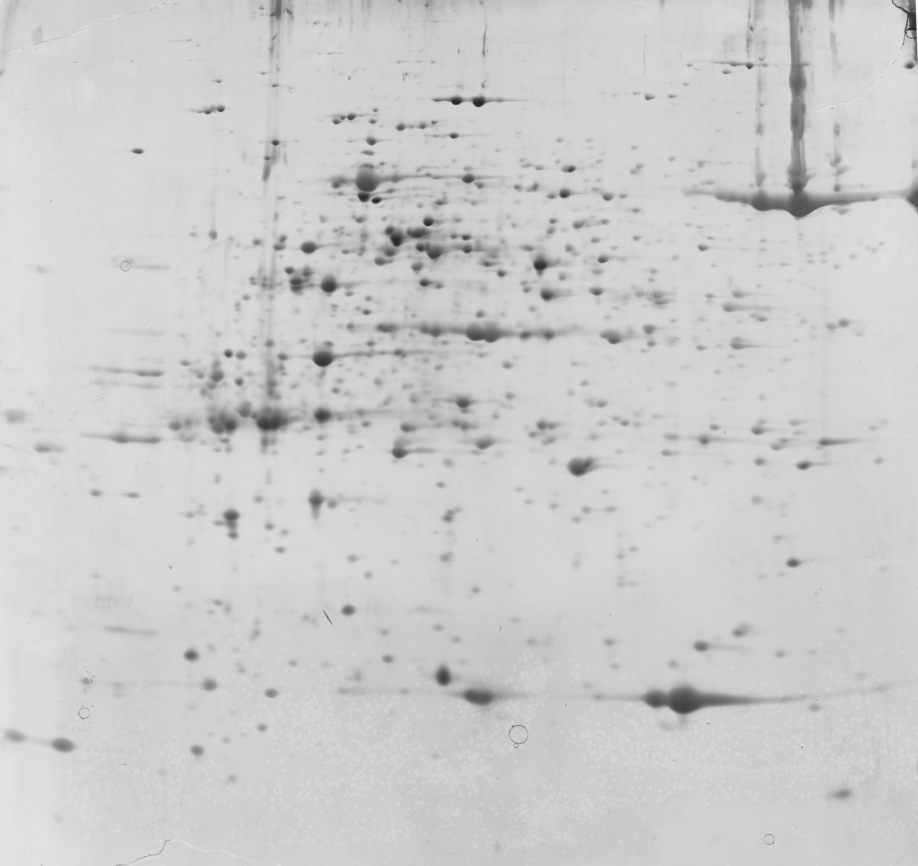


TF58 leaf under control


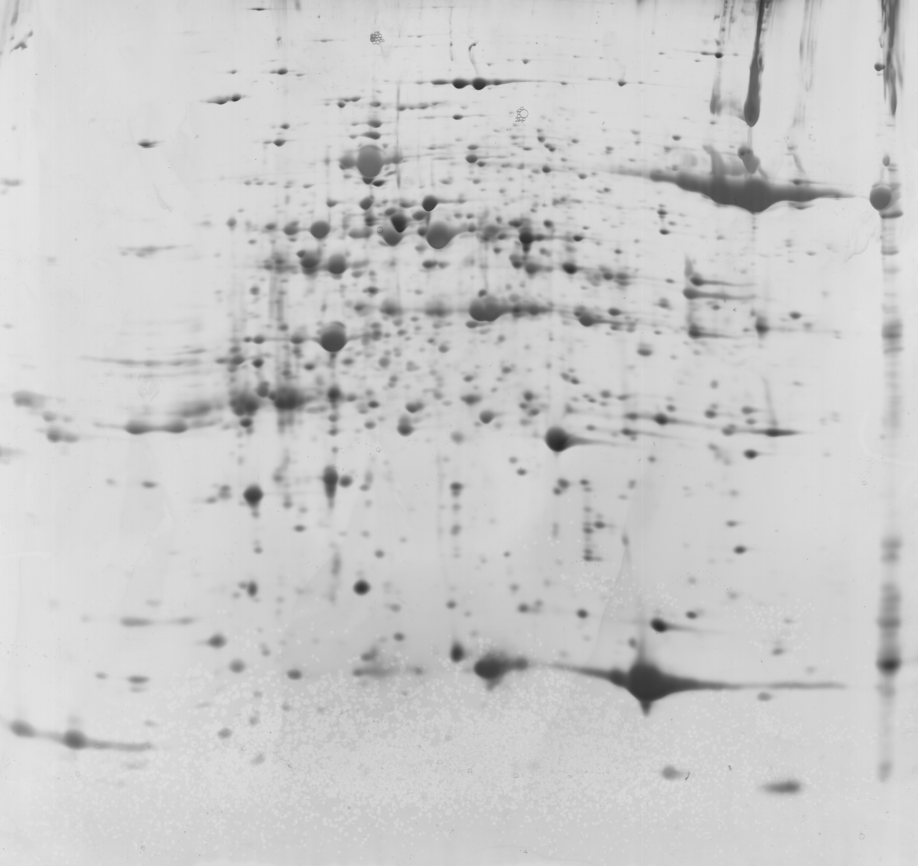


TF58 leaf under waterlogging


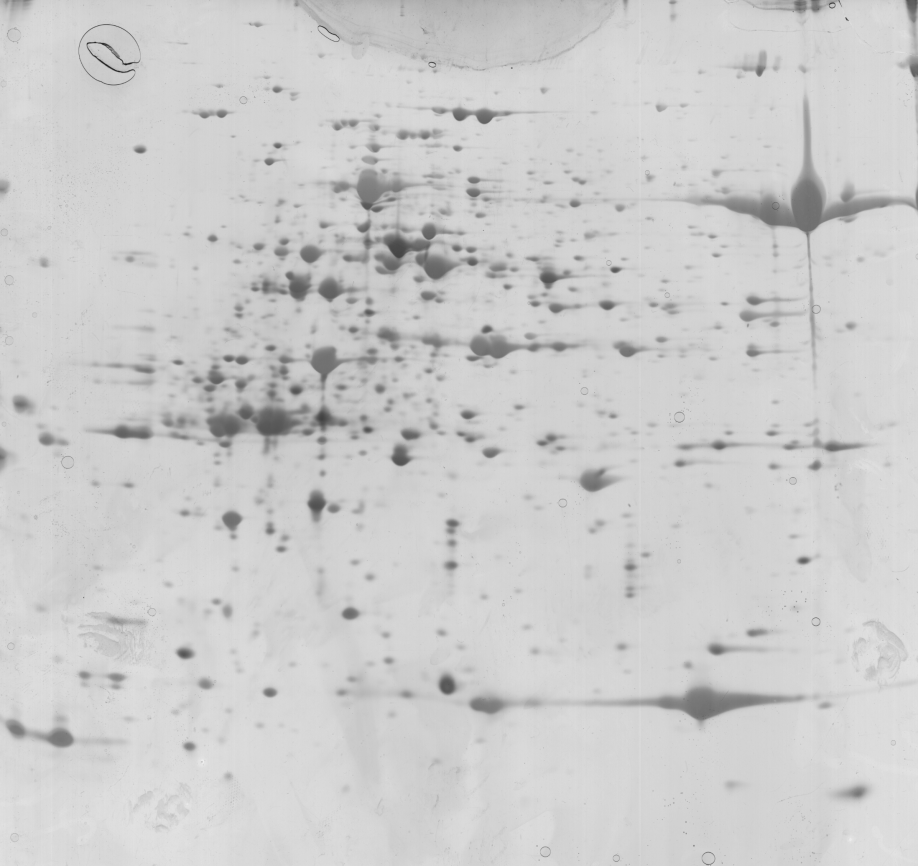


TF57 adventitious root under control





TF57 adventitious root under waterlogging





TF58 adventitious root under control





TF58 adventitious root under waterlogging





TF57 nodal root under control


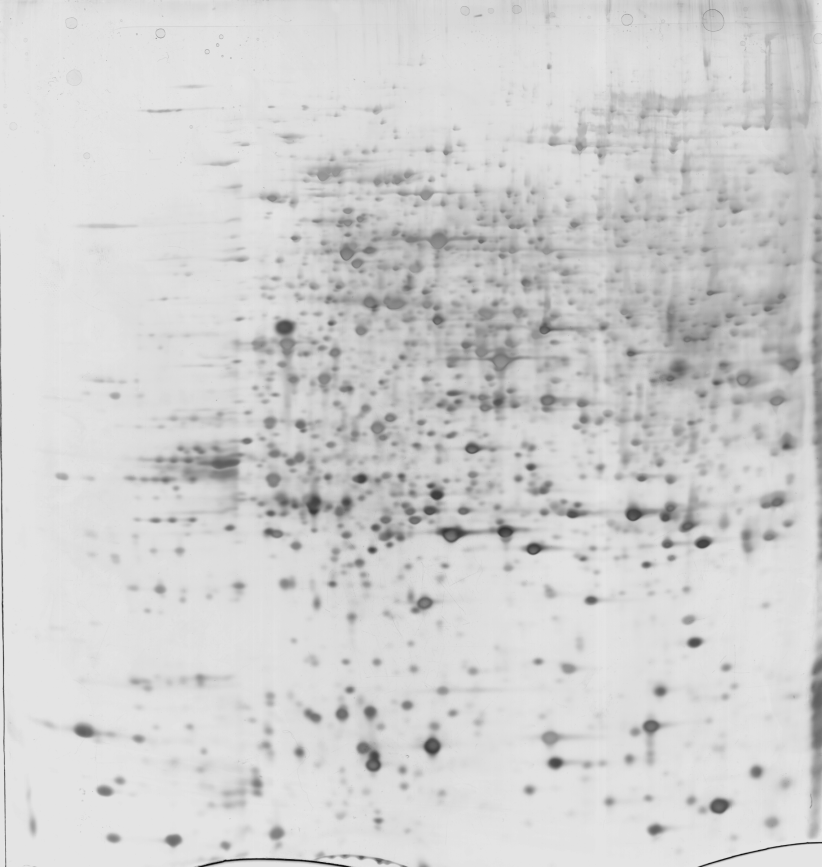


TF57 nodal root under waterlogging


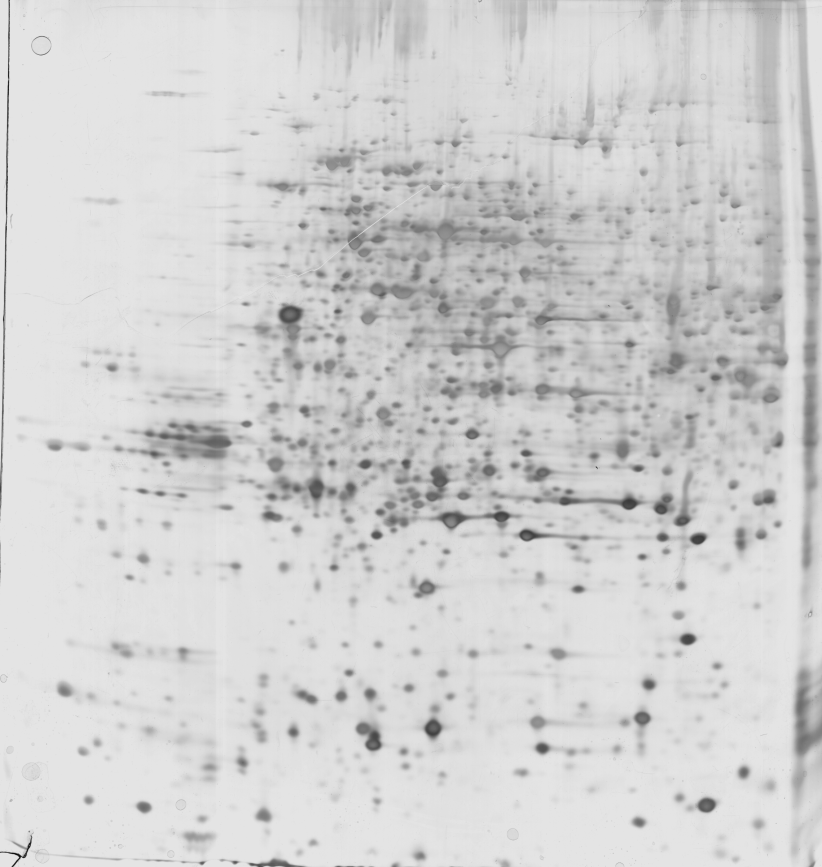


TF58 nodal root under control


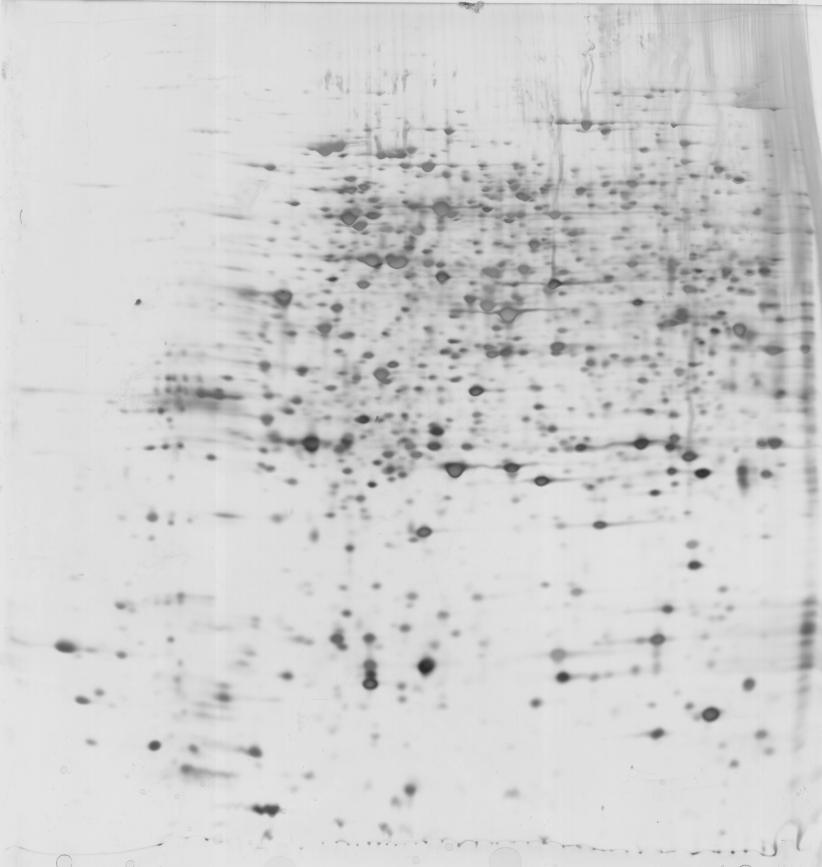


TF58 nodal root under waterlogging


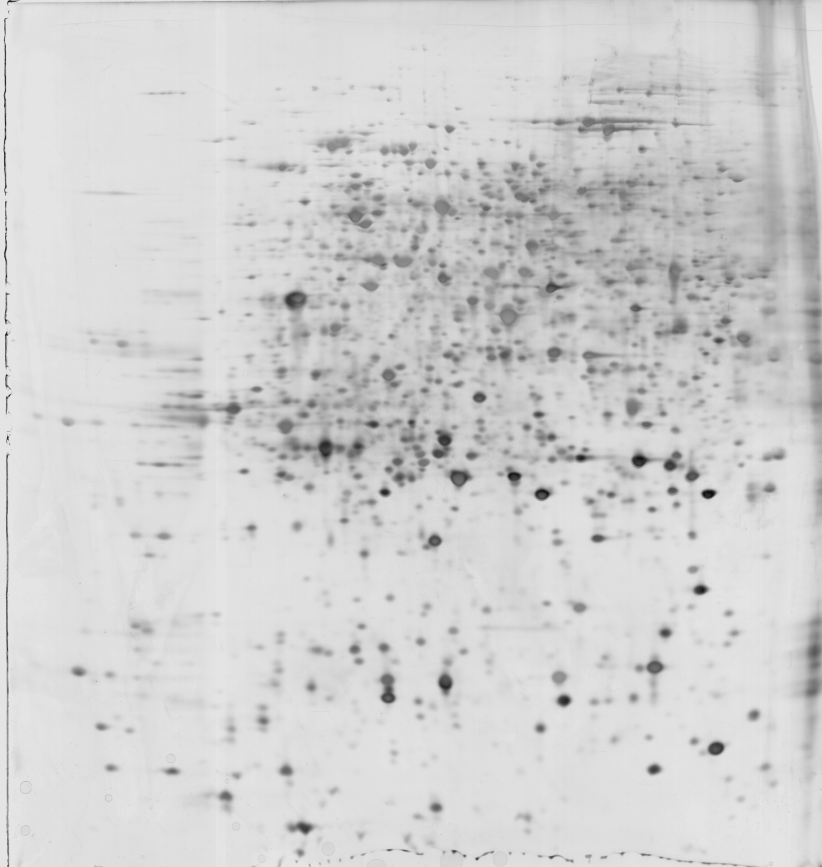


TF57 seminal root under control


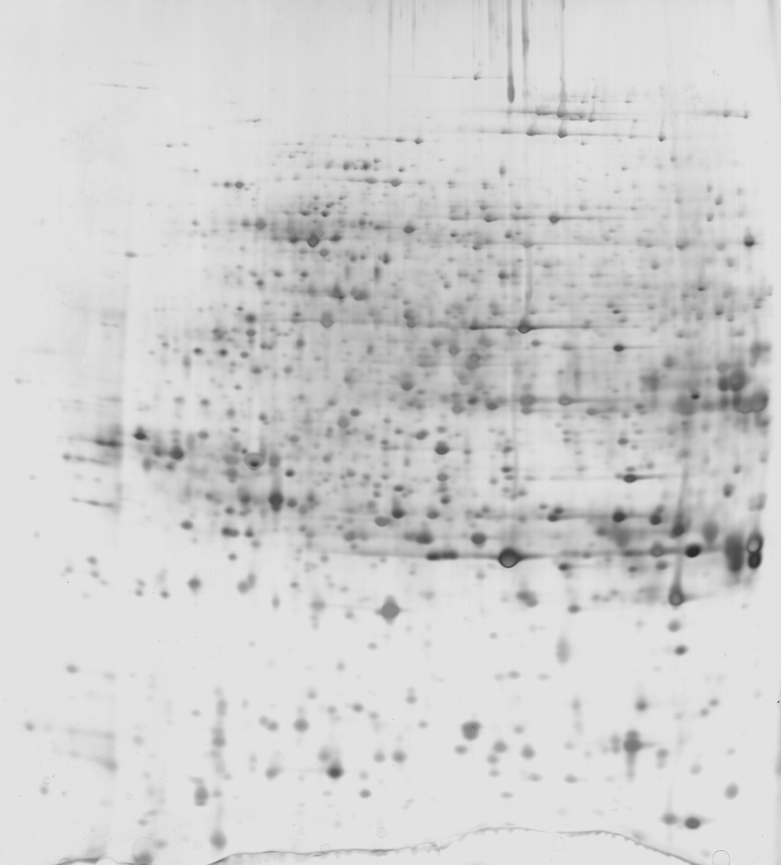


TF57 seminal root under waterlogging


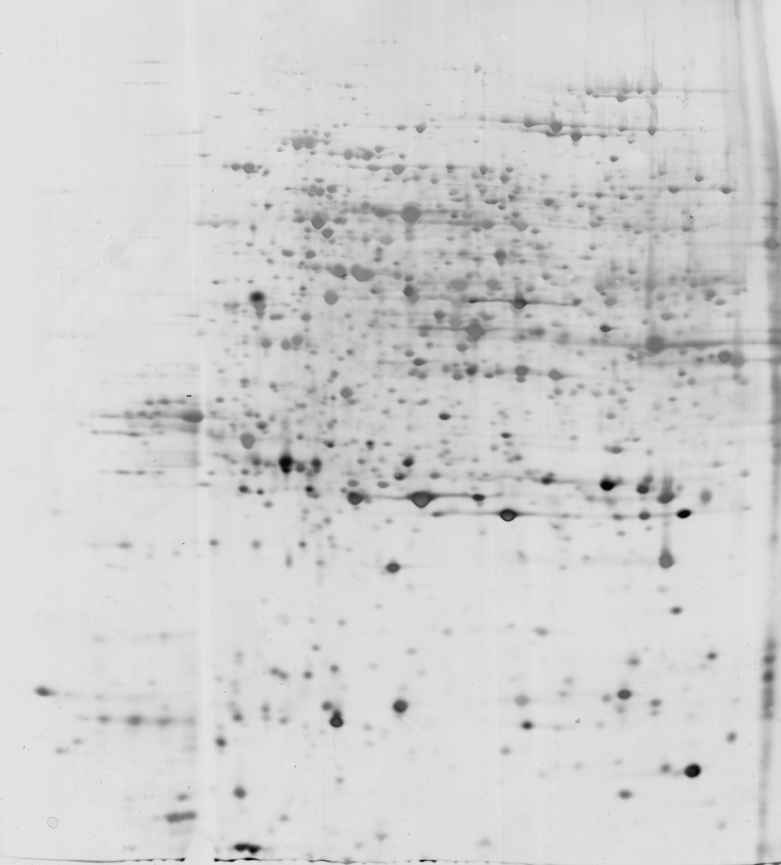


TF58 seminal root under control


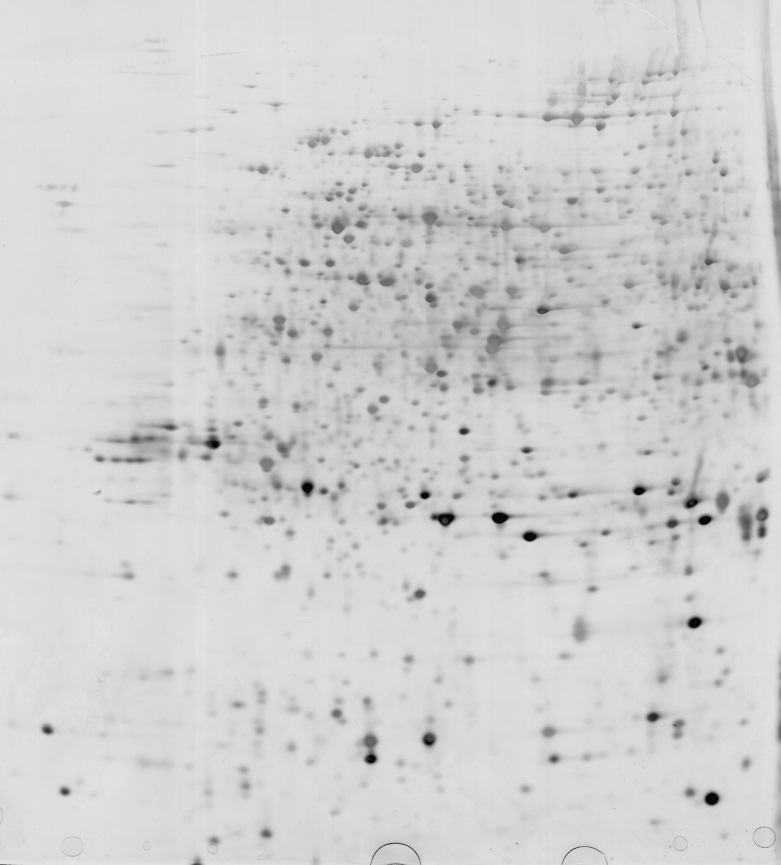


TF58 seminal root under waterlogging


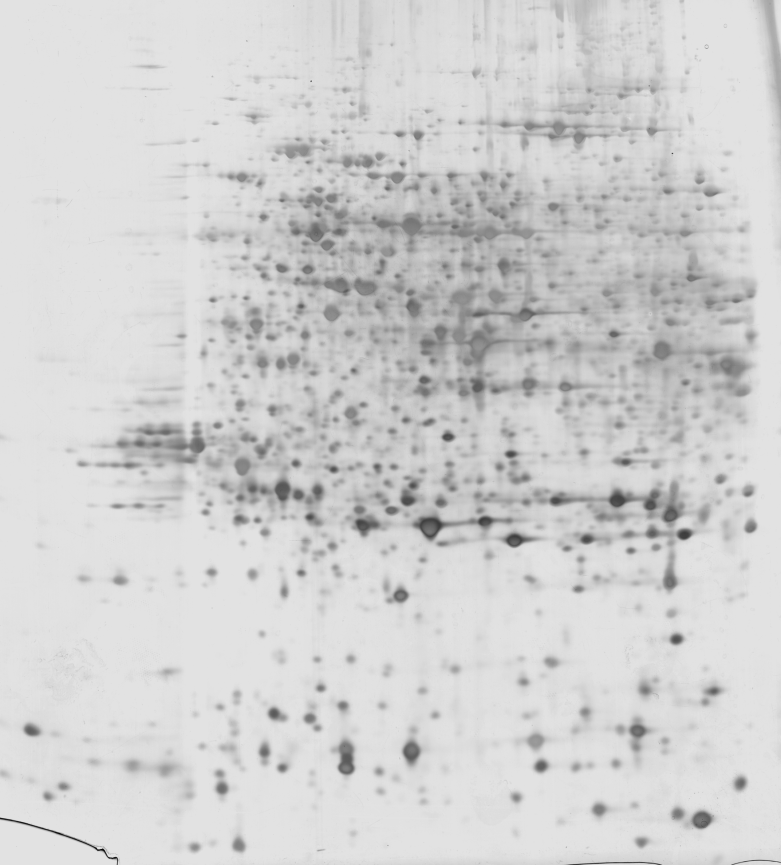


**Table S1** Specific primers for quantitative real time PCR (qRT-PCR) analysis

| Protein ID ^a)^ | Gene name | Forword primer (5'-3') | Reverse primer (5'-3') | Fragement length (bp) |
| --- | --- | --- | --- | --- |
| gi\|118430395 | ATP synthase CF1 beta subunit | GACCGGAACACTGAGAGGAG | GGACGGGTTGATGAGAGGAA | 61 |
| gi\|326494020 | oxygen-evolving enhancer protein 1 | CCCGTCTCACTTACACCCTT | CCGTCCTTCTCCTCGAACTT | 85 |
| gi\|313574196 | ribulose-1,5-bisphosphate carboxylase/oxygenase activase small isoform | ATGCCAAGAACTACGACCCA | CGATGCGGACTTAGGTAGGT | 110 |
| gi\|326531984 | cytosolic malate dehydrogenase | CCTATCCTCGCCTCCAGAAG | CCAAGCATAATTCCCCTGGC | 134 |
| gi\|326528981 | pyruvate decarboxylase | ATGCTTACGGGTGACAATGC | CTGGAATTCATACCCGCAGC | 99 |
| gi\|417356961 | glutamine synthetase isoform GS1 | CGATCTCCATTCTCTCGCCT | AACGGGTCCTTGAAAATGGC | 144 |
| gi\|397740906 | 1-aminocyclopropane-1-carboxylate oxidase 1 | AAGGACTGGGGCTTCTTCTG | CTCGTCGTAGTGGGCATACA | 93 |
| gi\|326529393 | glutathione s-transferase | TGGAAGCAGGTGGTCAAGAT | TGTGAAGCCCGATGGAAGAT | 141 |
| gi\|326505616 | caffeic acid o-methyltransferase | GGTGATCGTCGTGGAAATCG | GTCCTCTCCTTTCCACCCTC | 116 |
| gi\|326491885 | guanine nucleotide-binding protein subunit beta-like protein a-like | GGCCTCTGTTCCGGTAAAAC | CATGACTGCGGCTAAGACAC | 116 |
| gi\|24496452 | actin | GGTCCATCCTAGCCTCACTC | GATAACAGCAGTGGAGCGCT | 129 |

Note: a) Protein ID against the NCBI database;

| **Table S2** The differentially expressed proteins of leaves between TF57 and TF58 under control and waterlogging stress. | | | | | | | | | | | |
| --- | --- | --- | --- | --- | --- | --- | --- | --- | --- | --- | --- |
|  |  |  |  |  |  |  |  |  |  |  |  |
| **Spot No^a^** | **Protein Name^b^** | **Protein Id^c^** | **Theoretical Mr(kDa)/p*I*^d^** | **Experimental Mr(kDa)/p*I*^e^** | **Matched Peptides^f^** | **Cov (%)^g^** | **Score^h^** | **Species** | **Change folds^i^** | | **Peptides Squence** |
|  |  |  |  |  |  |  |  |  | **TF57** | **TF58** |  |
| **Light reaction** | | | | | | | | | | |  |
| L388 | Plastocyanin,chloroplastic | gi\|130269 | 12.75/4.59 | 12.54/4.51 | 1 | 15% | 165 | *Hordeum vulgare* | 0.39 | 1.15 | K.NNAGYPHNVVFDEDAVPSGVDVSK.I |
| L176 | Oxygen-evolving enhancer protein 1 | gi\|326494020 | 34.67/5.75 | 37.21/5.21 | 4 | 19% | 219 | *Hordeum vulgare* | 1.1 | 1.69 | K.RLTFDEIQSK.T |
|  |  |  |  |  |  |  |  |  |  |  | K.DGIDYAAVTVQLPGGER.V |
|  |  |  |  |  |  |  |  |  |  |  | K.DVKIQGVWYAQLESN + 2 Deamidated (NQ) |
|  |  |  |  |  |  |  |  |  |  |  | K.QLVATGKPESFSGPFLVPSYR.G |
| L92 | Oxygen-evolving enhancer protein chloroplastic-like | gi\|131394 | 27.42/6.56 | 28.48/6.24 | 4 | 17% | 475 | *Hordeum vulgare* | 0.58 | 0.79 | R.GVNGTVAHEFIIDLR.G |
|  |  |  |  |  |  |  |  |  |  |  | K.HLAPFLPSHPVIPTGGFPLPEK.T |
|  |  |  |  |  |  |  |  |  |  |  | R.DSPYLTHPIFSMYHTEHELLR.Y |
|  |  |  |  |  |  |  |  |  |  |  | R.NLMENPAWYTQYTPYQAEIAQGR.L |
| L375 | Cytochrome B6-F complex like-protein, partial | gi\|2695939 | 16.980/6.81 | 14.52/6.85 | 4 | 49% | 186 | *Hordeum vulgare* | 0.81 | 0.58 | K.VVFVPWVETDFR.T |
|  |  |  |  |  |  |  |  |  |  |  | K.FLCPCHGSQYNNQGK.V |
|  |  |  |  |  |  |  |  |  |  |  | R.GPAPLSLALVHADVDDGKVVFVPWVETDFR.T |
|  |  |  |  |  |  |  |  |  |  |  | R.GPAPLSLALVHADVDDGKVVFVPWVETDFR.T + 2 Oxidation (HW) |
| L472 | Glycine dehydrogenase | [gi\|473793124](http://www.matrixscience.com/cgi/master_results.pl?file=..%2Fdata%2F20150819%2FFTTASxSST.dat;sessionID=guest_guestsession#Hit3) | 85.81/7.89 | 84.65/7.91 | 4 | 11% | 372 | *Triticum urartu* | 2.06 | 2.86 | R.NGDLKPYAVLWIDAGAR.C |
|  |  |  |  |  |  |  |  |  |  |  | R.NGDLKPYAVLWIDAGAR.C + Deamidated (NQ) |
|  |  |  |  |  |  |  |  |  |  |  | R.YYDPNPGAYPAPAGYGSSR.D |
|  |  |  |  |  |  |  |  |  |  |  | R.VDLDNGENPTWDDKVVVPLPPASR.L |
| **Calvin cycle** | | | | | | | | | | |  |
| L35 | Ribulose-bisphosphate carboxylase oxygenase small subunit | gi\|326509829 | 15.73/8.81 | 13.84/7.81 | 8 | 57% | 303 | *Hordeum vulgare* | 0.62 | 0.44 | K.QVDYLIR.S |
|  |  |  |  |  |  |  |  |  |  |  | R.IIGFDNLR.Q |
|  |  |  |  |  |  |  |  |  |  |  | K.EYPDAYVR.I |
|  |  |  |  |  |  |  |  |  |  |  | K.KFETLSYLPPLSTEALLK.Q |
|  |  |  |  |  |  |  |  |  |  |  | R.EHNSSPGYYDGRYWTMWK.L |
|  |  |  |  |  |  |  |  |  |  |  | R.QVQCVSFIAFRPPGCEESGK.A |
|  |  |  |  |  |  |  |  |  |  |  | K.LPMFGCTDATQVLNEVEEVK.K + Oxidation (M) |
|  |  |  |  |  |  |  |  |  |  |  | R.QVQCVSFIAFRPPGCEESGKA.- |
| L36 | Ribulose bisphosphate carboxylase small chain | gi\|3914588 | 15.69/6.08 | 13.45/5.87 | 10 | 68% | 326 | *Hordeum vulgare* | 0.59 | 0.52 | K.QVDYLIR.S |
|  |  |  |  |  |  |  |  |  |  |  | R.IIGFDNMR.Q + Oxidation (M) |
|  |  |  |  |  |  |  |  |  |  |  | K.EYPDAYVR.I |
|  |  |  |  |  |  |  |  |  |  |  | R.EHNASPGYYDGR.Y |
|  |  |  |  |  |  |  |  |  |  |  | R.EHNASPGYYDGR.Y + Oxidation (HW) |
|  |  |  |  |  |  |  |  |  |  |  | MAPTVMASSATSVAPFQGLK.S |
|  |  |  |  |  |  |  |  |  |  |  | K.KFETLSYLPPLSTEALLK.Q |
|  |  |  |  |  |  |  |  |  |  |  | K.VGFIFREHNASPGYYDGR.Y |
|  |  |  |  |  |  |  |  |  |  |  | K.LPMFGCTDATQVLNEVEEVK.K + Oxidation (M) |
|  |  |  |  |  |  |  |  |  |  |  | R.QVQCVSFIAFKPPGCQESGKA.- + Deamidated (NQ) |
| L252 | Ribulose bisphosphate carboxylase/oxygenase activase B | gi\|10720253 | 47.43/5.59 | 46.54/5.87 | 2 | 7% | 210 | *Hordeum vulgare* | 0.5 | 0.85 | K.GLAYDISDDQQDITR.G |
|  |  |  |  |  |  |  |  |  |  |  | R.LVDTFPGQSIDFFGALR.A |
| L263 | Ribulose-1,5-bisphosphate carboxylase/oxygenase activase small isoform | gi\|313574196 | 47.29/6.59 | 45.84/6.45 | 15 | 35% | 966 | *Hordeum vulgare* | 0.53 | 1.02 | R.VYDDEVR.K |
|  |  |  |  |  |  |  |  |  |  |  | K.FYWAPTR.D |
|  |  |  |  |  |  |  |  |  |  |  | R.VYDDEVRK.W |
|  |  |  |  |  |  |  |  |  |  |  | K.FYWAPTRDDR.I |
|  |  |  |  |  |  |  |  |  |  |  | K.WVGSTGIENIGKR.L |
|  |  |  |  |  |  |  |  |  |  |  | K.GLAYDISDDQQDITR.G |
|  |  |  |  |  |  |  |  |  |  |  | K.MCCLFINDLDAGAGR.M |
|  |  |  |  |  |  |  |  |  |  |  | K.MCCLFINDLDAGAGR.M + Oxidation (M) |
|  |  |  |  |  |  |  |  |  |  |  | K.IVDTFPGQSIDFFGALR.A |
|  |  |  |  |  |  |  |  |  |  |  | K.LLEYGHMLVQEQDNVKR.V |
|  |  |  |  |  |  |  |  |  |  |  | R.VPIVVTGNDFSTLYAPLIR.D |
|  |  |  |  |  |  |  |  |  |  |  | K.LLEYGHMLVQEQDNVKR.V + Oxidation (M) |
|  |  |  |  |  |  |  |  |  |  |  | K.EENPRVPIVVTGNDFSTLYAPLIR.D |
|  |  |  |  |  |  |  |  |  |  |  | K.GIVDSLFQAPTGDGTHEAVLSSYEYVSQGLR.K |
|  |  |  |  |  |  |  |  |  |  |  | R.GKGIVDSLFQAPTGDGTHEAVLSSYEYVSQGLR.K |
| L342 | RuBisCO large subunit-binding protein subunit beta | gi\|2493650 | 56.72/5.37 | 55.89/5.84 | 4 | 10% | 154 | *Hordeum vulgare* | 0.46 | 0.85 | K.AILNLSLR.H |
|  |  |  |  |  |  |  |  |  |  |  | R.HDLHIAAYGEGNER.R |
|  |  |  |  |  |  |  |  |  |  |  | R.VPIVVTGNDFSTLYAPLIR.D |
|  |  |  |  |  |  |  |  |  |  |  | K.VTSQVPWFGIEQEYTLMQR.D + Oxidation (M) |
| L294 | Ribulose-1,5-bisphosphate carboxylase/oxygenase activase small isoform | gi\|313574196 | 47.29/5.59 | 47.95/5.19 | 9 | 28% | 402 | *Hordeum vulgare* | 0.52 | 0.99 | K.FYWAPTR.D |
|  |  |  |  |  |  |  |  |  |  |  | K.FYWAPTR.D + Oxidation (HW) |
|  |  |  |  |  |  |  |  |  |  |  | K.FYWAPTRDDR.I |
|  |  |  |  |  |  |  |  |  |  |  | K.WVGSTGIENIGKR.L |
|  |  |  |  |  |  |  |  |  |  |  | K.GLAYDISDDQQDITR.G |
|  |  |  |  |  |  |  |  |  |  |  | K.IVDTFPGQSIDFFGALR.A |
|  |  |  |  |  |  |  |  |  |  |  | R.VPIVVTGNDFSTLYAPLIR.D |
|  |  |  |  |  |  |  |  |  |  |  | K.LLEYGHMLVQEQDNVKR.V + Oxidation (M) |
|  |  |  |  |  |  |  |  |  |  |  | K.GIVDSLFQAPTGDGTHEAVLSSYEYVSQGLR.K |
| **19** | | | | | | | | | | |  |
| L224 | Cytosolic malate dehydrogenase | gi\|326531984 | 39.92/7.62 | 38.9/7.24 | 5 | 27% | 237 | *Hordeum vulgare* | 0.57 | 0.86 | R.ALGQISER.L |
|  |  |  |  |  |  |  |  |  |  |  | K.EFAPSIPEKNISCLTR.L |
|  |  |  |  |  |  |  |  |  |  |  | R.VLVTGAAGQIGYALVPMIAR.G + Oxidation (M) |
|  |  |  |  |  |  |  |  |  |  |  | R.ELVQDDEWLNGEFIATVQQR.G |
|  |  |  |  |  |  |  |  |  |  |  | K.GVVATTDPVEACTGVNVAVMVGGFPR.K + Oxidation (M) |
| L99 | Carbonate dehydratase | gi\|729003 | 32.74/6.68 | 31.84/6.98 | 5 | 10% | 110 | *Hordeum vulgare* | 0.82 | 0.39 | K.DGADDSFHFVEDWVR.I + 2 Oxidation (HW) |
|  |  |  |  |  |  |  |  |  |  |  | R.VCPSVTLGLEPGEAFTIR.N |
|  |  |  |  |  |  |  |  |  |  |  | K.LVGGHYDFVSGKFETWEQ.- + 2 Oxidation (HW) |
|  |  |  |  |  |  |  |  |  |  |  | K.ALLSLKDGADDSFHFVEDWVR.I |
|  |  |  |  |  |  |  |  |  |  |  | K.ALLSLKDGADDSFHFVEDWVR.I + 2 Oxidation |
| L4 | ATP synthase CF1 beta subunit | gi\|118430395 | 53.89/5.17 | 53.46/5.57 | 15 | 43% | 821 | *Hordeum vulgare* | 0.63 | 1.34 | K.VVDLLAPYR.R |
|  |  |  |  |  |  |  |  |  |  |  | K.VVDLLAPYRR.G |
|  |  |  |  |  |  |  |  |  |  |  | K.AHGGVSVFGGVGER.T |
|  |  |  |  |  |  |  |  |  |  |  | R.IVGNEHYETAQR.V |
|  |  |  |  |  |  |  |  |  |  |  | R.FVQAGSEVSALLGR.M |
|  |  |  |  |  |  |  |  |  |  |  | R.VGLTALTMAEYFR.D + Oxidation (M) |
|  |  |  |  |  |  |  |  |  |  |  | K.LPYIYNALVVQSR.D |
|  |  |  |  |  |  |  |  |  |  |  | K.VALVYGQMNEPPGAR.M + Oxidation (M) |
|  |  |  |  |  |  |  |  |  |  |  | K.QINVTCEVQQLLGNNR.V |
|  |  |  |  |  |  |  |  |  |  |  | R.DVNKQDVLLFIDNIFR.F |
|  |  |  |  |  |  |  |  |  |  |  | K.GIYPAVDPLDSTSTMLQPR.I + Oxidation (M) |
|  |  |  |  |  |  |  |  |  |  |  | R.GMEVIDTGAPLSVPVGGATLGR.I + Oxidation (M) |
|  |  |  |  |  |  |  |  |  |  |  | R.MPSAVGYQPTLSTEMGSLQER.I + 2 Oxidation (M) |
|  |  |  |  |  |  |  |  |  |  |  | R.DTADKQINVTCEVQQLLGNNR.V |
|  |  |  |  |  |  |  |  |  |  |  | R.IFNVLGEPVDNLGPVDSSATFPIHR.S |
| L9 | ATP synthase subunit mitochondrial-like | gi\|326492854 | 51.43/5.85 | 52.45/5.34 | 14 | 37% | 725 | *Hordeum vulgare* | 1.21 | 1.76 | K.THDYLPIHR.E |
|  |  |  |  |  |  |  |  |  |  |  | K.VVDLLAPYQR.G |
|  |  |  |  |  |  |  |  |  |  |  | K.AHGGFSVFAGVGER.T |
|  |  |  |  |  |  |  |  |  |  |  | R.VGLTGLTVAEHFR.D |
|  |  |  |  |  |  |  |  |  |  |  | K.VLNTGSPITVPVGR.A |
|  |  |  |  |  |  |  |  |  |  |  | R.FTQANSEVSALLGR.I |
|  |  |  |  |  |  |  |  |  |  |  | R.LVLEVAQHLGENVVR.T |
|  |  |  |  |  |  |  |  |  |  |  | K.CALVYGQMNEPPGAR.A + Oxidation (M) |
|  |  |  |  |  |  |  |  |  |  |  | R.DAEGQDVLLFIDNIFR.F |
|  |  |  |  |  |  |  |  |  |  |  | R.SISELGIYPAVDPLDSTSR.M |
|  |  |  |  |  |  |  |  |  |  |  | R.IPSAVGYQPTLATDLGGLQER.I |
|  |  |  |  |  |  |  |  |  |  |  | R.FDEGLPPILTALEVLDNSIR.L |
|  |  |  |  |  |  |  |  |  |  |  | K.ITDEFTGAGSVGQVCQVIGAVVDVR.F |
|  |  |  |  |  |  |  |  |  |  |  | K.ITDEFTGAGSVGQVCQVIGAVVDVR.F + Deamidated (NQ) |
| L82 | [ATP synthase subunit beta, mitochondrial](http://blast.ncbi.nlm.nih.gov/Blast.cgi#alnHdr_475548007) | gi\|326532046 | 25.93/5.63 | 25.99/5.78 | 4 | 17% | 180 | *Hordeum vulgare* | 0.61 | 1.35 | R.EYLTFLSGFR.K |
|  |  |  |  |  |  |  |  |  |  |  | K.GTNGTDSEFFNPR.K |
|  |  |  |  |  |  |  |  |  |  |  | R.SEREYLTFLSGFR.K |
|  |  |  |  |  |  |  |  |  |  |  | R.DAAEYVYDVPEGWKER.L |
| L266 | Plastid glutamine synthetase 2 | gi\|755762 | 46.90/5.75 | 47.21/5.35 | 8 | 27% | 302 | *Hordeum vulgare* | 0.63 | 1.07 | R.VGRDTEAK.G |
|  |  |  |  |  |  |  |  |  |  |  | K.AILNLSLR.H |
|  |  |  |  |  |  |  |  |  |  |  | K.GKGYLEDR.R |
|  |  |  |  |  |  |  |  |  |  |  | R.HDLHIAAYGEGNER.R |
|  |  |  |  |  |  |  |  |  |  |  | K.IIAEYIWVGGSGIDLR.S |
|  |  |  |  |  |  |  |  |  |  |  | R.LTGLHETASISDFSWGVANR.G |
|  |  |  |  |  |  |  |  |  |  |  | K.VTSQVPWFGIEQEYTLMQR.D + Oxidation (M) |
|  |  |  |  |  |  |  |  |  |  |  | R.GGNNILVICDTYTPQGEPIPTNKR.H |
| L525 | Glutamine synthetase isoform GS1_1 | gi\|417356959 | 45.33/5.41 | 46.54/5.17 | 8 | 20% | 251 | *Hordeum vulgare* | ↑ | ↑ | K.HKEHIAAYGEGNER.R |
|  |  |  |  |  |  |  |  |  |  |  | K.IIAEYIWIGGSGMDLR.S |
|  |  |  |  |  |  |  |  |  |  |  | K.IIAEYIWIGGSGMDLR.S + Oxidation (M) |
|  |  |  |  |  |  |  |  |  |  |  | K.HETADINTFSWGVANR.G |
|  |  |  |  |  |  |  |  |  |  |  | K.HETADINTFSWGVANR.G + 2 Oxidation (HW) |
|  |  |  |  |  |  |  |  |  |  |  | R.LTGKHETADINTFSWGVANR.G |
|  |  |  |  |  |  |  |  |  |  |  | R.LTGKHETADINTFSWGVANR.G + 2 Oxidation (HW) |
|  |  |  |  |  |  |  |  |  |  |  | K.GNNILVMCDCYTPAGEPIPTNKR.Y |
| L656 | Chain crystal structure of gad1 | gi\|326503536 | 55.47/6.78 | 54.89/6.87 | 2 | 6% | 148 | *Hordeum vulgare* | ↑ | ↑ | K.GSSQVIAQYYQLIR.H |
|  |  |  |  |  |  |  |  |  |  |  | K.EAAYQIINDELMLDGNPR.L |
| L245 | Glutamate-1-semialdehyde - chloroplastic-like | gi\|1170029 | 49.69/6.39 | 49.586.24/ | 11 | 37% | 624 | *Hordeum vulgare* | 0.16 | 0.33 | K.ELMPGGVNSPVR.A |
|  |  |  |  |  |  |  |  |  |  |  | K.IIGGGLPVGAYGGR.K |
|  |  |  |  |  |  |  |  |  |  |  | K.SVGGQPIVFDSVK.G |
|  |  |  |  |  |  |  |  |  |  |  | R.FVNSGTEACMGALR.L |
|  |  |  |  |  |  |  |  |  |  |  | K.FEGCYHGHADSFLVK.A |
|  |  |  |  |  |  |  |  |  |  |  | K.QDGALLVFDEVMTGFR.L |
|  |  |  |  |  |  |  |  |  |  |  | R.GMFGFFFAGGPVHNFDDAK.K |
|  |  |  |  |  |  |  |  |  |  |  | R.LAYGGAQEYFGITPDVTTLGK.I |
|  |  |  |  |  |  |  |  |  |  |  | R.LMEPGTYEYLDKVTGELVR.G |
| **Stress** | | | | | | | | | | | R.EVTKQDGALLVFDEVMTGFR.L |
|  |  |  |  |  |  |  |  |  |  |  | K.GTSFGAPCALENVLAQMVISAVPSIEMVR.F |
| L79 | Adenosine diphosphate glucose pyrophosphatase | gi\|13160411 | 31.96/5.68 | 22.12/5.98 | 1 | 10% | 115 | *Hordeum vulgare* | 0.57 | 0.81 | K.AAVTPAFVGQFPGVNGLGISAAR.L |
| L66 | Adenosine diphosphate glucose pyrophosphatase | gi\|13160411 | 31.96/5.68 | 20.56/5.89 | 1 | 10% | 142 | *Hordeum vulgare* | 0.74 | 1.84 | K.AAVTPAFVGQFPGVNGLGISAAR.L |
| L540 | Adenosine diphosphate glucose pyrophosphatase | gi\|13160411 | 31.96/5.68 | 22.52/6.89 | 1 | 10% | 180 | *Hordeum vulgare* | ↓ | 1.06 | K.AAVTPAFVGQFPGVNGLGISAAR.L |
| L357 | Heat shock protein 70 | gi\|326519769 | 71.37/5.14 | 71.58/5.34 | 1 | 11% | 136 | *Hordeum vulgare* | 1.61 | 1.91 | K.NAVVTVPAYFNDSQR.Q |
| L141 | Glutathione transferase | gi\|326492718 | 28.12/6.59 | 30.32/6.26 | 8 | 49% | 318 | *Hordeum vulgare* | 2.69 | 4.44 | K.GVLGIYEAR.L |
|  |  |  |  |  |  |  |  |  |  |  | R.HASVFDDYPK.V |
|  |  |  |  |  |  |  |  |  |  |  | R.LKGVLGIYEAR.L |
|  |  |  |  |  |  |  |  |  |  |  | K.VFGHPMLTNVAR.V |
|  |  |  |  |  |  |  |  |  |  |  | M.SPVKVFGHPMLTNVAR.V |
|  |  |  |  |  |  |  |  |  |  |  | R.VMLFLEEVGAEYELVPVDFVAGEHK.R |
|  |  |  |  |  |  |  |  |  |  |  | R.YLAGDSISFADLNHIPFTFYFMTTR.H |
|  |  |  |  |  |  |  |  |  |  |  | K.RPQHLQLNPFGQMPGFQDGDLVLFESR.A |
| L83 | Elongation factor p (ef-p) | gi\|326525777 | 26.13/5.77 | 25.89/5.68 | 1 | 4% | 91 | *Hordeum vulgare* | 0.55 | 1.21 | R.IIDFDLPITVR.L |
| L291 | Elongation factor Tu, chloroplastic | [gi\|474198705](http://www.matrixscience.com/cgi/master_results.pl?file=..%2Fdata%2F20150806%2FFTTAonETm.dat;sessionID=guest_guestsession#Hit1) | 45.62/5.61 | 45.78/5.46 | 2 | 9% | 186 | *Triticum urartu* | 0.26 | 0.51 | R.QTDLPFLLAVEDVFSITGR.G |
|  |  |  |  |  |  |  |  |  |  |  | R.ELLTAYEYDGDNVPIVSGSALR.A |
| L299 | Chloroplast translational elongation factor Tu | gi\|6525065 | 48.55/5.8 | 46.81/5.68 | 5 | 16% | 126 | *Hordeum vulgare* | 0.46 | 1.08 | R.IDKLPYSIR.I |
|  |  |  |  |  |  |  |  |  |  |  | K.LAEIPFKPAR.V |
|  |  |  |  |  |  |  |  |  |  |  | K.FYSLPALNDPR.I |
|  |  |  |  |  |  |  |  |  |  |  | R.KHQLLNQQLDNLR.R + 3 Deamidated (NQ); Oxidation (HW) |
|  |  |  |  |  |  |  |  |  |  |  | K.NALENYSYNMR.N + Oxidation (M) |
| L110 | Chaperonin 20 | gi\|326504940 | 30.48/5.62 | 30.23/5.35 | 5 | 54% | 191 | *Hordeum vulgare* | 0.36 | 0.62 | K.GTDGTNYIVLR.V |
|  |  |  |  |  |  |  |  |  |  |  | K.YAGGEFKGTDGTNYIVLR.V |
|  |  |  |  |  |  |  |  |  |  |  | K.HLIMKEDDIIGILESDDVK.D |
|  |  |  |  |  |  |  |  |  |  |  | K.EKPSIGTVVAVGPGSLDEEGNR.Q |
|  |  |  |  |  |  |  |  |  |  |  | K.EKPSIGTVVAVGPGSLDEEGNRQPLSVSPGSTVLYSK.Y |
| L45 | Strong similarity to 50S ribosomal protein L28 | gi\|1903348 | 16.17/5.93 | 16.78/6.12 | 4 | 22% | 93 | *Hordeum vulgare* | 0.51 | 0.68 | R.TTPSYVAFTDSER.L |
|  |  |  |  |  |  |  |  |  |  |  | R.FEEMNMDLFRK.C + Oxidation (M) |
|  |  |  |  |  |  |  |  |  |  |  | K.ATAGDTHLGGEDFDNR.M |
|  |  |  |  |  |  |  |  |  |  |  | K.NQVAMNPINTVFDAKR.L + Oxidation (M) |
|  |  |  |  |  |  |  |  |  |  |  |  |
| a The number of spot in gels indicates differentially expressed protein as given in Fig. S2; | | | | | |  |  |  |  |  |  |
| b Names and species of the proteins obtained via the MASCOT software from the NCBInr database | | | | | | | | | | | |
| c Accession number from the NCBInr database; | | | | | | | | | | | |
| d Theoretical molecular weight and isoelectric point | | | | | | | | | | | |
| e Experimental molecular weight and isoelectric point | | | | | | | | | | | |
| f Number of query matced pettides | | | | | | | | | | | |
| g Sequence coverage | | | | | | | | | | | |
| h Statistical probability of the predicted protein is calculated by MASCOT | | | | | | | | | | | |
| i The fold change is calculated using the mean value of each spot in normalized spot volume from gels comparing under control and waterlogging conditions.↑ and ↓ indicate specifically expressed protein under control and waterlogging conditions, respectively. | | | | | | | | | | | |

| **Table S3** The differentially expressed proteins of adventitious roots between TF57 and TF58 under control and waterlogging stress | | | | | | | | | | | |
| --- | --- | --- | --- | --- | --- | --- | --- | --- | --- | --- | --- |
|  |  |  |  |  |  |  |  |  |  |  |  |
| **Spot No** | **Protein Name** | **Protein ID^a^** | **Theoretical Mr(kDa)/p*I^b^*** | **Experimental Mr(kDa)/p*I^b^*** | **Matched Peptide^c^** | **Cov (%)^d^** | **Score^e^** | **Species** | **Change folds** | | **Peptides Squence** |
|  |  |  |  |  |  |  |  |  | **TF57** | **TF58** |  |
| **Energy** | | | | | | | | | | |  |
| AR610 | Phosphoglucomutase | gi\|326504468 | 62.95/5.28 | 63.54/5.31 | 26% | 9 | 408 | *Hordeum vulgare* | 0.43 | 0.88 | R.DTTPYEGQKPGTSGLR.K |
|  |  |  |  |  |  |  |  |  |  |  | K.FSFCFDGLHGVAGAYAK.R |
|  |  |  |  |  |  |  |  |  |  |  | K.LVTVEDIVLQHWGTYGR.H |
|  |  |  |  |  |  |  |  |  |  |  | R.VWVGQDSLLSTPAVSAIIR.E |
|  |  |  |  |  |  |  |  |  |  |  | R.RVWVGQDSLLSTPAVSAIIR.E |
|  |  |  |  |  |  |  |  |  |  |  | K.ATGAFILTASHNPGGPTEDFGIK.Y |
|  |  |  |  |  |  |  |  |  |  |  | K.DNLGGDKLVTVEDIVLQHWGTYGR.H |
|  |  |  |  |  |  |  |  |  |  |  | K.FFGNLMDAGMCSVCGEESFGTGSDHIR.E |
|  |  |  |  |  |  |  |  |  |  |  | K.VTVFQQPHYLANFVQSTFNALPADQVK.G |
| AR949 | Triosephosphate isomerase | gi\|326527869 | 26.99/5.39 | 27.16/5.39 | 54% | 7 | 191 | *Hordeum vulgare* | ↓ | ↓ | K.LRPEIQVAAQNCWVK.K |
|  |  |  |  |  |  |  |  |  |  |  | K.VATPAQAQEVHANLRDWLK.T |
|  |  |  |  |  |  |  |  |  |  |  | K.VATPAQAQEVHANLRDWLK.T + 2 Oxidation (HW) |
|  |  |  |  |  |  |  |  |  |  |  | K.IKDWSNVVVAYEPVWAIGTGK.V + 2 Oxidation (HW) |
|  |  |  |  |  |  |  |  |  |  |  | R.SLLGESSEFVGEKVAYALAQGLK.V |
|  |  |  |  |  |  |  |  |  |  |  | K.GGAFTGEVSAEMLANLGVPWVILGHSER.R |
|  |  |  |  |  |  |  |  |  |  |  | K.ELAAQADVDGFLVGGASLKPEFIDIINAAAVK.S |
| AR585 | Pyruvate decarboxylase | gi\|326528981 | 65.83/5.62 | 64.34/5.78 | 31% | 9 | 775 | *Hordeum vulgare* | 4.17 | 5.85 | R.DPVPFFLAPR.M |
|  |  |  |  |  |  |  |  |  |  |  | R.ILHHTIGVPDFSQELR.C |
|  |  |  |  |  |  |  |  |  |  |  | K.DSLCFIEVIAHKDDTSK.E + Oxidation (HW) |
|  |  |  |  |  |  |  |  |  |  |  | R.IFVPEGHPLKGEANEPLR.V |
|  |  |  |  |  |  |  |  |  |  |  | R.LVGCCNELNAGYAADGYAR.A |
|  |  |  |  |  |  |  |  |  |  |  | R.VIACIGDGSFQVTAQDVSTMLR.C |
|  |  |  |  |  |  |  |  |  |  |  | R.ESKPVYLSISCNLPGLPHPTFTR.D |
|  |  |  |  |  |  |  |  |  |  |  | R.LVQVGVSDVFAVPGDFNLTLLDHLVAEPGLR.L |
|  |  |  |  |  |  |  |  |  |  |  | R.CFQTVTCHQAVVTNLDDAHEQIDTAIATALR.E |
| AR168 | Germin-like protein 8-9-like | gi\|326503884 | 28.89/6.58 | 29.54/6.67 | 25% | 3 | 327 | *Hordeum vulgare* | 0.19 | 0.24 | R.IDYAPLGQNPPHTHPR.A |
|  |  |  |  |  |  |  |  |  |  |  | K.NTIDWLQAQFWENNHN. |
|  |  |  |  |  |  |  |  |  |  |  | R.ATEILTVLEGTLYVGFVTSNLPAPNR.N |
| AR595 | NADP-dependent malic enzyme | gi\|209405432 | 63.31/5.57 | 65.48/5.82 | 22% | 10 | 686 | *Hordeum vulgare* | 1.11 | 3.33 | R.QYTVPLQR.Y |
|  |  |  |  |  |  |  |  |  |  |  | R.YIAMMDLQER.N |
|  |  |  |  |  |  |  |  |  |  |  | K.GLIFPPFTNIR.K |
|  |  |  |  |  |  |  |  |  |  |  | K.YAESCMYTPLYR.S |
|  |  |  |  |  |  |  |  |  |  |  | R.ATGDEYHELLQEFMTAVK.Q |
|  |  |  |  |  |  |  |  |  |  |  | K.VLVQFEDFANHNAFDLLAK.Y |
|  |  |  |  |  |  |  |  |  |  |  | R.RATGDEYHELLQEFMTAVK.Q |
|  |  |  |  |  |  |  |  |  |  |  | R.ATEEQLVTPWSFSVASGHSLLR.D |
|  |  |  |  |  |  |  |  |  |  |  | K.TYVPGQSNNAYVFPGFGLGVVISGAIR.V |
|  |  |  |  |  |  |  |  |  |  |  | R.ATEEQLVTPWSFSVASGHSLLRDPR.H |
| AR17 | NADP-dependent malic enzyme | gi\|209405432 | 63.31/5.57 | 63.45/5.48 | 13% | 4 | 352 | *Hordeum vulgare* | 1.45 | 2.91 | K.GLIFPPFTNIR.K |
|  |  |  |  |  |  |  |  |  |  |  | R.ATGDEYHELLQEFMTAVK.Q |
|  |  |  |  |  |  |  |  |  |  |  | R.ATEEQLVTPWSFSVASGHSLLRDPR.H |
|  |  |  |  |  |  |  |  |  |  |  | K.TYVPGQSNNAYVFPGFGLGVVISGAIR.V |
|  |  |  |  |  |  |  |  |  |  |  | K.VLVQFEDFANHNAFDLLAK.Y |
|  |  |  |  |  |  |  |  |  |  |  | R.RATGDEYHELLQEFMTAVK.Q |
|  |  |  |  |  |  |  |  |  |  |  | R.ATEEQLVTPWSFSVASGHSLLR.D |
|  |  |  |  |  |  |  |  |  |  |  | R.ATEEQLVTPWSFSVASGHSLLR.D + 2 Oxidation (HW) |
|  |  |  |  |  |  |  |  |  |  |  | K.TYVPGQSNNAYVFPGFGLGVVISGAIR.V |
| **N-metabolism** | | | | | | | | | | |  |
| AR400 | Glutamine synthetase isoform GS1_1 | gi\|417356959 | 39.33/5.31 |  | 49% | 8 | 450 | *Hordeum vulgare* | 2.05 | 2.61 | K.HKEHIAAYGEGNER.R |
|  |  |  |  |  |  |  |  |  |  |  | K.IIAEYIWIGGSGMDLR.S |
|  |  |  |  |  |  |  |  |  |  |  | K.IIAEYIWIGGSGMDLR.S + Oxidation (M) |
|  |  |  |  |  |  |  |  |  |  |  | K.HETADINTFSWGVANR.G |
|  |  |  |  |  |  |  |  |  |  |  | K.HETADINTFSWGVANR.G + 2 Oxidation (HW) |
|  |  |  |  |  |  |  |  |  |  |  | R.LTGKHETADINTFSWGVANR.G |
|  |  |  |  |  |  |  |  |  |  |  | R.LTGKHETADINTFSWGVANR.G + 2 Oxidation (HW) |
|  |  |  |  |  |  |  |  |  |  |  | K.GNNILVMCDCYTPAGEPIPTNKR.Y |
| AR373 | Glutamine synthetase isoform GS1_2 | gi\|417356961 | 39.04/5.71 | 39.84/5.78 | 43% | 7 | 500 | *Hordeum vulgare* | 2.91 | 4.54 | R.HMQHIAAYGEGNER.R |
|  |  |  |  |  |  |  |  |  |  |  | K.VIVEYLWVGGSGIDIR.S |
|  |  |  |  |  |  |  |  |  |  |  | K.VAAEETWYGIEQEYTLLQK.D |
|  |  |  |  |  |  |  |  |  |  |  | R.RPASNMDPYVVTSMIAETTLLL.- |
|  |  |  |  |  |  |  |  |  |  |  | R.GDNILVMCDCYTPQGVPIPTNKR.H |
|  |  |  |  |  |  |  |  |  |  |  | K.DVNWPLGWPIGGYPGPQGPYYCAAGADK.A |
|  |  |  |  |  |  |  |  |  |  |  | K.WNYDGSSTGQAPGEDSEVILYPQAIFKDPFR.R |
| AR421 | Plastid glutamine synthetase 2 | gi\|755762 | 46.90/5.75 | 45.78/5.48 | 25% | 7 | 363 | *Hordeum vulgare* | 0.28 | 0.79 | K.IIAEYIWVGGSGIDLR.S |
|  |  |  |  |  |  |  |  |  |  |  | R.LTGLHETASISDFSWGVANR.G + 2 Oxidation (HW) |
|  |  |  |  |  |  |  |  |  |  |  | K.VTSQVPWFGIEQEYTLMQR.D |
|  |  |  |  |  |  |  |  |  |  |  | K.VTSQVPWFGIEQEYTLMQR.D + Oxidation (M) |
|  |  |  |  |  |  |  |  |  |  |  | K.VTSQVPWFGIEQEYTLMQR.D + Oxidation (HW); |
|  |  |  |  |  |  |  |  |  |  |  | R.GGNNILVICDTYTPQGEPIPTNKR.H |
| **Lipid metabolism** | | | | | | | | | | | K.WNYDGSSTGQAPGEDSEVILYPQAIFKDPFR.G |
| AR689 | Quinone reductase 2 | gi\|326516502 | 27.77/6.21 | 27.89/6.54 | 54% | 6 | 765 | *Hordeum vulgare* | 2.06 | 0.39 | K.AFFDATGGLWR.E |
|  |  |  |  |  |  |  |  |  |  |  | K.VWQVPEILNEEVLGK.M |
|  |  |  |  |  |  |  |  |  |  |  | R.WPSEMELEHAFHQGK.Y |
|  |  |  |  |  |  |  |  |  |  |  | M.AVKVYVVYYSMYGHVAK.L + Oxidation (M) |
|  |  |  |  |  |  |  |  |  |  |  | K.TDVPVISPQELAEADGVLFGFPTR.F |
|  |  |  |  |  |  |  |  |  |  |  | K.MGAPPKTDVPVISPQELAEADGVLFGFPTR.F |
| AR333 | Gibberellin receptor gid1l2 | gi\|326511611 | 38.79/5.61 | 37.54/5.78 | 20% | 5 | 328 | *Hordeum vulgare* | 0.75 | 0.36 | K.DVVIDPANGLWAR.V |
|  |  |  |  |  |  |  |  |  |  |  | R.VFLAGGSAGGTIAHVMAVR.A |
|  |  |  |  |  |  |  |  |  |  |  | R.LAPEHPLPAAYDDSWEGLK.W |
|  |  |  |  |  |  |  |  |  |  |  | R.FLYPGSPGLDDPLSNPFSEAAGGSAAR.I |
|  |  |  |  |  |  |  |  |  |  |  | K.ASGYAGEVELLESVGEDHVFYCMKPR.S |
| AR311 | Sulfotransferase 17-like | gi\|326521606 | 37.06/6.02 | 36.54/6.08 | 30% | 8 | 597 | *Hordeum vulgare* | 0.47 | 0.41 | K.IFIQHSSLFR.K |
|  |  |  |  |  |  |  |  |  |  |  | R.SRYDFADADHPLR.T |
|  |  |  |  |  |  |  |  |  |  |  | K.SGVVDQVVSFCSFESLR.N |
|  |  |  |  |  |  |  |  |  |  |  | R.EGWSTPLTLHNNFWLR.S |
|  |  |  |  |  |  |  |  |  |  |  | R.EGWSTPLTLHNNFWLR.S + Oxidation (HW) |
|  |  |  |  |  |  |  |  |  |  |  | R.EGWSTPLTLHNNFWLR.S + 2 Oxidation (HW) |
|  |  |  |  |  |  |  |  |  |  |  | R.LLSTHLPLSLLPPAVSAVGCR.V |
|  |  |  |  |  |  |  |  |  |  |  | R.VVPFIGAVGGDLDFLETLPSPR.L |
| AR152 | Quinone reductase 2 of the maize glutamine synthetase | gi\|326512488 | 25.69/5.94 | 25.89/6.12 | 39% | 4 | 271 | *Hordeum vulgare* | 0.38 | 0.32 | K.AFFDATGGLWR.E |
|  |  |  |  |  |  |  |  |  |  |  | K.LFDMDKVQGGSPYGAGTFAADGSR.W |
|  |  |  |  |  |  |  |  |  |  |  | K.LDAPIITPQELADADGILFGFPTR.F |
|  |  |  |  |  |  |  |  |  |  |  | R.WPSEMELEHAFHQGQYFAGIAK.K |
| **Hormone metabolism** | | | | | | | | | | |  |
| AR306 | Isoflavone reductase homolog | [gi\|474219963](http://www.matrixscience.com/cgi/master_results.pl?file=..%2Fdata%2F20150807%2FFTTAoGHaS.dat;sessionID=guest_guestsession#Hit1) | 36.24/5.45 | 35.78/5.54 | 75% | 1 | 162 | *Triticum urartu* | 0.41 | 0.39 | K.QIQEAPIPMNIIFSIGHASYIK.G |
| AR852 | 1-aminocyclopropane-1-carboxylate oxidase 1-like | gi\|397740906 | 34.80/5.17 | 38.14/5.19 | 51% | 10 | 1060 | *Hordeum vulgare* | ↑ | ↑ | K.AVSGEVDWETAYFIR.H |
|  |  |  |  |  |  |  |  |  |  |  | R.EMLDVYIGQMVSLAER.L |
|  |  |  |  |  |  |  |  |  |  |  | R.IFVNLGDQLEVMSGGAYR.S |
|  |  |  |  |  |  |  |  |  |  |  | R.HRPANNVADFPEIPPATR.E |
|  |  |  |  |  |  |  |  |  |  |  | K.FAMYPACPRPDLLWGLR.A |
|  |  |  |  |  |  |  |  |  |  |  | K.FAMYPACPRPDLLWGLR.A + Oxidation (M) |
|  |  |  |  |  |  |  |  |  |  |  | R.LSVATFYNPGAEAVVAPAPTAR.Q |
|  |  |  |  |  |  |  |  |  |  |  | R.AHTDAGGIILLLQDDVVGGLEFFR.G |
|  |  |  |  |  |  |  |  |  |  |  | R.AHTDAGGIILLLQDDVVGGLEFFRGDR.E |
|  |  |  |  |  |  |  |  |  |  |  | R.LHEACKDWGFFWVDSHGVDAALMEEVK.R |
| AR402 | 12-oxophytodienoate reductase 5-like | gi\|326516290 | 40.77/5.96 | 42.17/6.15 | 14% | 4 | 252 | *Hordeum vulgare* | 1.79 | 2.05 | K.GAAFFCQLWHVGR.V |
|  |  |  |  |  |  |  |  |  |  |  |  |
|  |  |  |  |  |  |  |  |  |  |  | K.LNDHGILYLHMIEPR.M |
|  |  |  |  |  |  |  |  |  |  |  | R.SYGNVPQPHAAVYYGQR.A |
|  |  |  |  |  |  |  |  |  |  |  | R.NAIDAGFDGVEIHGGNGYLIEQFLK.D |
| AR14 | Ascorbate peroxidase | gi\|257696322 | 27.53/5.85 | 28.89/5.89 | 38% | 5 | 491 | *Hordeum vulgare* | 1.32 | 1.59 | R.SGFEGPWTR.N |
|  |  |  |  |  |  |  |  |  |  |  | K.AFFEDYKEAHLR.L |
|  |  |  |  |  |  |  |  |  |  |  | K.TLLTDPVFRPLVEK.Y |
|  |  |  |  |  |  |  |  |  |  |  | K.KPAEQAHAANAGLDIAVR.M |
|  |  |  |  |  |  |  |  |  |  |  | K.SYPVVSAEYLEAVEKAR.Q |
| AR169 | Dehydroascorbate reductase | gi\|326496021 | 26.44/5.71 | 26.84/5.68 | 66% | 13 | 959 | *Hordeum vulgare* | 0.55 | 0.33 | K.IFSTFVTFLK.S |
|  |  |  |  |  |  |  |  |  |  |  | K.LFHLQVALEHFK.G |
|  |  |  |  |  |  |  |  |  |  |  | K.LIDVSNKPDWFLK.I |
|  |  |  |  |  |  |  |  |  |  |  | K.ALVDELQALEEHLK.A |
|  |  |  |  |  |  |  |  |  |  |  | K.WIADSDVITQVIEEK.Y |
|  |  |  |  |  |  |  |  |  |  |  | K.AAVGHPDTLGDCPFSQR.V |
|  |  |  |  |  |  |  |  |  |  |  | K.LFHLQVALEHFKGWK.V |
|  |  |  |  |  |  |  |  |  |  |  | K.YPTPSLVTPPEYASVGSK.I |
|  |  |  |  |  |  |  |  |  |  |  | K.VPETLTSVHAYTEALFSR.E |
|  |  |  |  |  |  |  |  |  |  |  | K.AHGPYINGANVSAADLSLAPK.L |
|  |  |  |  |  |  |  |  |  |  |  | K.AHGPYINGANVSAADLSLAPK.L + Deamidated (NQ) |
|  |  |  |  |  |  |  |  |  |  |  | K.GWKVPETLTSVHAYTEALFSR.E |
|  |  |  |  |  |  |  |  |  |  |  | K.WIADSDVITQVIEEKYPTPSLVTPPEYASVGSK.I |
| **Development** | | | | | | | | | | |  |
| AR343 | 11s globulin seed storage protein 2-like | gi\|326516970 | 35.91/5.1 | 37.54/5.35 | 10% | 2 | 64 | *Hordeum vulgare* | 0.3 | 0.43 | R.LEAGDVIAVR.T |
|  |  |  |  |  |  |  |  |  |  |  | R.EGAAQAVYVAR.G |
| AR144 | Translationally-controlled tumor protein | gi\|20140865 | 25.93/4.53 | 26.24/4.58 | 37% | 4 | 257 | *Hordeum vulgare* | 3.1 | 0.41 | K.VVDIVDTFR.L |
|  |  |  |  |  |  |  |  |  |  |  | K.LSGDELLSDSFPYR.E |
|  |  |  |  |  |  |  |  |  |  |  | R.EGAADPTFLYFAHGLK.E |
|  |  |  |  |  |  |  |  |  |  |  | K.LKDLQFFVGESMHDDGSVVFAYYR.E |
| **Stress** | | | | | | | | | | |  |
|  | Germin-like protein | gi\|9837119 | 25.40/7.15 | 26.21/7.18 | 29% | 3 | 302 | *Hordeum vulgare* | 0.06 | 0.06 | R.IDYGPLGVNTPHIHPR.A |
|  |  |  |  |  |  |  |  |  |  |  | K.LIDWLQSQFWENNHY.- + Deamidated (NQ) |
|  |  |  |  |  |  |  |  |  |  |  | R.ATELLTVLEGTLYLGFVTSNPNR.L |
| AR148 | Glutathione s-transferase-like | gi\|326510031 | 23.98/5.47 | 25.48/5.68 | 25% | 2 | 154 | *Hordeum vulgare* | 2.96 | 1.45 | K.YCVGDEVHLGDVFLAPQIHAAINR.F |
|  |  |  |  |  |  |  |  |  |  |  | K.ALDLQIANIVCSSIQPLQGYGVIGLHEGR.L |
| AR181 | Glutathione transferase | gi\|326532030 | 27.01/5.82 | 27.14/6.01 | 9% | 1 | 198 | *Hordeum vulgare* | 2.29 | 1.08 | R.NPFGQIPAFQDGDLLLFESR.A |
| AR694 | Glutathione s-transferase | gi\|326529393 | 27.54/6.46 | 28.01/6.89 | 37% | 6 | 131 | *Hordeum vulgare* | 1.68 | 3.01 | K.NLEFELVR.I |
|  |  |  |  |  |  |  |  |  |  |  | R.EHKLPEFIK.L + Oxidation (HW) |
|  |  |  |  |  |  |  |  |  |  |  | R.YLCTQFPEDGNR.G |
|  |  |  |  |  |  |  |  |  |  |  | R.WYDAISSRPSWK.Q + 2 Oxidation (HW) |
|  |  |  |  |  |  |  |  |  |  |  | K.LQQMLGVYDEILAK.N + 2 Deamidated (NQ); Oxidation (M) |
|  |  |  |  |  |  |  |  |  |  |  | K.NQYLAGDEFTLADLSHLPASHYIAGSQR.G |
| AR141 | Thioredoxin-like protein 1 | gi\|326514956 | 25.08/4.8 | 25.16/4.86 | 16% | 2 | 1 | *Hordeum vulgare* | ↓ | ↓ | K.LHSALFKGPEEEGPK.T |
|  |  |  |  |  |  |  |  |  |  |  | K.QGYREDEGLHLASDSDEQLLIYIPFMQVIK.L |
| AR370 | Caffeic acid o-methyltransferase | gi\|326505616 | 40.17/5.31 | 41.52/5.35 | 29% | 9 | 627 | *Hordeum vulgare* | 1.18 | ↑ | R.LSGFDGAFR.S |
|  |  |  |  |  |  |  |  |  |  |  | R.LLAAFDVVR.C |
|  |  |  |  |  |  |  |  |  |  |  | R.TEQDFVNMAR.L |
|  |  |  |  |  |  |  |  |  |  |  | K.TAVELGLIDALTNAAGR.A |
|  |  |  |  |  |  |  |  |  |  |  | K.VIVVEIVLPATTEATR.E |
|  |  |  |  |  |  |  |  |  |  |  | R.EAQDMFLLDVIMFNNLEGGKER.T |
|  |  |  |  |  |  |  |  |  |  |  | R.EAQDMFLLDVIMFNNLEGGKER.T + Oxidation (M) |
|  |  |  |  |  |  |  |  |  |  |  | R.FDGFDGVGVLVDVGGGTGAALEMITSR.H |
|  |  |  |  |  |  |  |  |  |  |  | R.FDGFDGVGVLVDVGGGTGAALEMITSR.H + Oxidation (M) |
| AR679 | r40c1 protein | gi\|326497973 | 19.06/6.27 | 18.79/6.78 | 18% | 3 | 184 | *Hordeum vulgare* | 0.26 | 0.34 | K.ILPWGEEAYAGGSANAPR.G |
|  |  |  |  |  |  |  |  |  |  |  | R.DGNVVLAPSNPRDEHQHWFK.D |
|  |  |  |  |  |  |  |  |  |  |  | K.DHGGVHDGTTVVLWEWAKGDNQSWK.I + 2 Deamidated (NQ); 3 Oxidation (HW) |
| AR677 | Cold-regulated protein | gi\|326511755 | 17.66/4.93 | 17.54/5.12 | 49% | 3 | 232 | *Hordeum vulgare* | ↓ | 0.32 | K.DISPPLEWYGVPGGAR.S |
|  |  |  |  |  |  |  |  |  |  |  | K.GLPEGFSGAGGNANAGGEGGLQEGVNDWKQPGWR.G |
|  |  |  |  |  |  |  |  |  |  |  | R.SLALVVQDIDADERVPWTHWVVANISPEEK.G |
| AR97 | Wound stress protein precursor | gi\|326526311 | 20.07/5.47 | 15.45/5.35 | 18% | 2 | 104 | *Hordeum vulgare* | 0.36 | 1.14 | R.GNLDIFSGR.G |
|  |  |  |  |  |  |  |  |  |  |  | R.VSSDGTGAHHGWYCNYVEVTVTGPHR.G |
| **Protein** | | | | | | | | | | |  |
|  |  |  |  |  |  |  |  |  |  |  |  |
| AR321 | Guanine nucleotide-binding protein subunit beta-like protein a-like | gi\|326491885 | 36.66/5.97 | 37.21/6.27 | 41% | 7 | 423 | *Hordeum vulgare* | 1.37 | 3.25 | R.FSPNNFAPTIVSGSWDR.S |
|  |  |  |  |  |  |  |  |  |  |  | R.LYSLDAGSIINSLCFSPNR.Y |
|  |  |  |  |  |  |  |  |  |  |  | K.YTIGGDLGGGEGHTGWVSCVR.F |
|  |  |  |  |  |  |  |  |  |  |  | R.GHNDVVTAIATPIDNSPFIVSSSR.D |
|  |  |  |  |  |  |  |  |  |  |  | K.SLLVWDLTNPIQATQDTSSEYGVPFR.R |
|  |  |  |  |  |  |  |  |  |  |  | R.DKSLLVWDLTNPIQATQDTSSEYGVPFR.R |
|  |  |  |  |  |  |  |  |  |  |  | R.LTGHGHFVQDVVLSSDGQFALSGSWDGELR.L |
|  |  |  |  |  |  |  |  |  |  |  |  |
| a The number of spot in gels indicates differentially expressed protein as given in Fig. S2; | | | | |  |  |  |  |  |  |  |
| b Names and species of the proteins obtained via the MASCOT software from the NCBInr database | | | | | | | | | | | |
| c Accession number from the NCBInr database; | | | | | | | | | | | |
| d Theoretical molecular weight and isoelectric point | | | | | | | | | | | |
| e Experimental molecular weight and isoelectric point | | | | | | | | | | | |
| f Number of query matced pettides | | | | | | | | | | | |
| g Sequence coverage | | | | | | | | | | | |
| h Statistical probability of the predicted protein is calculated by MASCOT | | | | | | | | | | | |
| i The fold change is calculated using the mean value of each spot in normalized spot volume from gels comparing under control and waterlogging conditions.↑ and ↓ indicate specifically expressed protein under control and waterlogging conditions, respectively. | | | | | | | | | | | |
|  |  |  |  |  |  |  |  |  |  |  |  |

| **Table S4** The differentially expressed proteins of nodal roots between TF57 and TF58 under control and waterlogging stress | | | | | | | | | | | |
| --- | --- | --- | --- | --- | --- | --- | --- | --- | --- | --- | --- |
|  |  |  |  |  |  |  |  |  |  |  |  |
| **Spot No** | **Protein Name** | **Protein ID^a^** | **Theoretical Mr(kDa)/p*I^b^*** | **Experimental Mr(kDa)/p*I^b^*** | **Matched peptide^c^** | **Cov (%)^d^** | **Score^e^** | **Species** | **Change folds** | | **Peptides Squence** |
|  |  |  |  |  |  |  |  |  | **TF57** | **TF58** |  |
| **Energy** | | | | | | | | | | |  |
| NR779 | Fruit protein pkiwi 502-like | gi\|326522216 | 36.14/6.45 | 30.54/5.78 | 5 | 33% | 125 | *Hordeum vulgare* | ↑ | 1.02 | R.ALIESGFSASQR.A |
|  |  |  |  |  |  |  |  |  |  |  | K.DMQSMPYQER.F |
|  |  |  |  |  |  |  |  |  |  |  | K.ILTIEWNPDIDLSTLDSFGLNGLGLVR.R |
|  |  |  |  |  |  |  |  |  |  |  | R.IPSLLGGPPPAFMCISSPPHSGLQFDLLVR.S |
|  |  |  |  |  |  |  |  |  |  |  | K.GTGFAIQNINPPEDTETVLLFAAAEGISPIR.A |
| NR1132 | Glyceraldehyde-3-phosphate dehydrogenase 2, cytosolic | gi\|120668 | 33.44/6.2 | 36.54/6.54 | 7 | 33% | 328 | *Hordeum vulgare* | 4.05 | 1.34 | K.TLLFGEKEVAVFGCR.N |
|  |  |  |  |  |  |  |  |  |  |  | K.LVSWYDNEWGYSTR.V |
|  |  |  |  |  |  |  |  |  |  |  | K.DAPMFVCGVNEKEYK.S + Deamidated (NQ) |
|  |  |  |  |  |  |  |  |  |  |  | K.LVSWYDNEWGYSTR.V + 2 Oxidation (HW) |
|  |  |  |  |  |  |  |  |  |  |  | K.YDTVHGQWKHHEVK.V + 3 Oxidation (HW) |
|  |  |  |  |  |  |  |  |  |  |  | K.GILGYVDEDLVSTDFQGDSR.S |
|  |  |  |  |  |  |  |  |  |  |  | K.VINDRFGIVEGLMTTVHAMTATQK.T |
| NR864 | Pyruvate decarboxylase | gi\|326528981 | 65.83/5.62 | 66.21/5.67 | 9 | 31% | 775 | *Hordeum vulgare* | 1.38 | 3.17 | R.DPVPFFLAPR.M |
|  |  |  |  |  |  |  |  |  |  |  | R.ILHHTIGVPDFSQELR.C |
|  |  |  |  |  |  |  |  |  |  |  | K.DSLCFIEVIAHKDDTSK.E + Oxidation (HW) |
|  |  |  |  |  |  |  |  |  |  |  | R.IFVPEGHPLKGEANEPLR.V |
|  |  |  |  |  |  |  |  |  |  |  | R.LVGCCNELNAGYAADGYAR.A |
|  |  |  |  |  |  |  |  |  |  |  | R.VIACIGDGSFQVTAQDVSTMLR.C |
|  |  |  |  |  |  |  |  |  |  |  | R.ESKPVYLSISCNLPGLPHPTFTR.D |
|  |  |  |  |  |  |  |  |  |  |  | R.LVQVGVSDVFAVPGDFNLTLLDHLVAEPGLR.L |
|  |  |  |  |  |  |  |  |  |  |  | R.CFQTVTCHQAVVTNLDDAHEQIDTAIATALR.E |
| NR709 | NADP-dependent malic enzyme | gi\|209405432 | 63.31/5.57 | 67.35/5.69 | 10 | 14% | 686 | *Hordeum vulgare* | 1.06 | 2.28 | R.QYTVPLQR.Y |
|  |  |  |  |  |  |  |  |  |  |  | R.YIAMMDLQER.N |
|  |  |  |  |  |  |  |  |  |  |  | K.GLIFPPFTNIR.K |
|  |  |  |  |  |  |  |  |  |  |  | K.YAESCMYTPLYR.S |
|  |  |  |  |  |  |  |  |  |  |  | R.ATGDEYHELLQEFMTAVK.Q |
|  |  |  |  |  |  |  |  |  |  |  | K.VLVQFEDFANHNAFDLLAK.Y |
|  |  |  |  |  |  |  |  |  |  |  | R.RATGDEYHELLQEFMTAVK.Q |
|  |  |  |  |  |  |  |  |  |  |  | R.ATEEQLVTPWSFSVASGHSLLR.D |
|  |  |  |  |  |  |  |  |  |  |  | K.TYVPGQSNNAYVFPGFGLGVVISGAIR.V |
|  |  |  |  |  |  |  |  |  |  |  | R.ATEEQLVTPWSFSVASGHSLLRDPR.H |
| NR1032 | ATP synthase d mitochondrial | gi\|326524003 | 19.60/6.22 | 18.98/6.02 | 1 | 8% | 92 | *Hordeum vulgare* | 1.23 | ↑ | K.FSQEPQPIDWEYYR.K |
| **Metabolism** | | | | | | | | | | |  |
| NR11 | Glutamine synthetase isoform GS1_2 | gi\|417356961 | 39.04/5.71 | 39.21/5.69 | 7 | 43% | 500 | *Hordeum vulgare* | 0.52 | 0.77 | R.HMQHIAAYGEGNER.R |
|  |  |  |  |  |  |  |  |  |  |  | K.VIVEYLWVGGSGIDIR.S |
|  |  |  |  |  |  |  |  |  |  |  | K.VAAEETWYGIEQEYTLLQK.D |
|  |  |  |  |  |  |  |  |  |  |  | R.RPASNMDPYVVTSMIAETTLLL.- |
|  |  |  |  |  |  |  |  |  |  |  | R.GDNILVMCDCYTPQGVPIPTNKR.H |
|  |  |  |  |  |  |  |  |  |  |  | K.DVNWPLGWPIGGYPGPQGPYYCAAGADK.A |
|  |  |  |  |  |  |  |  |  |  |  | K.WNYDGSSTGQAPGEDSEVILYPQAIFKDPFR.R |
| NR370 | Isoflavone reductase homolog | [gi\|474219963](http://www.matrixscience.com/cgi/master_results.pl?file=..%2Fdata%2F20150807%2FFTTAoGHaS.dat;sessionID=guest_guestsession#Hit1) | 24.24/6.45 | 35.42/5.42 | 1 | 75% | 162 | *Triticum urartu* | 0.55 | 0.76 | K.QIQEAPIPMNIIFSIGHASYIK.G |
| NR412 | 1-aminocyclopropane-1-carboxylate oxidase 1-like | gi\|397740906 | 34.80/5.17 | 36.78/5.12 | 10 | 51% | 1060 | *Hordeum vulgare* | 1.65 | 5.2 | K.AVSGEVDWETAYFIR.H |
|  |  |  |  |  |  |  |  |  |  |  | R.EMLDVYIGQMVSLAER.L |
|  |  |  |  |  |  |  |  |  |  |  | R.IFVNLGDQLEVMSGGAYR.S |
|  |  |  |  |  |  |  |  |  |  |  | R.HRPANNVADFPEIPPATR.E |
|  |  |  |  |  |  |  |  |  |  |  | K.FAMYPACPRPDLLWGLR.A |
|  |  |  |  |  |  |  |  |  |  |  | K.FAMYPACPRPDLLWGLR.A + Oxidation (M) |
|  |  |  |  |  |  |  |  |  |  |  | R.LSVATFYNPGAEAVVAPAPTAR.Q |
|  |  |  |  |  |  |  |  |  |  |  | R.AHTDAGGIILLLQDDVVGGLEFFR.G |
|  |  |  |  |  |  |  |  |  |  |  | R.AHTDAGGIILLLQDDVVGGLEFFRGDR.E |
|  |  |  |  |  |  |  |  |  |  |  | R.LHEACKDWGFFWVDSHGVDAALMEEVK.R |
| NR137 | Membrane steroid-binding protein | gi\|326533634 | 23.98/4.65 | 24.15/4.75 | 1 | 10% | 76 | *Hordeum vulgare* | 2.22 | 2.45 | R.EEPEAEPLPPPVQLGEVDEEELR.Q |
| NR997 | Peroxidase | gi\|129806 | 33.41/6.07 | 33.68/6.17 | 10 | 16% | 510 | *Hordeum vulgare* | 2.84 | 5.4 | K.TGTQGQIR.L |
|  |  |  |  |  |  |  |  |  |  |  | K.SGVMAAVTSDPR.M |
|  |  |  |  |  |  |  |  |  |  |  | K.QTVSCADILTVAAR.D |
|  |  |  |  |  |  |  |  |  |  |  | R.DSVVALGGPSWTVPLGR.R |
|  |  |  |  |  |  |  |  |  |  |  | R.DSVVALGGPSWTVPLGR.R + Oxidation (HW) |
|  |  |  |  |  |  |  |  |  |  |  | R.IYGGDTNINAAYAASLR.A |
|  |  |  |  |  |  |  |  |  |  |  | R.NFASNPAAFSSSFTTAMIK.M |
|  |  |  |  |  |  |  |  |  |  |  | R.NFASNPAAFSSSFTTAMIK.M + 2 Deamidated (NQ) |
|  |  |  |  |  |  |  |  |  |  |  | K.GGLNTVDMVALSGAHTIGQAQCSTFR.A |
|  |  |  |  |  |  |  |  |  |  |  | R.LHFHDCFVQGCDASVLLSGMEQNAIPNAGSLR.G + Oxidation (M) |
| NR287 | Beta-1,3-glucanase 2a | gi\|51860173 | 34.61/4.39 | 33.45/4.29 | 3 | 11% | 176 | *Hordeum vulgare* | 1.08 | 2.89 | K.HFGLFNPDKSPAYPISF.- |
|  |  |  |  |  |  |  |  |  |  |  | R.SAAVAWVQTNVQAHQGLNIK.Y |
|  |  |  |  |  |  |  |  |  |  |  | R.SAAVAWVQTNVQAHQGLNIK.Y + 2 Oxidation (HW) |
| NR275 | Beta-1,3-glucanase 2a | gi\|51860173 | 34.61/4.39 | 32.65/4.38 | 1 | 5% | 80 | *Hordeum vulgare* | 1.85 | 3.02 | R.SAAVAWVQTNVQAHQGLNIK.Y |
| **Stress** | | | | | | | | | | |  |
| NR266 | Glutathione transferase f4 | gi\|326524323 | 25.31/5.44 | 27.54/6.78 | 3 | 23% | 167 | *Hordeum vulgare* | 1.75 | 2.93 | R.SGGDHRQPDHLTR.N |
|  |  |  |  |  |  |  |  |  |  |  | R.VLVCLEEAGAEYEIVPMSR.S + Oxidation (M) |
|  |  |  |  |  |  |  |  |  |  |  | R.NPFGEIPVLEDGDLTLYQSR.A |
| NR176 | Glutathione s-transferase 3-like | gi\|326490728 | 23.56/5.67 | 26.14/5.85 | 5 | 33% | 456 | *Hordeum vulgare* | 1.05 | 2.42 | K.AWWDEISARPAWAK.T |
|  |  |  |  |  |  |  |  |  |  |  | K.AWWDEISARPAWAK.T + 2 Oxidation (HW) |
|  |  |  |  |  |  |  |  |  |  |  | K.LEVWLEVESHHFYPPVR.A |
|  |  |  |  |  |  |  |  |  |  |  | R.VATVLNELGLDFEFVSVDLR.T |
|  |  |  |  |  |  |  |  |  |  |  | K.LNPFGQIPALQDGDEVVFESR.A |
| NR167 | Glutathione s-transferase-like | gi\|326510031 | 23.99/5.47 | 25.21/5.58 | 2 | 25% | 154 | *Hordeum vulgare* | 2.43 | 1.35 | K.YCVGDEVHLGDVFLAPQIHAAINR.F |
|  |  |  |  |  |  |  |  |  |  |  | K.ALDLQIANIVCSSIQPLQGYGVIGLHEGR.L |
| NR98 | Cold-regulated protein | gi\|326511755 | 17.66/4.93 | 17.84/5.10 | 3 | 49% | 232 | *Hordeum vulgare* | 0.58 | 0.33 | K.DISPPLEWYGVPGGAR.S |
|  |  |  |  |  |  |  |  |  |  |  | K.GLPEGFSGAGGNANAGGEGGLQEGVNDWKQPGWR.G |
|  |  |  |  |  |  |  |  |  |  |  | R.SLALVVQDIDADERVPWTHWVVANISPEEK.G |
| NR1153 | Thioredoxin-like protein 1 | gi\|326514956 | 21.08/4.8 | 21.54/4.98 | 2 | 16% | 92 | *Hordeum vulgare* | ↓ | 0.78 | K.LHSALFKGPEEEGPK.T |
|  |  |  |  |  |  |  |  |  |  |  | K.QGYREDEGLHLASDSDEQLLIYIPFMQVIK.L |
| **Protein** | | | | | | | | | | |  |
| NR390 | Guanine nucleotide-binding protein subunit beta-like protein a-like | gi\|326491885 | 36.66/5.97 | 37.15/5.99 | 7 | 41% | 423 | *Hordeum vulgare* | 1.95 | 3.15 | R.FSPNNFAPTIVSGSWDR.S |
|  |  |  |  |  |  |  |  |  |  |  | R.LYSLDAGSIINSLCFSPNR.Y |
|  |  |  |  |  |  |  |  |  |  |  | K.YTIGGDLGGGEGHTGWVSCVR.F |
|  |  |  |  |  |  |  |  |  |  |  | R.GHNDVVTAIATPIDNSPFIVSSSR.D |
|  |  |  |  |  |  |  |  |  |  |  | K.SLLVWDLTNPIQATQDTSSEYGVPFR.R |
|  |  |  |  |  |  |  |  |  |  |  | R.DKSLLVWDLTNPIQATQDTSSEYGVPFR.R |
|  |  |  |  |  |  |  |  |  |  |  | R.LTGHGHFVQDVVLSSDGQFALSGSWDGELR.L |
| NR85 | Acidic ribosomal protein | gi\|326534206 | 12.14/4.43 | 14.51/4.58 | 3 | 21% | 192 | *Hordeum vulgare* | 0.47 | 0.52 | M.GVFTFVCR.D |
|  |  |  |  |  |  |  |  |  |  |  | K.GELEASAATPYDLQR.Q |
|  |  |  |  |  |  |  |  |  |  |  | K.QHKGELEASAATPYDLQR.Q |
| **Others** | | | | | | | | | | |  |
| NR1030 | 819302 protein | gi\|326521236 | 14.62/5.04 | 14.95/5.25 | 3 | 28% | 191 | *Hordeum vulgare* | 1.23 | 0.68 | K.KPLFFYVNLAK.R |
|  |  |  |  |  |  |  |  |  |  |  | K.TENFDELMAAAAEER.E |
|  |  |  |  |  |  |  |  |  |  |  | K.TENFDELMAAAAEEREVAAAEDGEEQA.- |
|  |  |  |  |  |  |  |  |  |  |  |  |
| a The number of spot in gels indicates differentially expressed protein as given in Fig. S2 | | | | | | | | | | | |
| b Names and species of the proteins obtained via the MASCOT software from the NCBInr database | | | | | | | | | | | |
| c Accession number from the NCBInr database; | | | | | | | | | | | |
| d Theoretical molecular weight and isoelectric point | | | | | | | | | | | |
| e Experimental molecular weight and isoelectric point | | | | | | | | | | | |
| f Number of query matced pettides | | | | | | | | | | | |
| g Sequence coverage | | | | | | | | | | | |
| h Statistical probability of the predicted protein is calculated by MASCOT | | | | | | | | | | | |
| i The fold change is calculated using the mean value of each spot in normalized spot volume from gels comparing under control and waterlogging conditions.↑ and ↓ indicate specifically expressed protein under control and waterlogging conditions, respectively. | | | | | | | | | | | |

| **Table S5** The differentially expressed proteins of seminal roots between TF57 and TF58 under control and waterlogging stress | | | | | | | | | | | |
| --- | --- | --- | --- | --- | --- | --- | --- | --- | --- | --- | --- |
|  |  |  |  |  |  |  |  |  |  |  |  |
| **Spot No** | **Protein Name** | **Protein ID^a^** | **Theoretical Mr(kDa)/p*I^b^*** | **Experimental Mr(kDa)/p*I^b^*** | **Matched peptides^c^** | **Cov (%)^d^** | **Score^e^** | **Species** | **Change folds** | | **Peptides Squence** |
|  |  |  |  |  |  |  |  |  | **TF57** | **TF58** |  |
| **Energy** | | | | | | | | | | |  |
| SR270 | Pyrophosphate--fructose 6-phosphate 1-phosphotransferase subunit beta-like | gi\|326522238 | 61.21/6.34 | 63.54/6.45 | 11 | 24% | 369 | *Hordeum vulgare* | 3.76 | 2.31 | K.YYHFVR.L |
|  |  |  |  |  |  |  |  |  |  |  | R.DKIETPEQFK.Q |
|  |  |  |  |  |  |  |  |  |  |  | R.GQSHFFGYEGR.C |
|  |  |  |  |  |  |  |  |  |  |  | R.NQGGFDMICSGR.D |
|  |  |  |  |  |  |  |  |  |  |  | R.IAHMLPLPSVLR.S |
|  |  |  |  |  |  |  |  |  |  |  | K.FASMRDEWAIK.N |
|  |  |  |  |  |  |  |  |  |  |  | K.IYSEMIGNVMTDAR.S |
|  |  |  |  |  |  |  |  |  |  |  | K.YVELTADFVYPYR.N |
|  |  |  |  |  |  |  |  |  |  |  | K.GKYVELTADFVYPYR.N |
|  |  |  |  |  |  |  |  |  |  |  | K.TIQEQLLLERDPHGNVQVAK.I |
|  |  |  |  |  |  |  |  |  |  |  | K.IGVVLSGGQAPGGHNVICGIFDYLQER.A |
| SR565 | Pyruvate decarboxylase | gi\|326528981 | 65.83/5.62 | 65.89/5.68 | 9 | 31% | 775 | *Hordeum vulgare* | 2.32 | 4.13 | R.DPVPFFLAPR.M |
|  |  |  |  |  |  |  |  |  |  |  | R.ILHHTIGVPDFSQELR.C |
|  |  |  |  |  |  |  |  |  |  |  | K.DSLCFIEVIAHKDDTSK.E + Oxidation (HW) |
|  |  |  |  |  |  |  |  |  |  |  | R.IFVPEGHPLKGEANEPLR.V |
|  |  |  |  |  |  |  |  |  |  |  | R.LVGCCNELNAGYAADGYAR.A |
|  |  |  |  |  |  |  |  |  |  |  | R.VIACIGDGSFQVTAQDVSTMLR.C |
|  |  |  |  |  |  |  |  |  |  |  | R.ESKPVYLSISCNLPGLPHPTFTR.D |
|  |  |  |  |  |  |  |  |  |  |  | R.LVQVGVSDVFAVPGDFNLTLLDHLVAEPGLR.L |
|  |  |  |  |  |  |  |  |  |  |  | R.CFQTVTCHQAVVTNLDDAHEQIDTAIATALR.E |
| SR724 | 6-phosphogluconolactonase chloroplastic-like | gi\|326503206 | 28.67/5.53 | 32.54/6.02 | 2 | 9% | 84 | *Hordeum vulgare* | 0.35 | 0.89 | K.WHVFWVDER.V |
|  |  |  |  |  |  |  |  |  |  |  | R.GAFTVVLSGGSLIHALR.K |
| SR82 | Germin-like protein 8-9-like | gi\|326503884 | 24.89/6.58 | 25.48/6.68 | 3 | 25% | 327 | *Hordeum vulgare* | 0.3 | 0.35 | R.IDYAPLGQNPPHTHPR.A |
|  |  |  |  |  |  |  |  |  |  |  | K.NTIDWLQAQFWENNHN. |
|  |  |  |  |  |  |  |  |  |  |  | R.ATEILTVLEGTLYVGFVTSNLPAPNR.N |
| **Lipid metabolism** | | | | | | | | | | |  |
| SR373 | Enoyl-acp reductase | gi\|326494026 | 36.87/6.04 | 37.15/6.12 | 4 | 15% | 242 | *Hordeum vulgare* | 0.42 | 0.96 | R.GKFDESR.K |
|  |  |  |  |  |  |  |  |  |  |  | K.MIEYSYVNAPLQK.E |
|  |  |  |  |  |  |  |  |  |  |  | R.AFIAGVADDNGYGWAIAK.A |
|  |  |  |  |  |  |  |  |  |  |  | K.ALAAAGAEILVGTWVPALNIFETSLR.R |
| SR67 | Quinone reductase 2 of the maize glutamine synthetase complexed with amppnp and methionine sulfoximine | gi\|326512488 | 21.69/5.94 | 22.38/6.12 | 4 | 39% | 271 | *Hordeum vulgare* | 0.14 | 0.52 | K.AFFDATGGLWR.E |
|  |  |  |  |  |  |  |  |  |  |  | K.LFDMDKVQGGSPYGAGTFAADGSR.W |
|  |  |  |  |  |  |  |  |  |  |  | K.LDAPIITPQELADADGILFGFPTR.F |
|  |  |  |  |  |  |  |  |  |  |  | R.WPSEMELEHAFHQGQYFAGIAK.K |
| SR17 | Quinone reductase 2 | gi\|326516502 | 21.77/6.21 | 25.47/6.57 | 6 | 54% | 765 | *Hordeum vulgare* | 0.52 | 0.5 | K.AFFDATGGLWR.E |
|  |  |  |  |  |  |  |  |  |  |  | K.VWQVPEILNEEVLGK.M |
|  |  |  |  |  |  |  |  |  |  |  | R.WPSEMELEHAFHQGK.Y |
|  |  |  |  |  |  |  |  |  |  |  | M.AVKVYVVYYSMYGHVAK.L + Oxidation (M) |
|  |  |  |  |  |  |  |  |  |  |  | K.TDVPVISPQELAEADGVLFGFPTR.F |
|  |  |  |  |  |  |  |  |  |  |  | K.MGAPPKTDVPVISPQELAEADGVLFGFPTR.F |
| **Hormone metabolism** | | | | | | | | | | |  |
| SR160 | 1-aminocyclopropane-1-carboxylate oxidase 1-like | gi\|397740906 | 34.80/5.17 | 36.78/5.12 | 10 | 51% | 1060 | *Hordeum vulgare* | 2.32 | 5.65 | K.AVSGEVDWETAYFIR.H |
|  |  |  |  |  |  |  |  |  |  |  | R.EMLDVYIGQMVSLAER.L |
|  |  |  |  |  |  |  |  |  |  |  | R.IFVNLGDQLEVMSGGAYR.S |
|  |  |  |  |  |  |  |  |  |  |  | R.HRPANNVADFPEIPPATR.E |
|  |  |  |  |  |  |  |  |  |  |  | K.FAMYPACPRPDLLWGLR.A |
|  |  |  |  |  |  |  |  |  |  |  | K.FAMYPACPRPDLLWGLR.A + Oxidation (M) |
|  |  |  |  |  |  |  |  |  |  |  | R.LSVATFYNPGAEAVVAPAPTAR.Q |
|  |  |  |  |  |  |  |  |  |  |  | R.AHTDAGGIILLLQDDVVGGLEFFR.G |
|  |  |  |  |  |  |  |  |  |  |  | R.AHTDAGGIILLLQDDVVGGLEFFRGDR.E |
|  |  |  |  |  |  |  |  |  |  |  | R.LHEACKDWGFFWVDSHGVDAALMEEVK.R |
| SR142 | Isoflavone reductase homolog | [gi\|474219963](http://www.matrixscience.com/cgi/master_results.pl?file=..%2Fdata%2F20150807%2FFTTAoGHaS.dat;sessionID=guest_guestsession#Hit1) | 24.24/6.45 | 33.42/5.42 | 1 | 75% | 162 | *Triticum urartu* | 0.54 | 0.36 | K.QIQEAPIPMNIIFSIGHASYIK.G |
| **N-metabolism** | | | | | | | | | | |  |
| SR171 | Glutamine synthetase isoform GS1_2 | gi\|417356961 | 39.04/5.71 | 39.45/5.78 | 7 | 43% | 500 | *Hordeum vulgare* | 1.84 | 2.14 | R.HMQHIAAYGEGNER.R |
|  |  |  |  |  |  |  |  |  |  |  | K.VIVEYLWVGGSGIDIR.S |
|  |  |  |  |  |  |  |  |  |  |  | K.VAAEETWYGIEQEYTLLQK.D |
|  |  |  |  |  |  |  |  |  |  |  | R.RPASNMDPYVVTSMIAETTLLL.- |
|  |  |  |  |  |  |  |  |  |  |  | R.GDNILVMCDCYTPQGVPIPTNKR.H |
|  |  |  |  |  |  |  |  |  |  |  | K.DVNWPLGWPIGGYPGPQGPYYCAAGADK.A |
|  |  |  |  |  |  |  |  |  |  |  | K.WNYDGSSTGQAPGEDSEVILYPQAIFKDPFR.R |
| **Nucleotide metabolism** | | | | | | | | | | |  |
| SR525 | Soluble inorganic pyrophosphatase | gi\|326516184 | 24.69/5.59 | 27.54/5.65 | 12 | 61% | 612 | *Hordeum vulgare* | 0.36 | 0.3 | K.EVAVDAFLPATTAR.E |
|  |  |  |  |  |  |  |  |  |  |  | K.NENKEVAVDAFLPATTAR.E |
|  |  |  |  |  |  |  |  |  |  |  | R.VLYSSVVYPHNYGFIPR.T |
|  |  |  |  |  |  |  |  |  |  |  | R.EAIQYSMDLYAQYILQSLR.Q |
|  |  |  |  |  |  |  |  |  |  |  | R.EAIQYSMDLYAQYILQSLRQ.- |
|  |  |  |  |  |  |  |  |  |  |  | R.EAIQYSMDLYAQYILQSLRQ.- + Oxidation (M) |
|  |  |  |  |  |  |  |  |  |  |  | K.IIAVCADDPEYRHYNDISELSPHR.L + Deamidated (NQ); 2 Oxidation (HW) |
|  |  |  |  |  |  |  |  |  |  |  | K.IIAVCADDPEYRHYNDISELSPHR.L + Deamidated (NQ); 2 Oxidation (HW) |
|  |  |  |  |  |  |  |  |  |  |  | R.AVAAHPWHDLEIGPGAPAVFNVVVEITK.G |
|  |  |  |  |  |  |  |  |  |  |  | R.TLCEDNDPMDVLVLMQEPVIPGSFLR.A |
|  |  |  |  |  |  |  |  |  |  |  | R.TLCEDNDPMDVLVLMQEPVIPGSFLR.A + Oxidation (M) |
|  |  |  |  |  |  |  |  |  |  |  | R.RAVAAHPWHDLEIGPGAPAVFNVVVEITK.G |
| **Stress** | | | | | | | | | | |  |
| SR314 | Germin-like protein | gi\|9837119 | 20.39/7.15 | 24.24/7.11 | 3 | 29% | 302 | *Hordeum vulgare* | 0.35 | 0.42 | R.IDYGPLGVNTPHIHPR.A |
|  |  |  |  |  |  |  |  |  |  |  | K.LIDWLQSQFWENNHY.- + Deamidated (NQ) |
|  |  |  |  |  |  |  |  |  |  |  | R.ATELLTVLEGTLYLGFVTSNPNR.L |
| SR101 | Glutathione transferase f4 | gi\|326524323 | 25.31/5.44 | 27.14/6.55 | 3 | 23% | 165 | *Hordeum vulgare* | 0.98 | 2.94 | R.SGGDHRQPDHLTR.N |
|  |  |  |  |  |  |  |  |  |  |  | R.VLVCLEEAGAEYEIVPMSR.S + Oxidation (M) |
|  |  |  |  |  |  |  |  |  |  |  | R.NPFGEIPVLEDGDLTLYQSR.A |
| SR86 | Glutathione transferase | gi\|326532030 | 25.01/5.82 | 25.15/5.95 | 1 | 9% | 198 | *Hordeum vulgare* | 0.97 | 3.17 | R.NPFGQIPAFQDGDLLLFESR.A |
| SR328 | Cold-regulated protein | gi\|326511755 | 17.66/4.93 | 16.98/4.98 | 3 | 49% | 232 | *Hordeum vulgare* | 0.51 | 0.37 | K.DISPPLEWYGVPGGAR.S |
|  |  |  |  |  |  |  |  |  |  |  | K.GLPEGFSGAGGNANAGGEGGLQEGVNDWKQPGWR.G |
|  |  |  |  |  |  |  |  |  |  |  | R.SLALVVQDIDADERVPWTHWVVANISPEEK.G |
| SR583 | r40c1 protein | gi\|326497973 | 39.06/6.27 | 18.54/6.34 | 3 | 18% | 184 | *Hordeum vulgare* | 0.95 | 0.28 | K.ILPWGEEAYAGGSANAPR.G |
|  |  |  |  |  |  |  |  |  |  |  | R.DGNVVLAPSNPRDEHQHWFK.D |
|  |  |  |  |  |  |  |  |  |  |  | K.DHGGVHDGTTVVLWEWAKGDNQSWK.I + 2 Deamidated (NQ); 3 Oxidation (HW) |
| SR781 | Thioredoxin-like protein 1 | gi\|326514956 | 21.08/4.8 | 22.54/4.98 | 4 | 16% | 92 | *Hordeum vulgare* | 0.53 | 0.59 | K.GLAFSEAERDAHYLR.G |
|  |  |  |  |  |  |  |  |  |  |  | R.ATGDEYHELLQEFMTAVK.Q |
|  |  |  |  |  |  |  |  |  |  |  | K.VLVQFEDFANHNAFDLLAK.Y |
|  |  |  |  |  |  |  |  |  |  |  | R.RATGDEYHELLQEFMTAVK.Q |
| **RNA** | | | | | | | | | | |  |
| SR445 | Transcription factor btf3 | gi\|326504174 | 19.19/5.99 | 20.15/5.85 | 3 | 28% | 349 | *Hordeum vulgare* | 0.3 | 0.9 | K.LQDLLPTIINQLGPDNLDNLR.R |
|  |  |  |  |  |  |  |  |  |  |  | K.LQDLLPTIINQLGPDNLDNLRR.L |
|  |  |  |  |  |  |  |  |  |  |  | R.VGVNTIPGIEEVNIFKDDVVIQFQNPK.V |
| **Protein** | | | | | | | | | | |  |
| SR434 | Subtilisin-like serine protease | gi\|326497905 | 80.33/6.26 | 78.54/6.28 | 2 | 6% | 91 | *Hordeum vulgare* | 0.35 | 0.28 | K.ATNYGENIIIGMVDTGVWPESR.S |
|  |  |  |  |  |  |  |  |  |  |  | K.GASFFTAAQYIVDNGGSGLISSLR.I |
|  |  |  |  |  |  |  |  |  |  |  |  |
| **Others** | | | | | | | | | | |  |
| SR522 | Bark storage protein a-like | gi\|326503550 | 39.46/5.9 | 18.54/5.97 | 4 | 15% | 276 | *Hordeum vulgare* | 0.42 | 0.35 | K.SIPNMDIQGR.R |
|  |  |  |  |  |  |  |  |  |  |  | K.SIPNMDIQGR.R + Oxidation (M) |
|  |  |  |  |  |  |  |  |  |  |  | R.FGDGKDNELPLEAAGDYTR.E |
|  |  |  |  |  |  |  |  |  |  |  | R.AGPFVGLVVPNTYEMVPVLESPSFVASK.S |
|  |  |  |  |  |  |  |  |  |  |  |  |
| a The number of spot in gels indicates differentially expressed protein as given in Fig. S2 | | | | | | | | | | | |
| b Names and species of the proteins obtained via the MASCOT software from the NCBInr database | | | | | | | | | | | |
| c Accession number from the NCBInr database; | | | | | | | | | | | |
| d Theoretical molecular weight and isoelectric point | | | | | | | | | | | |
| e Experimental molecular weight and isoelectric point | | | | | | | | | | | |
| f Number of query matced pettides | | | | | | | | | | | |
| g Sequence coverage | | | | | | | | | | | |
| h Statistical probability of the predicted protein is calculated by MASCOT | | | | | | | | | | | |
| i The fold change is calculated using the mean value of each spot in normalized spot volume from gels comparing under control and waterlogging conditions.↑ and ↓ indicate specifically expressed protein under control and waterlogging conditions, respectively. | | | | | | | | | | | |
